# Co-silencing of ABA receptors (SlRCAR) reveals interactions between ABA and ethylene signaling during tomato fruit ripening

**DOI:** 10.1093/hr/uhac057

**Published:** 2022-06-05

**Authors:** Jian Zou, Ning Li, Nan Hu, Ning Tang, Haohao Cao, Yudong Liu, Jing Chen, Wei Jian, Yanqiang Gao, Jun Yang, Zhengguo Li

**Affiliations:** 1 Key Laboratory of Plant Hormones and Development Regulation of Chongqing, School of Life Sciences, Chongqing University, Chongqing 401331, China; 2 Key Laboratory of Southwest China Wildlife Resources Conservation (Ministry of Education), School of Life Science, China West Normal University, Nanchong, Sichuan 637009, China; 3 School of Life Sciences, Henan Normal University, Xinxiang, Henan 453007, China; 4 College of Biology and Food Engineering, Anyang Institute of Technology, Anyang 455000, China; 5 Collaborative Innovation Center of Special Plant Industry in Chongqing, Institute of Special Plants, Chongqing University of Arts and Sciences, Yongchuan, Chongqing 402160, China

## Abstract

The ripening of fleshy fruits is highly dependent on the regulation of endogenous hormones, including ethylene, abscisic acid (ABA) and other phytohormones. However, the regulatory mechanism of ABA signaling and its interaction with ethylene signaling in fruit ripening are still unclear. In this study, multi-gene interference (RNAi) was applied to silence the ABA receptor genes in tomato for screening the specific receptors that mediate ABA signaling during fruit ripening. The results indicated that the ABA receptors, including SlRCAR9, SlRCAR12, SlRCAR11, and SlRCAR13, participate in the regulation of tomato fruit ripening. Comparative analysis showed that *SlRCAR11* and *SlRCAR13* play more important roles in mediating ABA signaling during tomato fruit ripening. Co-silencing of the four genes encoding these receptors could weaken the ethylene biosynthesis and signaling pathway at the early stage of tomato fruit ripening, leading to delayed fruit ripening. Meanwhile, co-silencing enhanced fruit firmness, and altered the shelf-life and susceptibility to *Botrytis cinerea* of the transgenic fruits. Furthermore, blocking ABA signaling did not affect the ability of ethylene to induce fruit ripening, whereas the block may inhibit the effectiveness of ABA in promoting fruit ripening. These results suggested that ABA signaling may be located upstream of ethylene signaling in regulating fruit ripening. Our findings provide a new insight into the complex regulatory network of phytohormones in regulating fruit ripening in tomato.

## Introduction

The ripening of fleshy fruits is an important process in higher plants, and could affect not only the seed development and reproduction of offspring in plants, but also the quality changes and nutritional composition of fruits. As a complex developmental process, fruit ripening is generally described as the coordinated manifestation of changes in color, texture, flavor, and nutritional characteristics, and could be influenced by internal and external factors [1, 2]. In general, ethylene is considered an important phytohormone in regulating fruit ripening, and it plays a crucial role as a trigger in the ripening of climacteric fruits. However, given the complexity of fruit ripening, the pre-ripening action of ethylene often requires the synergy of other hormones [3–5]. For example, ethylene could interact with auxin to regulate fruit ripening by a possible ethylene–auxin balance mechanism in tomato, banana, and grape [6, 7]. As another important hormone for regulating fruit ripening, abscisic acid (ABA) can function in non-climacteric and climacteric fruits [8–11]. In tomato, several studies have indicated that exogenous ABA application could significantly accelerate fruit ripening [12], whereas ABA deficiency produced by function loss of key genes related to ABA biosynthesis could severely inhibit fruit pigmentation and softening during fruit ripening in *not*/*flc* and *hp3* mutants [13, 14]. In addition, Sun *et al*. [10, 15] reported that silencing the *SlNCED1* gene could decrease the endogenous ABA content, thereby causing physiological alterations during fruit ripening, including carotenoid accumulation, fruit firmness, and other related physiological characteristics.

However, the synergistic relationship between ethylene and ABA in the regulation of fruit ripening remains unknown. Several previous independent studies have provided insights into the correlation between ABA and ethylene signals in the ripening of climacteric and non-climacteric fruits. For banana, ABA treatment could slightly promote the production of ethylene and enhance ethylene sensitivity [16]. Zaharah *et al*. [17] reported that endogenous ABA was raised prior to the peak of ethylene production and respiration rate in mango. In strawberry, ABA treatment could improve fruit pigment, reduce fruit firmness, and accelerate ethylene production [18]. In tomato fruit, the application of exogenous ABA accelerated the production of and response to ethylene by affecting the expression of several crucial genes for ethylene biosynthesis and signaling, such as *LeACS4*, *LeACO1*, *GR*, and *LeETR6* [10, 11, 19]. However, knowledge regarding the interaction between ABA signaling and ethylene signaling during fruit ripening in tomato is still lacking. In addition, understanding of fruit ripening-specific ABA signaling is unclear.

In general, ABA receptors (PYL/RCAR) would mediate ABA perception and signaling transduction with other components, including protein phosphatase 2C (PP2C ) and SNF1-related protein kinase 2 (SnRK2) [20–22]. The ABA receptor family in plants contains multiple genes, and they usually present functional divergence, but some may be functionally redundant [20, 22, 23]. To date, 15 ABA receptors have been identified in tomato, and some of these receptors were characterized for drought resistance [24]. However, the members of the ABA receptor family, which regulate fruit ripening by mediating ABA signaling, remain unknown. Therefore, it is essential to explore the fruit-ripening-related ABA receptors, and investigate how ABA signaling affects ethylene biosynthesis and signaling during fruit ripening.

In this study, multi-gene interference (RNAi) vectors were introduced to silence the multi-gene expression of target ABA receptors and screen the receptors that mediate the ABA signal in regulating fruit ripening. The transgenic tomato fruits with a partial defect of ABA signaling that were obtained were used to investigate the relationship between the ABA signal and the ethylene signal in regulating fruit ripening. It was found that co-silencing ABA receptor genes, including *SlRCAR9*, *SlRCAR12*, *SlRCAR11*, and *SlRCAR13*, could delay the ripening of tomato fruit, and *SlRCAR11* and *SlRCAR13* were shown to have comparatively more important roles. In addition, our findings suggested ABA-regulated ripening of tomato fruit would depend on ethylene signaling, and ABA would be located upstream of ethylene signaling at the early stage of fruit ripening in tomato.

**Figure 1:**
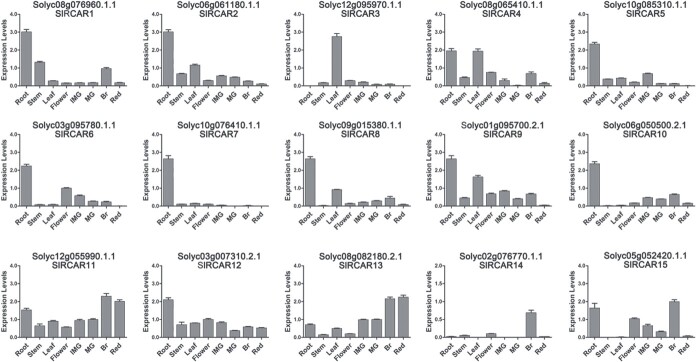
Relative expression of ABA receptor genes in different tissues of WT tomato. Tomato tissues included root, stem, leaf, flower, immature green fruit (IMG), mature green fruit (MG), breaker fruit (Br), and full red fruit (Red). Values represent mean ± standard deviation.

**Figure 2:**
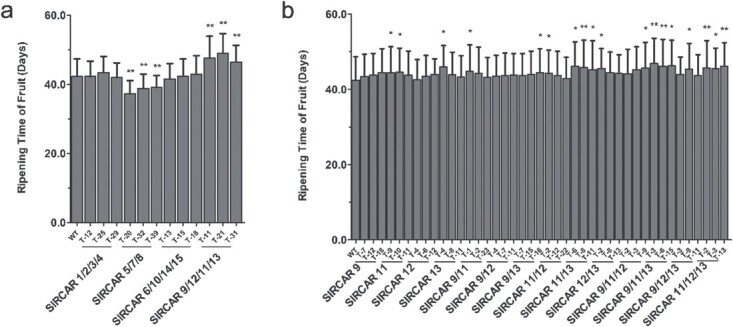
Fruit ripening time of transgenic and wild-type tomato. **a** Fruit ripening time of four-gene-silenced tomatoes, comprising SlRCAR1-2-3-4, SlRCAR5-7-8, SlRCAR6–1014-15, and SlRCAR9-12-11-13. **b** Fruit ripening time of single-gene, double-gene, and three-gene silenced tomato, comprising SlRCAR9, SlRCAR12, SlRCAR11, SlRCAR13, SlRCAR9-11, SlRCAR9-12, SlRCAR9-13, SlRCAR11-12, SlRCAR11-13, SlRCAR12-13, SlRCAR9-11-12, SlRCAR9-11-13, SlRCAR9-12-13, and SlRCAR11-12-13. Values represent mean ± standard deviation. ^*^P < .05; ^**^P < .01 by *t*-test (*n* = 30).

## Results

### Phylogenetic analysis and tissue expression pattern of abscisic acid receptor genes in tomato

Information for 15 known ABA receptor candidate genes was obtained from the annotated tomato genome database (SOL Genomics Network, http://solgenomics.net/) to explore the function of tomato ABA receptors in the regulation of fruit ripening, and they were numbered from *SlRCAR1* to *SlRCAR15* (Supplementary Table S1). Then, phylogenetic analysis of these 15 ABA receptor (PYR/PYL/RCAR) sequences, together with all members of the PYR/PYL/RCAR family from other species, was constructed to investigate their relationship (Supplementary Appendix 3 and Supplementary Figure S1). The results showed that these candidates could be categorized into three subfamilies, matching the corresponding groups of PYR/PYL/RCAR receptors from other species, except for Solyc02g076770.1.1 (*SlRCAR14*). In subfamily I, Solyc08g076960.1.1 (*SlRCAR1*) and Solyc06g061180.1.1 (*SlRCAR2*) were closely related to *Arabidopsis* PYL1/PYR1, whereas Solyc12g095970.1.1 (*SlRCAR3*) and Solyc08g065410.1.1 (*SlRCAR4*) were more closely related to AtPYL2/PYL3. In subfamily II, six tomato receptors related to AtPYL4/PYL5/PYL6 were found, including Solyc10g085310.1.1 (*SlRCAR5*), Solyc10g076410.1.1 (*SlRCAR7*), Solyc09g015380.1.1 (*SlRCAR8*), Solyc03g095780.1.1 (*SlRCAR6*), Solyc06g050500.2.1 (*SlRCAR10*), and Solyc05g052420.1.1 (*SlRCAR15*). In subfamily III, four tomato receptors, comprising Solyc01g095700.2.1 (*SlRCAR9*), Solyc12g055990.1.1 (*SlRCAR11*), Solyc03g007310.2.1 (*SlRCAR12*), and Solyc08g082180.2.1 (*SlRCAR13*), were closely correlated to AtPYL7/PYL8/PYL9/PYL10. The ABA receptor Solyc02g076770.1.1 (*SlRCAR14*) was ungrouped because it showed no obvious relation to *Arabidopsis* ABA receptors.

Subsequently, the transcriptional profiling of these 15 candidate ABA receptor genes in different tissues was conducted by qRT–PCR (Fig. 1). The results showed that nine ABA receptor genes presented their expression peak in the root, including *SlRCAR1*, *SlRCAR2*, *SlRCAR5*, *SlRCAR6*, *SlRCAR7*, *SlRCAR8*, *SlRCAR9*, *SlRCAR10*, and *SlRCAR12*. In addition, *SlRCAR3* showed its highest expression level in the leaf, whereas *SlRCAR4* was highly expressed in the root and leaf. The *SlRCAR11*, *SlRCAR13*, *SlRCAR14*, and *SlRCAR15* genes exhibited relatively high expression levels during fruit development and ripening (Fig. 1). The expression of *SlRCAR11* and *SlRCAR13* gradually increased during fruit development and ripening, and reached the peak value at the breaker stage. Although *SlRCAR14* and *SlRCAR15* genes had low background expression in all tissues, their expression levels reached the maximum at the breaker stage, and the *SlRCAR14* gene was specifically expressed in fruit at the breaker stage. These results implied that these four genes might function in regulating fruit development or fruit ripening.

### Screening of abscisic acid receptor candidate genes associated with fruit ripening

Multiple-gene silencing technology (RNAi technology) was used in this study to facilitate the screening of ABA receptor genes involved in fruit ripening. However, a traditional RNAi vector system, such as pCAMBIA1301, could not achieve simultaneous silencing of multiple genes. Therefore, the vector pCAMBIA1301 was reformed into a multiple-gene RNAi vector system, including pCAMBIA1301m and pCAMBIA1301s, to perform multiple-gene silencing of *SlRCARs* in this study (Supplementary Figure S2 and Supplementary Appendixes 1 and 2).

The 15 tomato ABA receptors were divided into four groups based on the results of phylogenetic analysis and expression pattern analysis, and they were constructed into multiple-gene silencing vectors (Supplementary Figure S2), including *SlRCAR1*-*2*-*3*-*4*, *SlRCAR5*-*7*-*8*, *SlRCAR6*-*10*-*14*-*15*, and *SlRCAR9*-*12*-*11*-*13*. Then, the multiple-gene silenced transgenic tomato plants were obtained, named SlRCAR1-2-3-4; SlRCAR5-7-8, SlRCAR6-10-14-15, and SlRCAR9-12-11-13 (Supplementary Figure S3a). Multiple-gene silencing could reduce ABA signaling by expression inhibition of these ABA receptor genes (Supplementary Figure S3a). The ABA receptor genes related to fruit ripening were screened by analyzing the fruit ripening time of multiple-gene-silenced lines. It was found that the fruit ripening time of SlRCAR9-12-11-13 was significantly prolonged, and all three lines presented consistent results. The fruit ripening time of SlRCAR5-7-8 was shortened, but SlRCAR1-2-3-4 and SlRCAR6-10-14-15 did not alter their fruit ripening time, compared with WT (Fig. 2a; Supplementary Table S5). The results implied that *SlRCAR9*, *SlRCAR12*, *SlRCAR11*, and *SlRCAR13* should be among the genes for ABA receptors that play a role in tomato fruit ripening.

In order to eliminate the gene/genes that was/were not involved in ABA signaling related to fruit ripening among *SlRCAR9*, *SlRCAR12*, *SlRCAR11*, and *SlRCAR13*, we performed single-gene silencing, double-gene silencing, and three-gene silencing. These transgenic plants were labeled SlRCAR9, SlRCAR12, SlRCAR11, SlRCAR13, SlRCAR9-11, SlRCAR9-12, SlRCAR9-13, SlRCAR11-12, SlRCAR11-13, SlRCAR12-13, SlRCAR9-11-12, SlRCAR9-11-13, SlRCAR9-12-13, and SlRCAR11-12-13 (Supplementary Figure S3b). The results indicated that the fruit ripening time of all these transgenic plants was significantly prolonged to a varying degree compared with WT, when the *SlRCAR11* and/or *SlRCAR13* gene was/were silenced (Fig. 2b; Supplementary Table S6). The phenotype of *SlRCAR11/13* double-silenced lines was more obvious than those of single-silenced plants (Fig. 2b; Supplementary Table S6). Furthermore, SlRCAR11-13, SlRCAR9-11-13, and SlRCAR11-12-13 presented longer fruit ripening times than other transgenic plants. These results suggested that *SlRCAR11* and *SlRCAR13* would play a more important role during fruit ripening in tomato than *SlRCAR9*, *SlRCAR12*, and other candidates. However, the fruit ripening time of single-gene silenced, double-gene silenced, and three-gene silenced transgenic tomatoes was shorter than that of four-gene (*SlRCAR9*, *SlRCAR12*, *SlRCAR11*, and *SlRCAR13*) co-silenced transgenic tomatoes. This result implied that the *SlRCAR9* and *SlRCAR12* genes could also be involved, albeit less importantly than *SlRCAR11* and *SlRCAR13*.

### Suppression of *SlRCAR9*, *SlRCAR11*, *SlRCAR12*, and *SlRCAR13* alters fruit ripening time and texture in tomato

As the fruit ripening time of co-silenced *SlRCAR9*, *SlRCAR12*, *SlRCAR11*, and *SlRCAR13* transgenic plants was prolonged—longer than those of single-gene, double-gene, and three-gene silenced transgenic plants (Fig. 2; Supplementary Tables S5 and S6)—the SlRCAR9-12-11-13 transgenic lines were used to explore the regulatory mechanism of ABA signaling by these ABA receptor genes in mediating tomato fruit ripening and to determine the ripening indicator gene/genes that was/were affected by this fruit-ripening-related ABA signaling.

**Figure 3:**
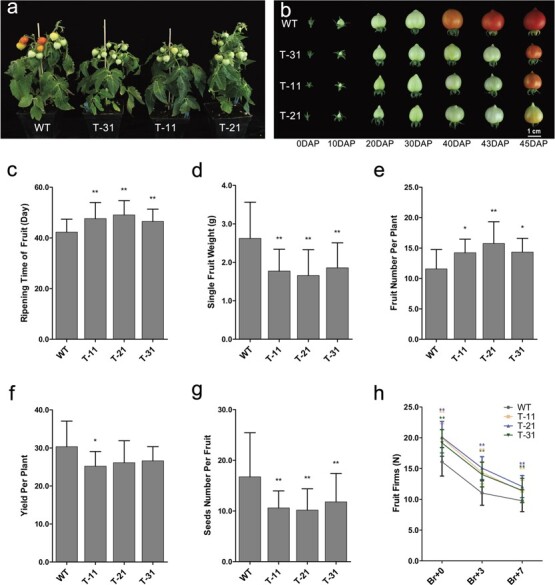
Phenotype of transgenic tomato fruits with four-gene co-silencing. **a** Photographs of whole plants of WT and four-gene co-silenced transgenic plants. **b** Phenotype of fruits at six different stages. **c** Fruit ripening time. **d** Single fruit weight. **e** Fruit number per plant. **f** Yield per plant. **g** Seed number per fruit. **h** Fruit firmness. T-11, T-21, and T-31, transgenic lines; the number of fruits for each group was more than or equal to 30; ^*^P < .05; ^**^P < .01 by *t*-test.

The co-silencing of these four genes prolonged the fruit ripening time by 4–7 days. Phenotypic analysis showed that the fruit ripening time of three SlRCAR9-12-11-13 transgenic lines was 47.60 ± 6.33, 49.05 ± 5.63, and 46.49 ± 4.83 days, times that were longer than that of WT tomato (42.29 ± 5.05 days) (Fig. 3a–c). Furthermore, the co-silencing of these four genes could affect the fruit weight, fruit set, yield per plant, seed number per fruit, and fruit firmness (Fig. 3d–h). The single fruit weight of transgenic tomato was ~29–37% lower than that of WT at the red ripe stage (Fig. 3d), whereas the fruit number per plant was increased by ~23–36% compared with WT (Fig. 3f). Thus, the total yield of transgenic tomato was only slightly reduced, by 12–17% (Fig. 3f). In addition, co-silencing significantly reduced the seed number per fruit in tomato (Fig. 3g) and enhanced fruit firmness (Fig. 3h).

**Figure 4:**
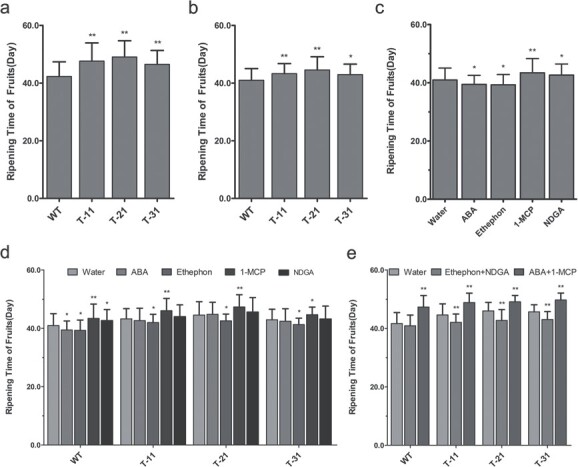
Effects of hormones and hormone inhibitors on fruit ripening time of transgenic tomato by four-gene co-silencing and in WT tomato. **a** Fruit ripening time with no treatment. **b** Fruit ripening time by treatment with double-distilled water. **c** Fruit ripening time by treatment with water, ethephon, ABA, NDGA, and1-MCP. **d** Ripening time by treatment with water, ethephon, ABA, NDGA, and 1-MCP. **e** Ripening time by treatment with water, ethephon + NDGA, and ABA +1-MCP. T-11, T-12, and T-13, transgenic lines; values represent mean ± standard deviation (*n* ≥ 30); ^*^P < .05; ^**^P < .01 by *t*-test.

**Figure 5:**
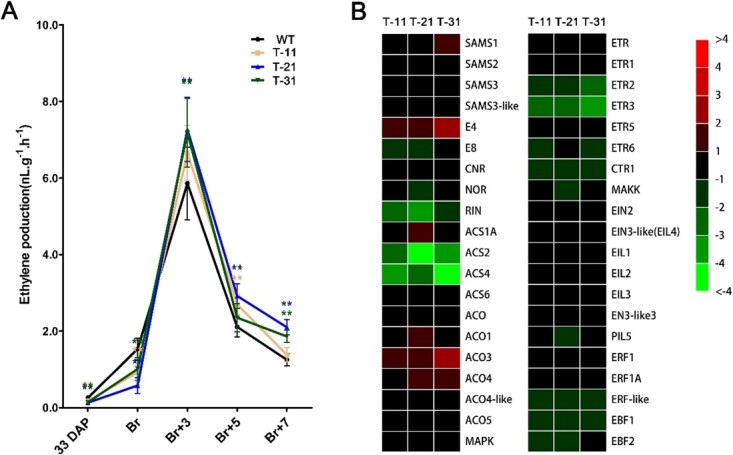
Ethylene production and related gene expression in transgenic tomato fruits by four-gene co-silencing. **a** Ethylene production of fruits in transgenic lines during fruit ripening, using WT as control. The number of fruits for each group was set to 30, and values represent mean ± standard deviation. **b** Relative expression of genes involved in the ethylene biosynthesis and signaling pathway of transgenic fruit at the breaker stage using WT as control. T-11, T-12, T-13, transgenic lines.^**^P < .01 by *t*-test.

**Figure 6:**
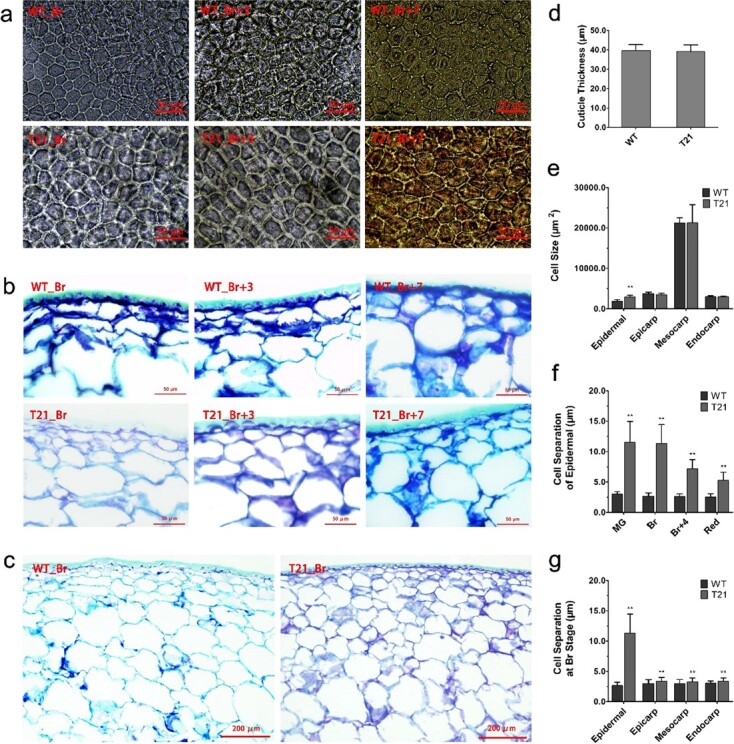
Variation of fruit pericarp cells in transgenic tomato fruits by four-gene suppression. **a** Epidermal cells at inner surface at Br, Br + 3, and Br + 7 stages from identical positions. **b** and **c** Histological analysis of pericarp tissue at Br, Br + 3, and Br + 7 stages. Scale bars = 50 μm (**a** and **b**) and 200 μm (**c**). **d***–***g** Statistical analysis of cuticle thickness (**d**), epidermal cell size (**e**), cell separation of epidermal tissue (**f**), and cell separation of pericarp tissue at Br stage (**g**). T-21, transgenic line; ^**^P < .01 by *t*-test.

**Figure 7:**
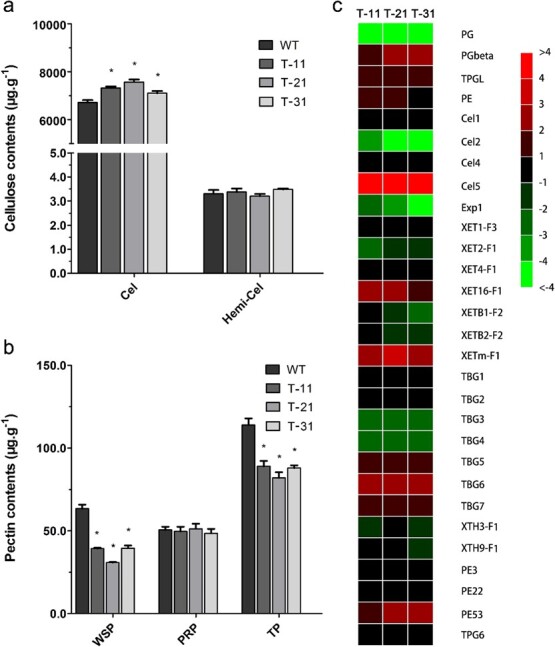
Changes in cell wall catabolism induced by four-gene suppression. **a** and **b** Cell wall components in fruit at the breaker stage. Cel, cellulose; Hemi-Cel, hemicellulose; WSP, water-soluble pectin; PRP, protopectin; TP, total pectin. **c** Expression alteration of genes involved in cell wall catabolism in transgenic fruit at the breaker stage compared with WT. ^*^P < .05 by *t*-test.

### Abscisic acid signaling regulates fruit ripening depending on ethylene signaling

Ethylene is an important hormone for regulating fruit ripening, and serves as a trigger and promoter during ripening of climacteric fruits. Wild-type fruits and transgenic tomatoes (*SlRCAR9-12-11-13*) were treated with ethephon (an ethylene releaser, simulating exogenous ethylene), ABA, and their inhibitors (1-MCP and NDGA) at the mature green stage (33 DPA) to explore the correlation between ABA signal and ethephon signal during fruit ripening (Fig. 4). The application of exogenous ethephon and ABA significantly shortened the fruit ripening time of WT tomato, whereas the ethylene inhibitor 1-MCP and ABA inhibitor NDGA significantly prolonged the ripening time of WT tomato (Fig. 4a–c). The effect of ethylene application in promoting fruit ripening was relatively stronger than that of ABA, whereas the ripening inhibition effect of 1-MCP was stronger than that of NDGA in WT fruits (Fig. 4d). With regard to transgenic tomato fruits, exogenous ethylene and its inhibitor could significantly alter their ripening time, while ABA and its inhibitor could not elicit significant alteration of ripening time (Fig. 4d). These results indicated that ethylene could effectively promote fruit ripening in both WT and transgenic tomato, and its perception inhibitor 1-MCP also repressed the ripening process in both WT and transgenic tomato. In contrast, the promotion of ABA and inhibition of its biosynthesis inhibitor NDGA could only act on WT tomato, but not on transgenic fruit. These results suggested that the simultaneous silencing of *SlRCAR9*, *SlRCAR11*, *SlRCAR12*, and *SlRCAR13* genes would result in a block of ABA signaling, which could lead to the ABA-insensitive phenotype in tomato (Fig. 4d). Nevertheless, the block caused by silencing of these genes did not alter the ethylene sensitivity of tomato fruits.

In addition, ethylene plus NDGA or ABA plus 1-MCP was used to co-treat the transgenic and WT tomato to investigate whether the block of ethylene signaling impacts the regulating capability of ABA during fruit ripening (Fig. 4e). The results showed that co-application of ethylene and NDGA did not significantly alter the fruit ripening time in WT, whereas this co-application significantly shortened the fruit ripening time in transgenic tomato, similar to treatment with ethylene alone (Fig. 4d and e). Moreover, the co-application of ABA and 1-MCP prolonged the fruit ripening time in transgenic and WT tomato similarly to 1-MCP treatment alone. These results suggested that suppression of ethylene signaling caused by 1-MCP also induced ABA insensitivity during fruit ripening in tomato, and further confirmed that the block of ABA signaling could not affect the regulating capability of ethylene during fruit ripening.

### Suppression of *SlRCAR9*, *SlRCAR11*, *SlRCAR12*, and *SlRCAR13* alters ethylene production during fruit ripening in tomato

Ethylene production was tested during fruit ripening to determine whether the regulation of fruit ripening by ABA signaling depended on ethylene, and the expression level of genes associated with ethylene biosynthesis and signaling was measured in transgenic (SlRCAR9-12-11-13) fruits at the breaker stage, using WT as control (Fig. 5). In this experiment, 14 genes related to ethylene biosynthesis, 5 genes involved in ethylene biosynthesis regulation, and 21 ethylene signaling genes were tested. The results showed that the key genes for ethylene biosynthesis, *ACS2* (0.054- to 0.133-fold) and *ACS4* (0.044- to 0.235-fold), were down-regulated compared with control at the breaker stage (Fig. 5b). Moreover, three ethylene biosynthesis-regulating genes were down-regulated, including *RIN* (0.122- to 0.498-fold), *NOR* (0.489- to 0.680-fold) and *E8* (0.274- to 0.669-fold) (Fig. 5b). Furthermore, 9 ethylene signaling genes were down-regulated and the other 12 ethylene signaling genes exhibited insignificant alteration in expression compared with control. The down-regulated ethylene signaling genes comprised *ETR2* (0.207- to 0.272-fold), *ETR3* (0.114- 0.145-fold), *ETR6* (0.354- to 0.459-fold), *CTR1* (0.286- to 0.370-fold), *MAKK* (0.499- to 0.693-fold), *PIL5* (0.469- to 0.0.868-fold), *ERF*-like (0.282- to 0.479-fold), *EBF1* (0.275- to 0.330-fold), and *EBF2* (0.405- to 0.518-fold) (Fig. 5b). The expression alteration of these genes involved in ethylene biosynthesis and ethylene signaling may interrupt ethylene biosynthesis and weaken ethylene signaling. In addition, the ethylene production of transgenic fruits was lower than that of WT at the mature green stage and breaker stage but higher at the Br + 3, Br + 5, and Br + 7 stages compared with WT fruits (Fig. 5a). Moreover, the endogenous ABA content reached its peak prior to ethylene production (Supplementary Figure S4). These results indicated that ABA signaling suppression caused by co-silencing *SlRCAR9*, *SlRCAR11*, *SlRCAR12*, and *SlRCAR13* could decrease ethylene biosynthesis and weaken ethylene signaling at the early stage of fruit ripening, which would affect fruit ripening.

### Suppression of abscisic acid receptor expression alters cell wall catabolism in fruits

In this study, transgenic fruits with co-silencing of *SlRCAR9*, *SlRCAR11*, *SlRCAR12*, and *SlRCAR13* exhibited significant changes in fruit firmness and fruit size. Fruit firmness alteration may be due to changes in cell wall composition and structure, and the change in fruit size may be ascribed to the number and/or size of cells. Histological analysis was performed to analyze the pericarp tissue of transgenic and WT fruits to determine the causes of hard and small fruit formation in transgenic fruits (Fig. 6). The results showed the same tissue structure of pericarp between transgenic and WT fruits (Fig. 6), and no significant difference was observed in cuticle thickness (Fig. 6d). Nevertheless, cell size was increased in epidermal tissue of pericarp, and cell number in one view was decreased in transgenic tomato compared with WT (Fig. 6a–c and e). However, in another part of the pericarp tissue, cell size was insignificantly different between transgenic and WT fruits (Fig. 6a–c and e). Thus, the decrease in cell number might be a cause of small fruit formation in transgenic fruits. Meanwhile, suppression of *SlRCAR9*, *SlRCAR11*, *SlRCAR12*, and *SlRCAR13* caused a significant increase in the cell separation of epidermal tissue at the mature green, breaker, 4 days after breaker, and red ripe stages (Fig. 6a and f). In other pericarp tissue, cell separation in transgenic fruit was also increased at the breaker stage compared with WT (Fig. 6g). The larger cell separation means greater cell wall thickness, which may be an important reason for the firmer fruit in the transgenic tomato.

Cellulose, hemicellulose, and pectin are major components of the cell wall, and their contents and proportions could affect the structure and mechanical support of the cell wall, thereby affecting fruit firmness. To determine whether the thickening of the cell wall was due to the alteration of cell wall components, the major components of the cell wall were measured in transgenic and WT fruits at the breaker stage. The results showed that the cellulose content in transgenic fruits was significantly higher than that in WT, whereas the hemicellulose content was altered insignificantly (Fig. 7a). Compared with WT, the contents of total pectin and WSP in transgenic fruits were decreased, whereas insignificant alteration of the protopectin content was observed (Fig. 7b). The increase in cellulose content and decrease in WSP content could affect the structure and mechanical support of the cell wall, which correspondingly caused the thickening of the cell wall in transgenic fruits.

To examine the molecular mechanism of these changes, the gene expression we measured the levels of cell wall catabolism-related proteins in transgenic and WT fruits, such as endo-β-1,4-glucanases (Cels), xyloglucan endotransglycosylase (XET), xyloglucan endotransglucosylase hydrolase (XTH), expansin (EXP), polygalacturonase (PG), β-galactosidase precursor (TBG), pectin methylesterase (PME), and pectin esterase (PE) families. In the Cel family, the *Cel2* gene was down-regulated, whereas *Cel5* was up-regulated (Fig. 7c). In the XET family, the expression level of *XET2-F1*, *XETB1-F2*, and *XETB2-F2* was down-regulated, whereas *XET16-F1* and *XETmF1* were up-regulated. Regarding the XTH gene, *XTH3-F1* and *XTH9-F1* were down-regulated. Furthermore, an EXP gene, *Exp1*, was down-regulated (Fig. 7c). Within these genes associated with cellulose and hemicellulose catabolism, the number of down-regulated genes was more than that of up-regulated ones (Fig. 7c), which implied that ABA signaling suppression by four-receptor gene (*SlRCAR9*, *SlRCAR11*, *SlRCAR12*, and *SlRCAR13*) silencing could result in a balance break of polysaccharide catabolism, such as cellulose and hemicellulose, during fruit ripening in tomato, which may explain the changes in cellulose and hemicellulose content. For the genes involved in pectin catabolism, many key genes were suppressed by the co-silencing of *SlRCAR9*, *SlRCAR11*, *SlRCAR12*, and *SlRCAR13*. In transgenic tomato fruit, the expression of *PG*, *TBG3*, and *TBG4* was strongly reduced during ripening, compared with WT; in particular, the *PG* gene was hardly expressed (Fig. 7c). The expression of several other key genes involved in pectin catabolism was increased in transgenic fruit compared with control, including *PGbeta*, *TPGL*, *PE*, *PE53*, *TBG5*, *TBG6*, and *TBG7* (Fig. 7c).

### Transgenic fruit silenced by *SlRCAR9*, *SlRCAR11*, *SlRCAR12*, and *SlRCAR13* presents shorter shelf life and reduced susceptibility to *B. cinerea*

**Figure 8:**
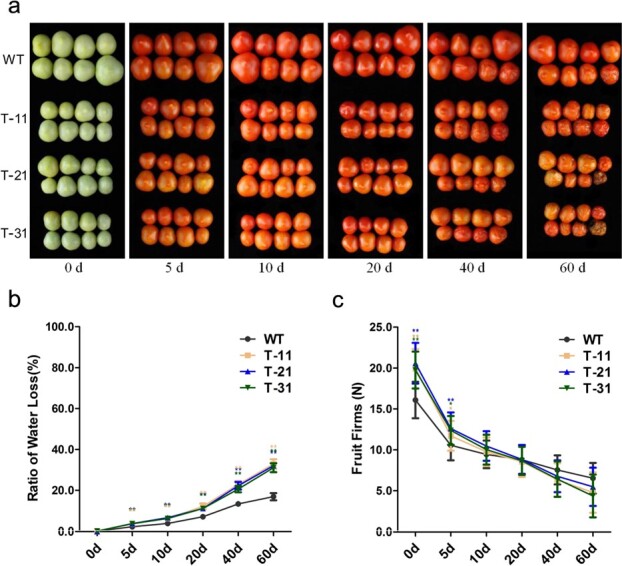
Co-silencing of SlRCAR9, SlRCAR11, SlRCAR12, and SlRCAR13 shortens fruit shelf-life. **a** Visual quality alteration of WT and transgenic (T-11, T-21, T-31) tomato fruit during post-harvest storage. **b** Physiological loss of water in WT and transgenic tomato during post-harvest storage. Values represent mean ± standard deviation (*n* = 20). **c** Fruit firmness alteration of WT and transgenic tomatoes during post-harvest storage. Values represent mean ± standard deviation (*n* = 30). ^**^P < .01 by *t*-test.

To determine the impact of ABA signaling block caused by the co-silencing of *SlRCAR9*, *SlRCAR11*, *SlRCAR12*, and *SlRCAR13* on shelf life, the fruits of transgenic and WT tomatoes were harvested at the breaker stage and stored at 25 ± 2°C with 85–90% relative humidity until deterioration was complete. In our research, the transgenic fruits were wrinkled after 22–25 days of storage, whereas WT fruits showed similar symptoms until 32–37 days of storage (Fig. 8a). Transgenic fruits exhibited higher physiological water loss than that of control at all storage stages (Fig. 8b). Meanwhile, faster softening was observed in transgenic fruits, and the firmness of transgenic fruits was <8 N after 24 ± 2 days of storage, whereas that of WT control did not drop below 8 N until 35 ± 3 days of storage (Fig. 8c).

**Figure 9:**
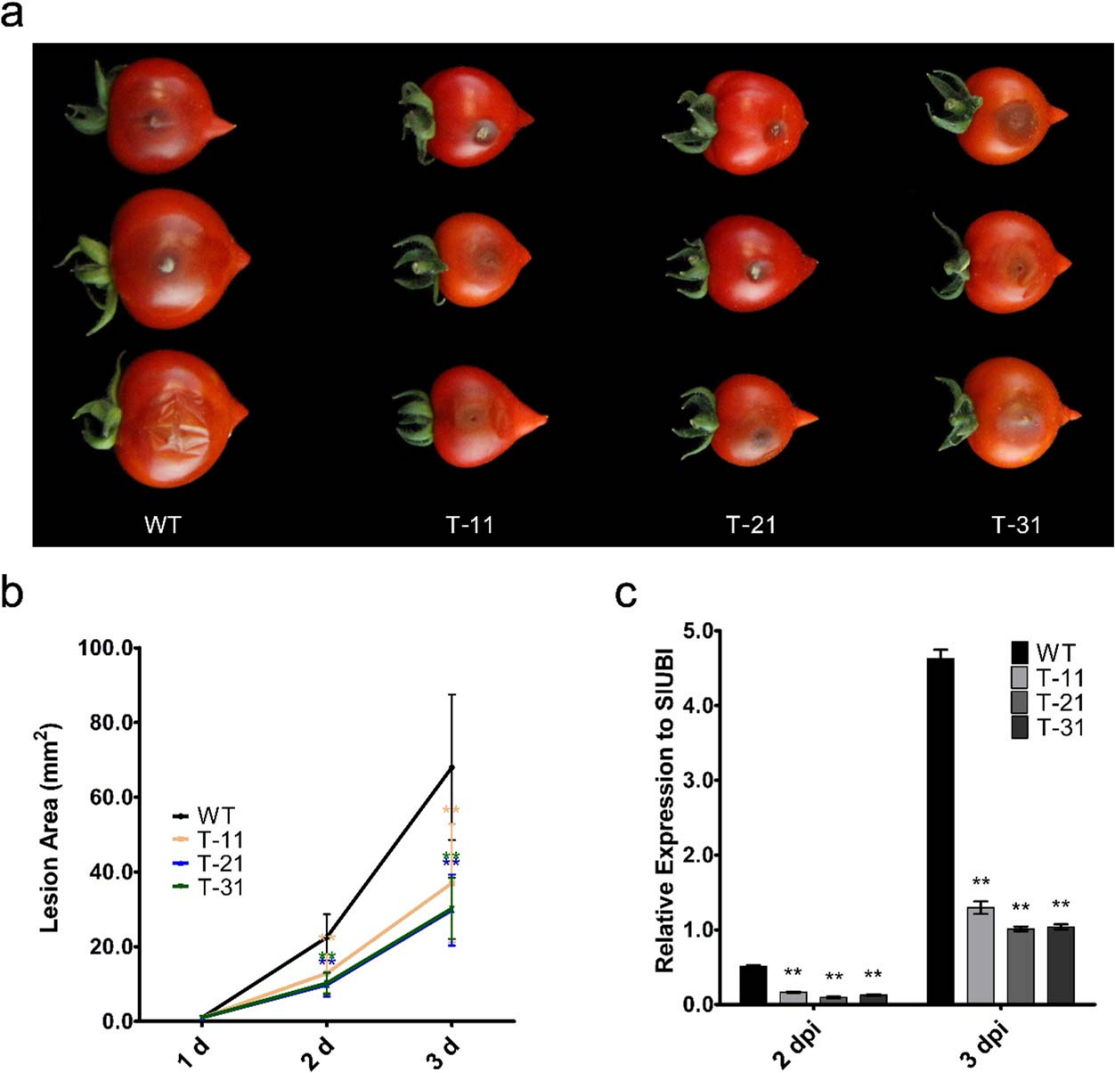
Four-gene co-silencing alters fruit pathogen susceptibility in tomato. **a** Symptoms of WT and transgenic (T-11, T-21, T-31) fruits infected with *B. cinerea* at 3 dpi. **b** Lesion area at 3 dpi. Error bars show mean ± SD (*n* = 30); ^**^P < .01 by *t*-test. **c***B. cinerea* relative growth on WT and transgenic fruits at 2 and 3 dpi, tested by qRT–PCR, using *BcCutA* as a test gene for *B. cinerea*. Data represent mean ± standard deviation.

*B. cinerea* (B05.10) is the causal agent of grey mold disease, which is an important post-harvest pathogen of tomato [36]. To explore the impact of ABA signaling block on the susceptibility of tomato fruits, a spore suspension of *B. cinerea* was used to infect fruits of transgenic and WT tomato. When intact tomato fruits were injected with *B. cinerea* spore suspension, more serious infection symptoms were observed on WT fruits compared with transgenic fruits at 3 days post-inoculation (dpi) (Fig. 9a). The lesion diameters on WT were nearly twice those on transgenic fruits, and lesion area was nearly three times that on transgenic fruits (Fig. 9b). qRT–PCR confirmed that more *B. cinerea* accumulated in WT than in transgenic fruits (Fig. 9c). Notably, our results indicated that ABA signaling block caused by the co-silencing of *SlRCAR9*, *SlRCAR11*, *SlRCAR12*, and *SlRCAR13* led to faster fruit senescence and shorter fruit shelf-life as well as reduced *B. cinerea* susceptibility in tomato.

## Discussion

### Abscisic acid receptors encoded by *SlRCAR9*, *SlRCAR12*, *SlRCAR11*, and *SlRCAR13* mediated ABA signaling associated with fruit ripening in tomato

Given the complexity of ABA signaling, precise regulation is particularly important for specific physiological processes, and regulation primarily depends on numerous specific ABA signaling components, such as specific ABA receptors (PYL/RCARs) [20–22, 37]. As the core components of ABA signaling, ABA receptors could trigger the downstream signaling cascade with A-group PP2C and SnRK2, so specific ABA receptors would play a crucial role in the precise regulation of fruit ripening [38, 39]. Undoubtedly, transduction weakening of the ABA signal associated with fruit development and ripening by suppressing ABA receptors should affect the process of fruit development and ripening in tomato [10, 13]. Nevertheless, the ABA receptors mediating the ABA signaling associated with fruit ripening have not been characterized in previous reports.

In this study, SlRCAR9, SlRCAR12, SlRCAR11, and SlRCAR13 have been identified as the crucial ABA receptors mediating ABA signal transduction for fruit ripening by multi-gene interference (RNAi) in tomato. The fruit ripening time was prolonged when these receptors were co-silenced. In addition, the results of single-gene silencing, double-gene silencing, and three-gene silencing of these four receptors showed that the fruit ripening time of transgenic tomatoes was significantly prolonged to varying degrees when the *SlRCAR11* and/or *SlRCAR13* gene was/were silenced (Fig. 2b; Supplementary Table S6). Moreover, the co-silencing of *SlRCAR11* and *SlRCAR13* could enhance the effect of fruit ripening time prolongation, compared with single silencing of these receptor genes (Fig. 2b; Supplementary Table S6). Notably, the fruit ripening time of single-gene-silenced, double-gene-silenced, and three-gene-silenced tomatoes was shorter than that of four-gene (*SlRCAR9*, *SlRCAR12*, *SlRCAR11*, and *SlRCAR13*) co-silenced transgenic tomatoes (Fig. 2). Gonzalez-Guzman *et al*. [24] proved that Solyc06g050500.2.1 (*SlRCAR 10*) and Solyc03g007310 (*SlRCAR 12*) mediated the ABA signal in response to drought stress in tomato by single-gene overexpression in *Arabidopsis*, and the drought resistance of transgenic *Arabidopsis* was improved, but the role of these genes in fruit ripening has not been reported. Thus, the results of this work suggested that all of these four ABA receptors played a role in fruit ripening, whereas *SlRCAR11* and *SlRCAR13* played more important roles than *SlRCAR9* and *SlRCAR12* during fruit ripening. Based on the results obtained in this study, when the receptor genes *SlRCAR9*, *SlRCAR12*, *SlRCAR11*, and *SlRCAR13* were silenced, the ABA signaling related to fruit ripening could be weakened, thereby affecting the physiological and biochemical processes of ripening, such as prolonged fruit ripening time, delayed pigmentation, and increased fruit firmness [10, 13].

### Abscisic acid signaling regulation of fruit ripening required synergistic action of ethylene signaling in tomato

Fruit ripening is a complex developmental process, which involves a series of coordinated changes in color, texture, flavor, and nutritional characteristics in fleshy fruit, and it is regulated by hormones, genes, and other factors [1, 2]. Given the complexity of the process, hormones cannot act alone in regulating fruit ripening, which requires the synergy of other hormones [3–5]. Ethylene was generally considered as the most crucial hormone for fruit ripening regulation of climacteric fruit, and ABA has been proven to be another important hormone in regulating climacteric fruit ripening, similar to ethylene [8–11]. Nevertheless, the clear regulatory relationship between the ABA signal and the ethylene signal remains unknown during fruit ripening in tomato. In *Arabidopsis*, the mutants *pyr1*/*pyl1*/*pyl4* and *pyr1*/*pyl1*/*pyl2*/*pyl4* were obtained through multi-mutation of ABA receptors, such as *AtPYR1*, *AtPYL1*, and *AtPYL4*, and the resulting co-function loss led to insensitivity to ABA during seed germination, root growth, and stomatal opening and closing [20]. However, to date the ABA-insensitive mutant for fruit ripening has not been described in climacteric fruit due to the lack of appropriate models. In this work, transgenic tomato SlRCAR9-12-11-13 showed evidence of ripening inhibition at the early stage of fruit ripening (Fig. 3), and an ABA-insensitive phenotype of fruit ripening was observed in the transgenic tomato, which might be due to the block of ABA signaling caused by the co-silencing of *SlRCAR9*, *SlRCAR11*, *SlRCAR12*, and *SlRCAR13* (Fig. 4). Therefore, SlRCAR9-12-11-13 showed great potential as an ideal model to investigate the connection between ABA signal and the ethylene signal during fruit ripening.

Although the transgenic tomato fruits of SlRCAR9-12-11-13 were insensitive to ABA during fruit ripening, they were sensitive to exogenous ethylene, and could be ripened effectively by ethylene treatment (Figs 3 and 4). Moreover, co-application of ABA and 1-MCP still significantly prolonged the fruit ripening time of transgenic tomato, in a similar manner to WT, and the effects were the same as that of 1-MCP treatment alone (Fig. 4). These results suggested that 1-MCP-mediated suppression of the ethylene signal led to ABA insensitivity with respect to fruit ripening in tomato. Based on these results, the block of ABA signaling did not affect the induction capability of ethylene in fruit ripening, whereas the suppression of the ethylene signal could result in ABA insensitivity during tomato fruit ripening. In previous reports, exogenous ABA could not restore the ripening process of the tomato fruit-ripening-block mutant *ripening-inhibitor* (*rin*), which further confirmed that ethylene signal block could lead to ABA insensitivity during fruit ripening in tomato (Supplementary Figure S5) [40]. These results suggested that the action of ABA signaling requires the synergies of ethylene signaling (or dependence on ethylene signaling) during fruit ripening, and the ABA signaling pathway might be located upstream of ethylene signaling in this process of tomato ripening (Fig. 4; Supplementary Figure S5).

**Figure 10:**
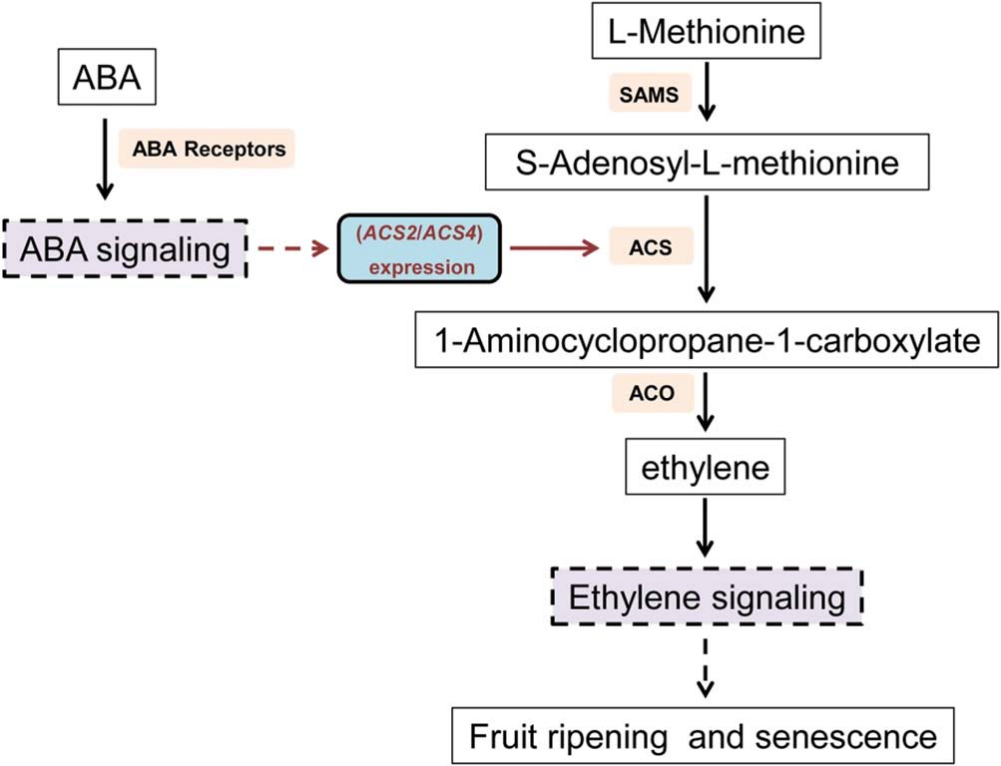
Proposed model of ABA–ethylene crosstalk during tomato fruit ripening. SAMS, *S*-adenosylmethionine synthetase; ACS, 1-aminocyclopropane-1-carboxylate synthase; ACO, aminocyclopropanecarboxylate oxidase.

Several previous reports revealed that exogenous ABA could promote tomato ripening by affecting crucial gene expression related to ethylene biosynthesis [10, 11, 19]. In this study, the block of ABA signaling related to fruit ripening severely inhibited the transcription of ethylene biosynthetic genes and other related regulators, such as *ACS2*, *ACS4*, *RIN*, *NOR*, and *E8*, and then significantly decreased ethylene production at the early stage of fruit ripening in tomato (Fig. 5). Our results, combined with previous reports, further confirmed that ABA signaling could regulate fruit ripening depending on ethylene signaling, and it would be located upstream of ethylene signaling during the early stage of tomato fruit ripening (the deduced working model of ABA–ethylene crosstalk is presented in Fig. 10). Moreover, ABA could trigger ethylene production and promote the ripening of other fresh fruits, such as banana, strawberry, and mango [16–18]. Therefore, our inference of interaction between ethylene signaling and ABA signaling during fruit ripening could be applied to other climacteric fruits. Based on the deduced relationship between ABA signaling and ethylene signaling, it can be readily understood that ABA signaling block can lead to a series of changes in physiological indicators of fruit ripening in our experiments, such as ripening time prolongation and fruit firmness increase.

### Abscisic acid signaling affected cell wall metabolism and fruit susceptibility in fruit ripening

With the assistance of ethylene, ABA participates in the regulation of fruit ripening, such as fruit softening. In previous studies, the suppression of ABA biosynthesis by *SlNCED1*-silencing resulted in a similar expression inhibitory effect on genes related to cell wall metabolism [10]. In this work, the ABA signaling block produced by co-silencing of the ABA receptors associated with fruit ripening induced down-regulation of the key genes related to cell wall metabolism, including *Cel2*, *Exp1*, *XET2-F1*, *XETB1-F2*, *XETB2-F2*, *XTH3-F1*, *XTH9-F1*, *PG*, *TBG3*, and *TBG4* (Fig. 7) [10, 41–46], which would weaken the cell wall metabolism of the tomato. Thus, the significant increase in cellulose and decrease in WSP could result in cell wall thickening, and subsequently lead to the increase in fruit firmness in transgenic tomato (SlRCAR9-12-11-13) (Figs 6 and 7). Normally, the increase in fruit firmness could prolong shelf life, which would be beneficial for fruit post-harvest storage [10, 32]. However, a shorter shelf life was observed in the transgenic tomato SlRCAR9-12-11-13, probably because of the rapid loss of water and excessive softening of the fruit at the later stage of long-term storage (Fig. 8). Therefore, the blocking of ABA signaling caused by co-silencing of *SlRCAR9*, *SlRCAR11*, *SlRCAR12*, and *SlRCAR13* genes could make the fruit more intolerant of storage.

Ethylene is a crucial factor for cell wall metabolism and should not be ignored during tomato fruit ripening. In reviews by Wang *et al.* [46] and Bennett *et al.* [47], the expression of a variety of cell wall-modifying protein genes was closely related to endogenous ethylene synthesis and perception. Among these genes, pectin metabolism genes, such as pectinmethylesterase (*PME*) and polygalacturonase (*PG*), may contribute to overall fruit softening depending on ethylene regulation [46, 47]. Moreover, the *TBG4* gene was dramatically repressed in ripening-impaired mutant *rin* and *nor* tomato fruits, and was defective in triggering ripening-related ethylene biosynthesis [40, 46, 47]. In strawberry, the application of ethephon and 1-MCP could significantly alter the expression of *FaPME1*, *FaPG1*, and other genes related to cell wall metabolism, thereby affecting the contents of cellulose and pectin of the fruits [48]. Therefore, it is easy to understand the rapid senescence rate and short shelf life of transgenic tomato SlRCAR9-12-11-13 (Fig. 8). Here, the sharp increase in ethylene production led to rapid fruit softening in transgenic tomatoes at the later stage of fruit ripening (Fig. 5a), which resulted in rapid fruit senescence. Based on previous reports [49], the smaller fruits of transgenic tomato have a greater proportional weight loss, because of the large specific surface area ratio, which could further aggravate the storage intolerance in tomato (Fig. 3e). However, the rapid senescence rate did not affect the low susceptibility of transgenic tomato to *B. cinerea*, probably due to the thick cell wall of transgenic fruits (Fig. 9) [32, 50]. Nevertheless, at the later stage of fruit ripening and senescence in tomato, the reasons for the sharp increase in ethylene production caused by the blocking of ABA signaling related to fruit ripening remain unknown and need further investigation.

In conclusion, the results of this work suggested that the ABA receptor genes *SlRCAR9*, *SlRCAR12*, *SlRCAR11*, and *SlRCAR13* could mediate ABA signaling to regulate fruit ripening at the early stage in tomato, and *SlRCAR11* and *SlRCAR13* played comparatively more important roles. Moreover, the action of ABA signaling mediated by these ABA receptor genes in fruit ripening depended on ethylene signaling, and ABA signaling should be located upstream of ethylene signaling at the early stage of tomato fruit ripening. Our findings provide more evidence of the complex regulatory network of fruit ripening in tomato.

## Materials and methods

### Plant materials and growth conditions

Tomato (*Solanum lycopersicum* cv. ‘Micro-Tom’) plants were grown in a greenhouse under the following conditions: 25°C for 14 hours in light at 250 μmol m^−2^ s^−1^ light intensity and 20°C for 10 hours in darkness, with 80% humidity. Tissues, including root, stem, leaf, flower, immature green fruit (IM), mature green fruit (MG), breaker stage fruit (Br), orange stage fruit (Br + 3), and red ripe fruit (Br + 7), were collected from wild-type (WT) plants for analysis of gene expression patterns. Leaf samples were collected from WT and transgenic tomato plants to determine silencing efficiency. All samples were immediately frozen using liquid nitrogen and stored at −80°C before use.

### RNA isolation and quantitative reverse transcription–PCR analysis

Total RNA was extracted and purified using the Qiagen (Germany) RNeasy Plant Mini Kit according to the manufacturer’s instructions. The primers used for quantitative reverse transcription (qRT)–PCR were designed by an online website at www.genscript.com/ssl-bin/app/primer. qRT–PCR was performed using Fast SYBR Mixture (CWBIO, China) through a two-step method. Three biological replicates were performed for all qRT–PCR experiments. The relative expression level was calculated on the basis of the 2^−ΔΔCt^ method, and *SlUBI* (Solyc07g064130) was used as an internal reference gene.

### Characterization of the tomato PYR/PYL/RCAR family

Following Gonzalez-Guzman *et al*. [24], information on 15 ABA receptor candidate genes was obtained from the SGN database (http://solgenomics.net/), and the genes were numbered from *SlRCAR1* to *SlRCAR15* (Supplementary Table S1). Multiple-sequence alignments of RCARs from different species were analyzed by ClustalX 1.81. The phylogenetic tree was constructed by MEGA6. Expression patterns of 15 tomato *RCAR* genes were predicted by Tom-Express (http://gbf.toulouse.inra.fr/tomexpress)and their expression was verified by qRT–PCR using vegetative and reproductive tissues.

### Vector construction for multiple-gene silencing

The pCAMBIA vectors have been widely used for *Agrobacterium*-mediated genetic transformation for a range of plant species [25–27]. In this study, pCAMBIA1301 was constructed into a multiple-gene RNAi vector system, integrated with pCAMBIA1301m and pCAMBIA1301s, to perform multiple-gene silencing of *SlRCAR*s (the detailed protocols of the multiple-gene RNAi vector system are listed in Supplementary Data Appendixes 1 and 2).

DNA fragments (~200–500 bp in length) from non-conserved regions of 15 *SlRCAR* genes were selected to construct the RNAi vector (the primer sequences are listed in Supplementary Table S3). The 15 *SlRCAR* candidates were divided into four groups based on phylogenetic analysis and expression pattern analysis and constructed into multiple-gene silencing vectors. For four-gene silencing vector construction, each DNA fragment of two genes was spliced into a nucleic acid sequence fusion fragment by overlapping PCR. In addition, the sense orientation fusion fragment contained the restriction sites SacI and SalI, and the antisense fragment contained the restriction sites SmaI and XbaI. One fusion fragment was inserted into pCAMIBA1301m and driven by a *Cauliflower mosaic virus 35S* promoter, and the other fusion fragment was inserted into pCAMIBA1301s. Then, the complete expression element, containing the *35S* promoter, the loop structure of the fusion fragment, and the *NOS* terminator, was spliced from pCAMIBA1301s into pCAMIBA1301m based on the restriction sites SpelI and SwaI. Then, two full expression systems were obtained for the vector pCAMIBA1301m, which can silence four genes simultaneously. The protocols for the construction of a three-gene and two-gene silencing vector were similar to that for four-gene silencing vector construction.

### Plant transformation

The RNAi plasmids for multiple-gene silencing were transferred into *Agrobacterium tumefaciens* strain GV3101 and used for genetic transformation in tomato, as described by Fillatti *et al*. [28]. Then, PCR detection was performed to verify the presence of T-DNA insertion into the genomic DNA of transgenic plants and screen the positive lines. The primer sequences are listed in Supplementary Table S4. Using qRT–PCR testing, transgenic plants with >60% repression efficiency were considered to be effectively silenced plants. The obtained homozygous RNAi lines of the *T*_2_ generation were used for the following experiments.

### Analysis of fruit ripening time and other phenotypes

The ripening time of fruit was determined as the number of days from anthesis to the breaker stage. For fruit ripening time analysis, the first five fruits on the rachis of the first inflorescence in each plant were collected. All lines of individual multiple/single-silence vectors were counted, and 20 plants were selected for the *T*_2_ and *T*_3_ generations using WT tomato as control. Student’s *t*-test was used to analyze the significance of differences between transgenic fruits and WT fruits, and *P* < .05 was considered statistically significant. In addition, fruits of SlRCAR9-12-11-13 transgenic plants were used to analyze their seed number, fruit weight, fruit set, and plant yield in the T2 and T3 generations, using the WT as control. All statistical samples were >30 (*n* > 30), and values are shown as means ± standard deviation.

### Analysis of fruit texture

Fruit firmness was determined using a GY-4 digital fruit sclerometer (Aiwoshi, China), according to Fan *et al*. [29] and Zou *et al*. [30]. The samples were collected at different fruit ripening stages in the *T*_2_ generation and WT, including the Br, Br + 3, and Br + 7 stages. The fruit number of each stage was set to 30, and values are presented as mean ± standard deviation.

### Measurement of ethylene production and abscisic acid content

Three intact fruits were placed into a 100-ml gas collecting bottle, capped with a rubber stopper for 1 hour, to test ethylene production according to the procedure used by Zhang *et al*. [31] and Zou *et al*. [30], with 10 replicates for each trial. For the ABA quantification test, fresh tomato fruits were collected at the MG, Br, and Br + 3 stages, and their ABA contents were tested by HPLC using an Agilent 1200-LC system, referring to the method used by Zaharah *et al*. [17]. Ten fruits were used for each ABA test, and three biological replicates were conducted. All values were represented as mean ± standard deviation.

### Hormone and hormone inhibitor treatment

The fruits of SlRCAR9-12-11-13 in the *T*_3_ generation were harvested at 33 days post-anthesis (DPA) (MG) and randomly sorted into seven groups. The first group was soaked in double distilled-water for 15 min as the control treatment and then placed in a plastic crisper at 25 ± 2°C and 85–90% relative humidity. The other four groups were incubated in a solution/solution mixture of ethephon (100 mg l^−1^), ABA (100 mg l^−1^), nordihydroguaiaretic acid (NDGA) (100 mg l^−1^), and ethephon (100 mg l^−1^) + NDGA (100 mg l^−1^) for 15 min, and stored under the same conditions. The sixth group was treated with 1-methylcyclopropene (1-MCP) (0.5 μl l^−1^) at 25°C for 24 hours and then stored under the same conditions. The seventh group was immersed in the ABA solution for 15 min, then treated with 1-MCP, and stored under the same conditions. Fruits from WT plants were collected and processed in a similar manner. Subsequently, their fruit ripening time was recorded. The fruit number of each group was set to 30 (*n* = 30), and values are presented as mean ± standard deviation. Student’s *t*-test was used to analyze differences, and *P* < .05 was considered statistically significant.

### Histological analysis of fruit pericarp

Analysis of fruit epidermis and pericarp tissue cell was performed in accordance with the methods described by Yang *et al*. [32]. Ten epidermis slices from the identical position of different fruits were isolated at the Br, Br + 3, and Br + 7 (red) stages. The inner surface of the epidermis was observed with a microscope (Nikon 80i, Japan). The epidermal cell size, cell separation, and cell number were measured by using Image-Pro Plus software at three different positions. For cytological assessment of pericarp cells, three samples were harvested at the MG, Br, Br + 3, and Br + 7 (red) stages, and their pericarp tissue was collected for paraffin sectioning. Cuticle thickness, cell size, cell separation, and cell number were estimated using Image-Pro Plus software. All results were expressed as mean ± standard deviation of three biological replicates.

### Analysis of fruit cell wall components

The cell wall components, including pectin, cellulose, and hemicellulose, were extracted and analyzed in accordance with the method described by Yang *et al*. [32]. The fruits were harvested from WT and transgenic (three lines of SlRCAR9-12-11-13 in the *T*_3_ generation) plants at the breaker stage, and their pericarps were collected for cell wall component analysis. The cellulose content was measured by anthrone–sulfuric acid colorimetry; the hemicellulose content was tested using the lichen phenol–HCl method; water-soluble pectin (WSP) and protopectin were analyzed in accordance with the *m*-hydroxydiphenyl method, and total pectin was calculated as WSP plus protopectin. For each sample, the tissue was pooled from 20 individual fruits. Three independent experiments were performed, and the results were expressed as mean ± standard deviation.

### Analysis of fruit shelf life

To determine the shelf life of tomato fruit, fruits at the breaker stage were surface-sterilized, placed in a glass jar, and stored at 25 ± 2°C and 85–90% relative humidity. For an interval of 5 days, physiological water loss, visual softening, and fruit wrinkling were assessed [33]. The fruit number of each group was set to 30 (*n* = 30), and values are presented as mean ± standard deviation.

### *Botrytis cinerea* infection

*Botrytis cinerea* (B05.10) was grown on potato dextrose agar medium, and the conidia were collected and filtered as described by Stefanato *et al*. [34]. Intact fruits from WT and transgenic lines (SlRCAR9-12-11-13) at the red ripe stage (Br + 7) were surface-sterilized and infected with *B. cinerea* spore suspension by wound inoculation (1 mm diameter and 2 mm depth). A total of 30 fruits were used for each group as experimental samples. The fungal culture was diluted with sterile double-distilled water to 1 × 10^5^ spores/ml, and then used to infect the fruits. Then, the infected fruits were stored at 25°C to stimulate germination. Lesion diameter was measured after 24, 48, and 72 hours to calculate the lesion area. An area of lesion tissue of 1 cm^2^ was collected after treatment for 48 and 72 hours to test the growth of *B. cinerea*, using the test gene *BcCutA* (Z69264.1) gene by qRT–PCR, according to the method described by Zhang *et al*. [35]. The *SlUBI* (Solyc01g056940) was used as tomato internal reference gene. The relative growth of *B. cinerea* was obtained as the ratio of *B. cinerea* DNA to tomato DNA based on the 2^−ΔΔCt^ method.

## Acknowledgements

We thank Dr Menghuan Li of Chongqing University for critical reading and revising the English language of this article. This study was supported by the National Key Research and Development Program (2016YFD0400101), the National Natural Science Foundation of China (31772370 and 31972470), the Fundamental Research Funds for the Central Universities (2021CDJZYJH-002), and the Project of Chongqing Science and Technology Commission (cstc2019jscx-dxwtBX0027).

## Author contributions

J.Z. and Z.L. designed the research; J.Z., N.L., N.H., N.T., H.C., Y.L., J.C., W.J., and Y.G. performed the experiments; J.Z. analyzed the data and wrote the paper; Y.L. provided English language editing; and J.Z., J.Y. and Z.L. revised the paper.

## Data availability

All relevant data are presented within the paper and its supplementary files.

## Conflict of interest

The authors declare that they have no conflict of interest.

## Supplementary data

Supplementary data is available at *Horticulture Research* online.

## Supplementary Material

Web_Material_uhac057
