# Correction to: Co-silencing of ABA receptors (SlRCAR) reveals interactions between ABA and ethylene signaling during tomato fruit ripening

**DOI:** 10.1093/hr/uhac209

**Published:** 2022-09-29

Correction to: *Horticulture Research*https://doi.org/10.1093/hr/uhac057 published online 05 June 2022

 In the originally published manuscript there were errors in the labelling of three figures. In Figure 3b, the developing time of fruits was erroneously omitted. In Figure 4e, the group names ``Ethephon+NDGA'' and ``ABA+1-MCP'' were the wrong way round. In the Supplementary data, in Figure S3b there are two histograms labelled ``SIRCAR9-11-12''. The histogram on the right should read ``SIRCAR9-11-13''. These errors have been corrected online.

