# Supplementary material for: Co-silencing of ABA receptors (SlRCAR) reveals interactions between ABA and ethylene signaling during tomato fruit ripening
Source: Hortic Res. 2022 Jun 5;9:uhac057. doi: 10.1093/hr/uhac057 (PMC9171117; doi:10.1093/hr/uhac057)
Supplement: Web_Material_uhac057 [file web_material_uhac057.zip › Supplementary appendix 1, appendix 2, and appendix 3.docx]

**Supplementary appendix 1, appendix2, and appendix3**

**Supplementary appendix 1**

**Detailed protocols of multiple-gene RNAi vector system**

The pCAMBIA vectors have been widely used for *Agrobacterium*-mediated genetic transformation of a range of plant species (Curtis and Grossniklaus 2003; Lee and Gelvin 2008). In this study, the vector pCAMBIA1301 was reformed into a multiple-gene RNAi vector system pCAMBIA1301m and pCAMBIA1301s to perform multiple-gene silencing of *RCARs*. An expression cassette, namely, RNAi-E- cassette, was designed, containing a CaMV 35S promoter (from pCAMBIA1301), PDK intron (from pHANNIBAL), and NOS terminator (from pCAMBIA1301). Three multiple clone sites (MCSs) were designed in this expression cassette. MCS1 was located between CaMV 35S promoter and PDK intron, containing four restriction sites, including *Hind* III, *Xba* I, *Sal* I, and *Pst* I. MCS2 was located between PDK intron and NOS terminator, containing four restriction sites, including *Kpn* I, *Xma* I, *Eco53k* I, *Sma* I, and *Sac* I. MCS3 was located on the upstream of CaMV 35S promoter 5′ end, possessing six restriction sites, including *spe*I, *Hpa*I, *stu*I, *Ecor*I, *Apa*I, and *Asc*I. Subsequently, a restriction site *Swa* I was added into the position of downstream of MCS3 3′ end, and the restriction site BamH I was added into the downstream of NOS terminator 3′end. Thus the RNAi-E-cassette was reformed into the RNAi-E- cassette-E; the restriction site *Swa* I, *Pme* I and *BamH* I were added into the position of downstream of NOS terminator 3′, to reform the RNAi-E-cassette into the RNAi-E-cassette-C. Afterwards, a 30bp sequence 5′ ACACAGGAAACAGCTATGACCATGATTACG 3′ homologous to 1301 was added into the upstream 5'end of RNAi-E-cassette-E and RNAi-E-cassette-C, and the other 30bp sequence 5′ AGCTTGGCACTGGCCGTCGTTTTACAACGT3′ was add into the downstream 3' end of RNAi-E-cassette-E and RNAi-E-cassette-C. Then, they were reformed into RNAi-E-cassette-E2 and RNAi-E-cassette-C2. Here, pCAMBIA1301 was used to construct the multiple-gene RNAi vector system. Considering that a hidden restriction site *Sma* I on the pCAMBIA1301 vector could affect the use of the expression cassette, a nucleic acid fragment from 9941 to 10800 on the pCAMBIA1301 vector was artificially synthesized to change the base G at position 9985 of pCAMBIA 1301 to C and remove the *Sma* I restriction site, and named as *Fragment-Delete-Sma I*. Subsequently, restriction enzymes Aat II and Bst XI were used to delete the fragment from 9944 to 10785 on the pCAMBIA1301 vector and replaced by *Fragment-Delete-Sma I* with the same double digestion sites. The vector was named as *pCAMBIA 1301-Delete-Sma I*. Next, the MCS of *pCAMBIA 1301-Delete-Sma I* was removed using restriction enzymes *EcoR*I and *Hind*III. RNAi-E-cassette-E2 was constructed into *pCAMBIA 1301-Delete-Sma I* by homologous recombination to reform pCAMBIA1301m, and RNAi-E-cassette-C2 was constructed into *pCAMBIA 1301-Delete-Sma I* to reform pCAMBIA1301s. The multiple-gene RNAi vector system pCAMBIA1301m and pCAMBIA1301s were used to perform multiple-gene silencing of *RCARs*. The map and sequence of pCAMBIA1301m and pCAMBIA1301s are listed in detail in Supplementary appendix 2. The vector maps of pCAMBIA1301m and pCAMBIA1301s were drawn based on the vector sequence using Snapgene 4.2.4.

**Supplementary appendix 2**

**Sequence of pcambia1301m and pcambia1301s**

**2.1 Sequence of pcambia1301m**

ATCTGAGGGTAAATTTCTAGTTTTTCTCCTTCATTTTCTTGGTTAGGACCCTTTTCTCTTTTTATTTTTTTGAGCTTTGATCTTTCTTTAAACTGATCTATTTTTTAATTGATTGGTTATGGTGTAAATATTACATAGCTTTAACTGATAATCTGATTACTTTATTTCGTGTGTCTATGATGATGATGATAGTTACAGAACCGACGACTCGTCCGTCCTGTAGAAACCCCAACCCGTGAAATCAAAAAACTCGACGGCCTGTGGGCATTCAGTCTGGATCGCGAAAACTGTGGAATTGATCAGCGTTGGTGGGAAAGCGCGTTACAAGAAAGCCGGGCAATTGCTGTGCCAGGCAGTTTTAACGATCAGTTCGCCGATGCAGATATTCGTAATTATGCGGGCAACGTCTGGTATCAGCGCGAAGTCTTTATACCGAAAGGTTGGGCAGGCCAGCGTATCGTGCTGCGTTTCGATGCGGTCACTCATTACGGCAAAGTGTGGGTCAATAATCAGGAAGTGATGGAGCATCAGGGCGGCTATACGCCATTTGAAGCCGATGTCACGCCGTATGTTATTGCCGGGAAAAGTGTACGTATCACCGTTTGTGTGAACAACGAACTGAACTGGCAGACTATCCCGCCGGGAATGGTGATTACCGACGAAAACGGCAAGAAAAAGCAGTCTTACTTCCATGATTTCTTTAACTATGCCGGAATCCATCGCAGCGTAATGCTCTACACCACGCCGAACACCTGGGTGGACGATATCACCGTGGTGACGCATGTCGCGCAAGACTGTAACCACGCGTCTGTTGACTGGCAGGTGGTGGCCAATGGTGATGTCAGCGTTGAACTGCGTGATGCGGATCAACAGGTGGTTGCAACTGGACAAGGCACTAGCGGGACTTTGCAAGTGGTGAATCCGCACCTCTGGCAACCGGGTGAAGGTTATCTCTATGAACTCGAAGTCACAGCCAAAAGCCAGACAGAGTCTGATATCTACCCGCTTCGCGTCGGCATCCGGTCAGTGGCAGTGAAGGGCCAACAGTTCCTGATTAACCACAAACCGTTCTACTTTACTGGCTTTGGTCGTCATGAAGATGCGGACTTACGTGGCAAAGGATTCGATAACGTGCTGATGGTGCACGACCACGCATTAATGGACTGGATTGGGGCCAACTCCTACCGTACCTCGCATTACCCTTACGCTGAAGAGATGCTCGACTGGGCAGATGAACATGGCATCGTGGTGATTGATGAAACTGCTGCTGTCGGCTTTCAGCTGTCTTTAGGCATTGGTTTCGAAGCGGGCAACAAGCCGAAAGAACTGTACAGCGAAGAGGCAGTCAACGGGGAAACTCAGCAAGCGCACTTACAGGCGATTAAAGAGCTGATAGCGCGTGACAAAAACCACCCAAGCGTGGTGATGTGGAGTATTGCCAACGAACCGGATACCCGTCCGCAAGGTGCACGGGAATATTTCGCGCCACTGGCGGAAGCAACGCGTAAACTCGACCCGACGCGTCCGATCACCTGCGTCAATGTAATGTTCTGCGACGCTCACACCGATACCATCAGCGATCTCTTTGATGTGCTGTGCCTGAACCGTTATTACGGATGGTATGTCCAAAGCGGCGATTTGGAAACGGCAGAGAAGGTACTGGAAAAAGAACTTCTGGCCTGGCAGGAGAAACTGCATCAGCCGATTATCATCACCGAATACGGCGTGGATACGTTAGCCGGGCTGCACTCAATGTACACCGACATGTGGAGTGAAGAGTATCAGTGTGCATGGCTGGATATGTATCACCGCGTCTTTGATCGCGTCAGCGCCGTCGTCGGTGAACAGGTATGGAATTTCGCCGATTTTGCGACCTCGCAAGGCATATTGCGCGTTGGCGGTAACAAGAAAGGGATCTTCACTCGCGACCGCAAACCGAAGTCGGCGGCTTTTCTGCTGCAAAAACGCTGGACTGGCATGAACTTCGGTGAAAAACCGCAGCAGGGAGGCAAACAAGCTAGCCACCACCACCACCACCACGTGTGAATTACAGGTGACCAGCTCGAATTTCCCCGATCGTTCAAACATTTGGCAATAAAGTTTCTTAAGATTGAATCCTGTTGCCGGTCTTGCGATGATTATCATATAATTTCTGTTGAATTACGTTAAGCATGTAATAATTAACATGTAATGCATGACGTTATTTATGAGATGGGTTTTTATGATTAGAGTCCCGCAATTATACATTTAATACGCGATAGAAAACAAAATATAGCGCGCAAACTAGGATAAATTATCGCGCGCGGTGTCATCTATGTTACTAGATCGGGAATTAAACTATCAGTGTTTGACAGGATATATTGGCGGGTAAACCTAAGAGAAAAGAGCGTTTATTAGAATAACGGATATTTAAAAGGGCGTGAAAAGGTTTATCCGTTCGTCCATTTGTATGTGCATGCCAACCACAGGGTTCCCCTCGGGATCAAAGTACTTTGATCCAACCCCTCCGCTGCTATAGTGCAGTCGGCTTCTGACGTTCAGTGCAGCCGTCTTCTGAAAACGACATGTCGCACAAGTCCTAAGTTACGCGACAGGCTGCCGCCCTGCCCTTTTCCTGGCGTTTTCTTGTCGCGTGTTTTAGTCGCATAAAGTAGAATACTTGCGACTAGAACCGGAGACATTACGCCATGAACAAGAGCGCCGCCGCTGGCCTGCTGGGCTATGCCCGCGTCAGCACCGACGACCAGGACTTGACCAACCAACGGGCCGAACTGCACGCGGCCGGCTGCACCAAGCTGTTTTCCGAGAAGATCACCGGCACCAGGCGCGACCGCCCGGAGCTGGCCAGGATGCTTGACCACCTACGCCCTGGCGACGTTGTGACAGTGACCAGGCTAGACCGCCTGGCCCGCAGCACCCGCGACCTACTGGACATTGCCGAGCGCATCCAGGAGGCCGGCGCGGGCCTGCGTAGCCTGGCAGAGCCGTGGGCCGACACCACCACGCCGGCCGGCCGCATGGTGTTGACCGTGTTCGCCGGCATTGCCGAGTTCGAGCGTTCCCTAATCATCGACCGCACCCGGAGCGGGCGCGAGGCCGCCAAGGCCCGAGGCGTGAAGTTTGGCCCCCGCCCTACCCTCACCCCGGCACAGATCGCGCACGCCCGCGAGCTGATCGACCAGGAAGGCCGCACCGTGAAAGAGGCGGCTGCACTGCTTGGCGTGCATCGCTCGACCCTGTACCGCGCACTTGAGCGCAGCGAGGAAGTGACGCCCACCGAGGCCAGGCGGCGCGGTGCCTTCCGTGAGGACGCATTGACCGAGGCCGACGCCCTGGCGGCCGCCGAGAATGAACGCCAAGAGGAACAAGCATGAAACCGCACCAGGACGGCCAGGACGAACCGTTTTTCATTACCGAAGAGATCGAGGCGGAGATGATCGCGGCCGGGTACGTGTTCGAGCCGCCCGCGCACGTCTCAACCGTGCGGCTGCATGAAATCCTGGCCGGTTTGTCTGATGCCAAGCTGGCGGCCTGGCCGGCCAGCTTGGCCGCTGAAGAAACCGAGCGCCGCCGTCTAAAAAGGTGATGTGTATTTGAGTAAAACAGCTTGCGTCATGCGGTCGCTGCGTATATGATGCGATGAGTAAATAAACAAATACGCAAGGGGAACGCATGAAGGTTATCGCTGTACTTAACCAGAAAGGCGGGTCAGGCAAGACGACCATCGCAACCCATCTAGCCCGCGCCCTGCAACTCGCCGGGGCCGATGTTCTGTTAGTCGATTCCGATCCCCAGGGCAGTGCCCGCGATTGGGCGGCCGTGCGGGAAGATCAACCGCTAACCGTTGTCGGCATCGACCGCCCGACGATTGACCGCGACGTGAAGGCCATCGGCCGGCGCGACTTCGTAGTGATCGACGGAGCGCCCCAGGCGGCGGACTTGGCTGTGTCCGCGATCAAGGCAGCCGACTTCGTGCTGATTCCGGTGCAGCCAAGCCCTTACGACATATGGGCCACCGCCGACCTGGTGGAGCTGGTTAAGCAGCGCATTGAGGTCACGGATGGAAGGCTACAAGCGGCCTTTGTCGTGTCGCGGGCGATCAAAGGCACGCGCATCGGCGGTGAGGTTGCCGAGGCGCTGGCCGGGTACGAGCTGCCCATTCTTGAGTCCCGTATCACGCAGCGCGTGAGCTACCCAGGCACTGCCGCCGCCGGCACAACCGTTCTTGAATCAGAACCCGAGGGCGACGCTGCCCGCGAGGTCCAGGCGCTGGCCGCTGAAATTAAATCAAAACTCATTTGAGTTAATGAGGTAAAGAGAAAATGAGCAAAAGCACAAACACGCTAAGTGCCGGCCGTCCGAGCGCACGCAGCAGCAAGGCTGCAACGTTGGCCAGCCTGGCAGACACGCCAGCCATGAAGCGGGTCAACTTTCAGTTGCCGGCGGAGGATCACACCAAGCTGAAGATGTACGCGGTACGCCAAGGCAAGACCATTACCGAGCTGCTATCTGAATACATCGCGCAGCTACCAGAGTAAATGAGCAAATGAATAAATGAGTAGATGAATTTTAGCGGCTAAAGGAGGCGGCATGGAAAATCAAGAACAACCAGGCACCGACGCCGTGGAATGCCCCATGTGTGGAGGAACGGGCGGTTGGCCAGGCGTAAGCGGCTGGGTTGTCTGCCGGCCCTGCAATGGCACTGGAACCCCCAAGCCCGAGGAATCGGCGTGACGGTCGCAAACCATCCGGCCCGGTACAAATCGGCGCGGCGCTGGGTGATGACCTGGTGGAGAAGTTGAAGGCCGCGCAGGCCGCCCAGCGGCAACGCATCGAGGCAGAAGCACGCCCCGGTGAATCGTGGCAAGCGGCCGCTGATCGAATCCGCAAAGAATCCCGGCAACCGCCGGCAGCCGGTGCGCCGTCGATTAGGAAGCCGCCCAAGGGCGACGAGCAACCAGATTTTTTCGTTCCGATGCTCTATGACGTGGGCACCCGCGATAGTCGCAGCATCATGGACGTGGCCGTTTTCCGTCTGTCGAAGCGTGACCGACGAGCTGGCGAGGTGATCCGCTACGAGCTTCCAGACGGGCACGTAGAGGTTTCCGCAGGGCCGGCCGGCATGGCCAGTGTGTGGGATTACGACCTGGTACTGATGGCGGTTTCCCATCTAACCGAATCCATGAACCGATACCGGGAAGGGAAGGGAGACAAGCCCGGCCGCGTGTTCCGTCCACACGTTGCGGACGTACTCAAGTTCTGCCGGCGAGCCGATGGCGGAAAGCAGAAAGACGACCTGGTAGAAACCTGCATTCGGTTAAACACCACGCACGTTGCCATGCAGCGTACGAAGAAGGCCAAGAACGGCCGCCTGGTGACGGTATCCGAGGGTGAAGCCTTGATTAGCCGCTACAAGATCGTAAAGAGCGAAACCGGGCGGCCGGAGTACATCGAGATCGAGCTAGCTGATTGGATGTACCGCGAGATCACAGAAGGCAAGAACCCGGACGTGCTGACGGTTCACCCCGATTACTTTTTGATCGATCCCGGCATCGGCCGTTTTCTCTACCGCCTGGCACGCCGCGCCGCAGGCAAGGCAGAAGCCAGATGGTTGTTCAAGACGATCTACGAACGCAGTGGCAGCGCCGGAGAGTTCAAGAAGTTCTGTTTCACCGTGCGCAAGCTGATCGGGTCAAATGACCTGCCGGAGTACGATTTGAAGGAGGAGGCGGGGCAGGCTGGCCCGATCCTAGTCATGCGCTACCGCAACCTGATCGAGGGCGAAGCATCCGCCGGTTCCTAATGTACGGAGCAGATGCTAGGGCAAATTGCCCTAGCAGGGGAAAAAGGTCGAAAAGGTCTCTTTCCTGTGGATAGCACGTACATTGGGAACCCAAAGCCGTACATTGGGAACCGGAACCCGTACATTGGGAACCCAAAGCCGTACATTGGGAACCGGTCACACATGTAAGTGACTGATATAAAAGAGAAAAAAGGCGATTTTTCCGCCTAAAACTCTTTAAAACTTATTAAAACTCTTAAAACCCGCCTGGCCTGTGCATAACTGTCTGGCCAGCGCACAGCCGAAGAGCTGCAAAAAGCGCCTACCCTTCGGTCGCTGCGCTCCCTACGCCCCGCCGCTTCGCGTCGGCCTATCGCGGCCGCTGGCCGCTCAAAAATGGCTGGCCTACGGCCAGGCAATCTACCAGGGCGCGGACAAGCCGCGCCGTCGCCACTCGACCGCCGGCGCCCACATCAAGGCACCCTGCCTCGCGCGTTTCGGTGATGACGGTGAAAACCTCTGACACATGCAGCTCCCGGAGACGGTCACAGCTTGTCTGTAAGCGGATGCCGGGAGCAGACAAGCCCGTCAGGGCGCGTCAGCGGGTGTTGGCGGGTGTCGGGGCGCAGCCATGACCCAGTCACGTAGCGATAGCGGAGTGTATACTGGCTTAACTATGCGGCATCAGAGCAGATTGTACTGAGAGTGCACCATATGCGGTGTGAAATACCGCACAGATGCGTAAGGAGAAAATACCGCATCAGGCGCTCTTCCGCTTCCTCGCTCACTGACTCGCTGCGCTCGGTCGTTCGGCTGCGGCGAGCGGTATCAGCTCACTCAAAGGCGGTAATACGGTTATCCACAGAATCAGGGGATAACGCAGGAAAGAACATGTGAGCAAAAGGCCAGCAAAAGGCCAGGAACCGTAAAAAGGCCGCGTTGCTGGCGTTTTTCCATAGGCTCCGCCCCCCTGACGAGCATCACAAAAATCGACGCTCAAGTCAGAGGTGGCGAAACCCGACAGGACTATAAAGATACCAGGCGTTTCCCCCTGGAAGCTCCCTCGTGCGCTCTCCTGTTCCGACCCTGCCGCTTACCGGATACCTGTCCGCCTTTCTCCCTTCGGGAAGCGTGGCGCTTTCTCATAGCTCACGCTGTAGGTATCTCAGTTCGGTGTAGGTCGTTCGCTCCAAGCTGGGCTGTGTGCACGAACCCCCCGTTCAGCCCGACCGCTGCGCCTTATCCGGTAACTATCGTCTTGAGTCCAACCCGGTAAGACACGACTTATCGCCACTGGCAGCAGCCACTGGTAACAGGATTAGCAGAGCGAGGTATGTAGGCGGTGCTACAGAGTTCTTGAAGTGGTGGCCTAACTACGGCTACACTAGAAGGACAGTATTTGGTATCTGCGCTCTGCTGAAGCCAGTTACCTTCGGAAAAAGAGTTGGTAGCTCTTGATCCGGCAAACAAACCACCGCTGGTAGCGGTGGTTTTTTTGTTTGCAAGCAGCAGATTACGCGCAGAAAAAAAGGATCTCAAGAAGATCCTTTGATCTTTTCTACGGGGTCTGACGCTCAGTGGAACGAAAACTCACGTTAAGGGATTTTGGTCATGCATTCTAGGTACTAAAACAATTCATCCAGTAAAATATAATATTTTATTTTCTCCCAATCAGGCTTGATCCCCAGTAAGTCAAAAAATAGCTCGACATACTGTTCTTCCCCGATATCCTCCCTGATCGACCGGACGCAGAAGGCAATGTCATACCACTTGTCCGCCCTGCCGCTTCTCCCAAGATCAATAAAGCCACTTACTTTGCCATCTTTCACAAAGATGTTGCTGTCTCCCAGGTCGCCGTGGGAAAAGACAAGTTCCTCTTCGGGCTTTTCCGTCTTTAAAAAATCATACAGCTCGCGCGGATCTTTAAATGGAGTGTCTTCTTCCCAGTTTTCGCAATCCACATCGGCCAGATCGTTATTCAGTAAGTAATCCAATTCGGCTAAGCGGCTGTCTAAGCTATTCGTATAGGGACAATCCGATATGTCGATGGAGTGAAAGAGCCTGATGCACTCCGCATACAGCTCGATAATCTTTTCAGGGCTTTGTTCATCTTCATACTCTTCCGAGCAAAGGACGCCATCGGCCTCACTCATGAGCAGATTGCTCCAGCCATCATGCCGTTCAAAGTGCAGGACCTTTGGAACAGGCAGCTTTCCTTCCAGCCATAGCATCATGTCCTTTTCCCGTTCCACATCATAGGTGGTCCCTTTATACCGGCTGTCCGTCATTTTTAAATATAGGTTTTCATTTTCTCCCACCAGCTTATATACCTTAGCAGGAGACATTCCTTCCGTATCTTTTACGCAGCGGTATTTTTCGATCAGTTTTTTCAATTCCGGTGATATTCTCATTTTAGCCATTTATTATTTCCTTCCTCTTTTCTACAGTATTTAAAGATACCCCAAGAAGCTAATTATAACAAGACGAACTCCAATTCACTGTTCCTTGCATTCTAAAACCTTAAATACCAGAAAACAGCTTTTTCAAAGTTGTTTTCAAAGTTGGCGTATAACATAGTATCGACGGAGCCGATTTTGAAACCGCGGTGATCACAGGCAGCAACGCTCTGTCATCGTTACAATCAACATGCTACCCTCCGCGAGATCATCCGTGTTTCAAACCCGGCAGCTTAGTTGCCGTTCTTCCGAATAGCATCGGTAACATGAGCAAAGTCTGCCGCCTTACAACGGCTCTCCCGCTGACGCCGTCCCGGACTGATGGGCTGCCTGTATCGAGTGGTGATTTTGTGCCGAGCTGCCGGTCGGGGAGCTGTTGGCTGGCTGGTGGCAGGATATATTGTGGTGTAAACAAATTGACGCTTAGACAACTTAATAACACATTGCGGACGTTTTTAATGTACTGAATTAACGCCGAATTAATTCGGGGGATCTGGATTTTAGTACTGGATTTTGGTTTTAGGAATTAGAAATTTTATTGATAGAAGTATTTTACAAATACAAATACATACTAAGGGTTTCTTATATGCTCAACACATGAGCGAAACCCTATAGGAACCCTAATTCCCTTATCTGGGAACTACTCACACATTATTATGGAGAAACTCGAGCAGTGGAACGAAAACTCACGTTAAGGGATTTTGGTCATGCATTCTAGGTACTAAAACAATTCATCCAGTAAAATATAATATTTTATTTTCTCCCAATCAGGCTTGATCCCCAGTAAGTCAAAAAATAGCTCGACATACTGTTCTTCCCCGATATCCTCCCTGATCGACCGGACGCAGAAGGCAATGTCATACCACTTGTCCGCCCTGCCGCTTCTCCCAAGATCAATAAAGCCACTTACTTTGCCATCTTTCACAAAGATGTTGCTGTCTCCCAGGTCGCCGTGGGAAAAGACAAGTTCCTCTTCGGGCTTTTCCGTCTTTAAAAAATCATACAGCTCGCGCGGATCTTTAAATGGAGTGTCTTCTTCCCAGTTTTCGCAATCCACATCGGCCAGATCGTTATTCAGTAAGTAATCCAATTCGGCTAAGCGGCTGTCTAAGCTATTCGTATAGGGACAATCCGATATGTCGATGGAGTGAAAGAGCCTGATGCACTCCGCATACAGCTCGATAATCTTTTCAGGGCTTTGTTCATCTTCATACTCTTCCGAGCAAAGGACGCCATCGGCCTCACTCATGAGCAGATTGCTCCAGCCATCATGCCGTTCAAAGTGCAGGACCTTTGGAACAGGCAGCTTTCCTTCCAGCCATAGCATCATGTCCTTTTCCCGTTCCACATCATAGGTGGTCCCTTTATACCGGCTGTCCGTCATTTTTAAATATAGGTTTTCATTTTCTCCCACCAGCTTATATACCTTAGCAGGAGACATTCCTTCCGTATCTTTTACGCAGCGGTATTTTTCGATCAGTTTTTTCAATTCCGGTGATATTCTCATTTTAGCCATAGACGTCGCGGTGAGTTCAGGCTTTTTCATATCTCATTGCCCCCCCGGATCTGCGAAAGCTCGAGAGAGATAGATTTGTAGAGAGAGACTGGTGATTTCAGCGTGTCCTCTCCAAATGAAATGAACTTCCTTATATAGAGGAAGGTCTTGCGAAGGATAGTGGGATTGTGCGTCATCCCTTACGTCAGTGGAGATATCACATCAATCCACTTGCTTTGAAGACGTGGTTGGAACGTCTTCTTTTTCCACGATGCTCCTCGTGGGTGGGGGTCCATCTTTGGGACCACTGTCGGCAGAGGCATCTTGAACGATAGCCTTTCCTTTATCGCAATGATGGCATTTGTAGGTGCCACCTTCCTTTTCTACTGTCCTTTTGATGAAGTGACAGATAGCTGGGCAATGGAATCCGAGGAGGTTTCCCGATATTACCCTTTGTTGAAAAGTCTCAATAGCCCTTTGGTCTTCTGAGACTGTATCTTTGATATTCTTGGAGTAGACGAGAGTGTCGTGCTCCACCATGTTATCACATCAATCCACTTGCTTTGAAGACGTGGTTGGAACGTCTTCTTTTTCCACGATGCTCCTCGTGGGTGGGGGTCCATCTTTGGGACCACTGTCGGCAGAGGCATCTTGAACGATAGCCTTTCCTTTATCGCAATGATGGCATTTGTAGGTGCCACCTTCCTTTTCTACTGTCCTTTTGATGAAGTGACAGATAGCTGGGCAATGGAATCCGAGGAGGTTTCCCGATATTACCCTTTGTTGAAAAGTCTCAATAGCCCTTTGGTCTTCTGAGACTGTATCTTTGATATTCTTGGAGTAGACGAGAGTGTCGTGCTCCACCATGTTGGCAAGCTGCTCTAGCCAATACGCAAACCGCCTCTCCCCGCGCGTTGGCCGATTCATTAATGCAGCTGGCACGACAGGTTTCCCGACTGGAAAGCGGGCAGTGAGCGCAACGCAATTAATGTGAGTTAGCTCACTCATTAGGCACCCCAGGCTTTACACTTTATGCTTCCGGCTCGTATGTTGTGTGGAATTGTGAGCGGATAACAATTTCACACAGGAAACAGCTATGACCATGATTACGAAGCTGCCCTTGAATTACGCGGCGCGCCAGGGCCCTCAGGGTCCCAATTGTACACGCGTGGCGAATTCGGTGACCGACGACAAGTCACGCCGCGAGGCCTGTGCGCACTGTTAACACTAGTCACATTTAAATGCAAGTGACGATGTCGAAGCATTTCGGCGGCGGCCGCATGGAGTCAAAGATTCAAATAGAGGACCTAACAGAACTCGCCGTAAAGACTGGCGAACAGTTCATACAGAGTCTCTTACGACTCAATGACAAGAAGAAAATCTTCGTCAACATGGTGGAGCACGACACACTTGTCTACTCCAAAAATATCAAAGATACAGTCTCAGAAGACCAAAGGGCAATTGAGACTTTTCAACAAAGGGTAATATCCGGAAACCTCCTCGGATTCCATTGCCCAGCTATCTGTCACTTTATTGTGAAGATAGTGGAAAAGGAAGGTGGCTCCTACAAATGCCATCATTGCGATAAAGGAAAGGCCATCGTTGAAGATGCCTCTGCCGACAGTGGTCCCAAAGATGGACCCCCACCCACGAGGAGCATCGTGGAAAAAGAAGACGTTCCAACCACGTCTTCAAAGCAAGTGGATTGATGTGATATCTCCACTGACGTAAGGGATGACGCACAATCCCACTATCCTTCGCAAGACCCTTCCTCTATATAAGGAAGTTCATTTCATTTGGAGAGGACACTCGGAGAGGTAGTATTTTTACAACAATTACCAACAACAACAACAAACAACAACAACATTACATTTTACATTCTACAACTACAAGCTTCTAGAGTCGACCTGCAGATTGGTAAGGAAATAATTATTTTCTTTTTTCCTTTTAGTATAAAATAGTTAAGTGATGTTAATTAGTATGATTATAATAATATAGTTGTTATAATTGTGAAAAAATAATTTATAAATATATTGTTTACATAAACAACATAGTAATGTAAAAAAATATGACAAGTGATGTGTAAGACGAAGAAGATAAAAGTTGAGAGTAAGTATATTATTTTTAATGAATTTGATCGAACATGTAAGATGATATACTAGCATTAATATTTGTTTTAATCATAATAGTAATTCTAGCTGGTTTGATGAATTAAATATCAATGATAAAATACTATAGTAAAAATAAGAATAAATAAATTAAAATAATATTTTTTTATGATTAATAGTTTATTATATAATTAAATATCTATACCATTACTAAATATTTTAGTTTAAAAGTTAATAAATATTTTGTTAGAAATTCCAATCTGCTTGTAATTTATCAATAAACAAAATATTAAATAACAAGCTAAAGTAACAAATAATATCAAACTAATAGAAACAGTAATCTAATGTAACAAAACATAATCTAATGCTAATATAACAAAGCGCAAGATCTATCAATTTTATATAGTATTATTTTTCAATCAACATTCTTATTAATTTCTAAATAATACTTGTAGTTTTATTAACTTCTAAATGGATTGACTATTAATTAAATGAATTAGTCGAACATGAATAAACAAGGTAACATGATAGATCATGTCATTGTGTTATCATTGATCTTACATTTGGATTGATTACAGTTGGGAAAGTAAGCGGCCGCTGCGGTACCCGGGGAGCTCGAATTTCCCCGATCGTTCAAACATTTGGCAATAAAGTTTCTTAAGATTGAATCCTGTTGCCGGTCTTGCGATGATTATCATATAATTTCTGTTGAATTACGTTAAGCATGTAATAATTAACATGTAATGCATGACGTTATTTATGAGATGGGTTTTTATGATTAGAGTCCCGCAATTATACATTTAATACGCGATAGAAAACAAAATATAGCGCGCAAACTAGGATAAATTATCGCGCGCGGTGTCATCTATGTTACTAGATCGGGAATTAGATATCGTTGAAACAGTACTAACCTTAAGGGATCCAGCTTGGCACTGGCCGTCGTTTTACAACGTCGTGACTGGGAAAACCCTGGCGTTACCCAACTTAATCGCCTTGCAGCACATCCCCCTTTCGCCAGCTGGCGTAATAGCGAAGAGGCCCGCACCGATCGCCCTTCCCAACAGTTGCGCAGCCTGAATGGCGAATGCTAGAGCAGCTTGAGCTTGGATCAGATTGTCGTTTCCCGCCTTCAGTTTAGCTTCATGGAGTCAAAGATTCAAATAGAGGACCTAACAGAACTCGCCGTAAAGACTGGCGAACAGTTCATACAGAGTCTCTTACGACTCAATGACAAGAAGAAAATCTTCGTCAACATGGTGGAGCACGACACACTTGTCTACTCCAAAAATATCAAAGATACAGTCTCAGAAGACCAAAGGGCAATTGAGACTTTTCAACAAAGGGTAATATCCGGAAACCTCCTCGGATTCCATTGCCCAGCTATCTGTCACTTTATTGTGAAGATAGTGGAAAAGGAAGGTGGCTCCTACAAATGCCATCATTGCGATAAAGGAAAGGCCATCGTTGAAGATGCCTCTGCCGACAGTGGTCCCAAAGATGGACCCCCACCCACGAGGAGCATCGTGGAAAAAGAAGACGTTCCAACCACGTCTTCAAAGCAAGTGGATTGATGTGATATCTCCACTGACGTAAGGGATGACGCACAATCCCACTATCCTTCGCAAGACCCTTCCTCTATATAAGGAAGTTCATTTCATTTGGAGAGAACACGGGGGACTCTTGACCATGGTAG

**2.2 Sequence of pcambia1301S**

ATCTGAGGGTAAATTTCTAGTTTTTCTCCTTCATTTTCTTGGTTAGGACCCTTTTCTCTTTTTATTTTTTTGAGCTTTGATCTTTCTTTAAACTGATCTATTTTTTAATTGATTGGTTATGGTGTAAATATTACATAGCTTTAACTGATAATCTGATTACTTTATTTCGTGTGTCTATGATGATGATGATAGTTACAGAACCGACGACTCGTCCGTCCTGTAGAAACCCCAACCCGTGAAATCAAAAAACTCGACGGCCTGTGGGCATTCAGTCTGGATCGCGAAAACTGTGGAATTGATCAGCGTTGGTGGGAAAGCGCGTTACAAGAAAGCCGGGCAATTGCTGTGCCAGGCAGTTTTAACGATCAGTTCGCCGATGCAGATATTCGTAATTATGCGGGCAACGTCTGGTATCAGCGCGAAGTCTTTATACCGAAAGGTTGGGCAGGCCAGCGTATCGTGCTGCGTTTCGATGCGGTCACTCATTACGGCAAAGTGTGGGTCAATAATCAGGAAGTGATGGAGCATCAGGGCGGCTATACGCCATTTGAAGCCGATGTCACGCCGTATGTTATTGCCGGGAAAAGTGTACGTATCACCGTTTGTGTGAACAACGAACTGAACTGGCAGACTATCCCGCCGGGAATGGTGATTACCGACGAAAACGGCAAGAAAAAGCAGTCTTACTTCCATGATTTCTTTAACTATGCCGGAATCCATCGCAGCGTAATGCTCTACACCACGCCGAACACCTGGGTGGACGATATCACCGTGGTGACGCATGTCGCGCAAGACTGTAACCACGCGTCTGTTGACTGGCAGGTGGTGGCCAATGGTGATGTCAGCGTTGAACTGCGTGATGCGGATCAACAGGTGGTTGCAACTGGACAAGGCACTAGCGGGACTTTGCAAGTGGTGAATCCGCACCTCTGGCAACCGGGTGAAGGTTATCTCTATGAACTCGAAGTCACAGCCAAAAGCCAGACAGAGTCTGATATCTACCCGCTTCGCGTCGGCATCCGGTCAGTGGCAGTGAAGGGCCAACAGTTCCTGATTAACCACAAACCGTTCTACTTTACTGGCTTTGGTCGTCATGAAGATGCGGACTTACGTGGCAAAGGATTCGATAACGTGCTGATGGTGCACGACCACGCATTAATGGACTGGATTGGGGCCAACTCCTACCGTACCTCGCATTACCCTTACGCTGAAGAGATGCTCGACTGGGCAGATGAACATGGCATCGTGGTGATTGATGAAACTGCTGCTGTCGGCTTTCAGCTGTCTTTAGGCATTGGTTTCGAAGCGGGCAACAAGCCGAAAGAACTGTACAGCGAAGAGGCAGTCAACGGGGAAACTCAGCAAGCGCACTTACAGGCGATTAAAGAGCTGATAGCGCGTGACAAAAACCACCCAAGCGTGGTGATGTGGAGTATTGCCAACGAACCGGATACCCGTCCGCAAGGTGCACGGGAATATTTCGCGCCACTGGCGGAAGCAACGCGTAAACTCGACCCGACGCGTCCGATCACCTGCGTCAATGTAATGTTCTGCGACGCTCACACCGATACCATCAGCGATCTCTTTGATGTGCTGTGCCTGAACCGTTATTACGGATGGTATGTCCAAAGCGGCGATTTGGAAACGGCAGAGAAGGTACTGGAAAAAGAACTTCTGGCCTGGCAGGAGAAACTGCATCAGCCGATTATCATCACCGAATACGGCGTGGATACGTTAGCCGGGCTGCACTCAATGTACACCGACATGTGGAGTGAAGAGTATCAGTGTGCATGGCTGGATATGTATCACCGCGTCTTTGATCGCGTCAGCGCCGTCGTCGGTGAACAGGTATGGAATTTCGCCGATTTTGCGACCTCGCAAGGCATATTGCGCGTTGGCGGTAACAAGAAAGGGATCTTCACTCGCGACCGCAAACCGAAGTCGGCGGCTTTTCTGCTGCAAAAACGCTGGACTGGCATGAACTTCGGTGAAAAACCGCAGCAGGGAGGCAAACAAGCTAGCCACCACCACCACCACCACGTGTGAATTACAGGTGACCAGCTCGAATTTCCCCGATCGTTCAAACATTTGGCAATAAAGTTTCTTAAGATTGAATCCTGTTGCCGGTCTTGCGATGATTATCATATAATTTCTGTTGAATTACGTTAAGCATGTAATAATTAACATGTAATGCATGACGTTATTTATGAGATGGGTTTTTATGATTAGAGTCCCGCAATTATACATTTAATACGCGATAGAAAACAAAATATAGCGCGCAAACTAGGATAAATTATCGCGCGCGGTGTCATCTATGTTACTAGATCGGGAATTAAACTATCAGTGTTTGACAGGATATATTGGCGGGTAAACCTAAGAGAAAAGAGCGTTTATTAGAATAACGGATATTTAAAAGGGCGTGAAAAGGTTTATCCGTTCGTCCATTTGTATGTGCATGCCAACCACAGGGTTCCCCTCGGGATCAAAGTACTTTGATCCAACCCCTCCGCTGCTATAGTGCAGTCGGCTTCTGACGTTCAGTGCAGCCGTCTTCTGAAAACGACATGTCGCACAAGTCCTAAGTTACGCGACAGGCTGCCGCCCTGCCCTTTTCCTGGCGTTTTCTTGTCGCGTGTTTTAGTCGCATAAAGTAGAATACTTGCGACTAGAACCGGAGACATTACGCCATGAACAAGAGCGCCGCCGCTGGCCTGCTGGGCTATGCCCGCGTCAGCACCGACGACCAGGACTTGACCAACCAACGGGCCGAACTGCACGCGGCCGGCTGCACCAAGCTGTTTTCCGAGAAGATCACCGGCACCAGGCGCGACCGCCCGGAGCTGGCCAGGATGCTTGACCACCTACGCCCTGGCGACGTTGTGACAGTGACCAGGCTAGACCGCCTGGCCCGCAGCACCCGCGACCTACTGGACATTGCCGAGCGCATCCAGGAGGCCGGCGCGGGCCTGCGTAGCCTGGCAGAGCCGTGGGCCGACACCACCACGCCGGCCGGCCGCATGGTGTTGACCGTGTTCGCCGGCATTGCCGAGTTCGAGCGTTCCCTAATCATCGACCGCACCCGGAGCGGGCGCGAGGCCGCCAAGGCCCGAGGCGTGAAGTTTGGCCCCCGCCCTACCCTCACCCCGGCACAGATCGCGCACGCCCGCGAGCTGATCGACCAGGAAGGCCGCACCGTGAAAGAGGCGGCTGCACTGCTTGGCGTGCATCGCTCGACCCTGTACCGCGCACTTGAGCGCAGCGAGGAAGTGACGCCCACCGAGGCCAGGCGGCGCGGTGCCTTCCGTGAGGACGCATTGACCGAGGCCGACGCCCTGGCGGCCGCCGAGAATGAACGCCAAGAGGAACAAGCATGAAACCGCACCAGGACGGCCAGGACGAACCGTTTTTCATTACCGAAGAGATCGAGGCGGAGATGATCGCGGCCGGGTACGTGTTCGAGCCGCCCGCGCACGTCTCAACCGTGCGGCTGCATGAAATCCTGGCCGGTTTGTCTGATGCCAAGCTGGCGGCCTGGCCGGCCAGCTTGGCCGCTGAAGAAACCGAGCGCCGCCGTCTAAAAAGGTGATGTGTATTTGAGTAAAACAGCTTGCGTCATGCGGTCGCTGCGTATATGATGCGATGAGTAAATAAACAAATACGCAAGGGGAACGCATGAAGGTTATCGCTGTACTTAACCAGAAAGGCGGGTCAGGCAAGACGACCATCGCAACCCATCTAGCCCGCGCCCTGCAACTCGCCGGGGCCGATGTTCTGTTAGTCGATTCCGATCCCCAGGGCAGTGCCCGCGATTGGGCGGCCGTGCGGGAAGATCAACCGCTAACCGTTGTCGGCATCGACCGCCCGACGATTGACCGCGACGTGAAGGCCATCGGCCGGCGCGACTTCGTAGTGATCGACGGAGCGCCCCAGGCGGCGGACTTGGCTGTGTCCGCGATCAAGGCAGCCGACTTCGTGCTGATTCCGGTGCAGCCAAGCCCTTACGACATATGGGCCACCGCCGACCTGGTGGAGCTGGTTAAGCAGCGCATTGAGGTCACGGATGGAAGGCTACAAGCGGCCTTTGTCGTGTCGCGGGCGATCAAAGGCACGCGCATCGGCGGTGAGGTTGCCGAGGCGCTGGCCGGGTACGAGCTGCCCATTCTTGAGTCCCGTATCACGCAGCGCGTGAGCTACCCAGGCACTGCCGCCGCCGGCACAACCGTTCTTGAATCAGAACCCGAGGGCGACGCTGCCCGCGAGGTCCAGGCGCTGGCCGCTGAAATTAAATCAAAACTCATTTGAGTTAATGAGGTAAAGAGAAAATGAGCAAAAGCACAAACACGCTAAGTGCCGGCCGTCCGAGCGCACGCAGCAGCAAGGCTGCAACGTTGGCCAGCCTGGCAGACACGCCAGCCATGAAGCGGGTCAACTTTCAGTTGCCGGCGGAGGATCACACCAAGCTGAAGATGTACGCGGTACGCCAAGGCAAGACCATTACCGAGCTGCTATCTGAATACATCGCGCAGCTACCAGAGTAAATGAGCAAATGAATAAATGAGTAGATGAATTTTAGCGGCTAAAGGAGGCGGCATGGAAAATCAAGAACAACCAGGCACCGACGCCGTGGAATGCCCCATGTGTGGAGGAACGGGCGGTTGGCCAGGCGTAAGCGGCTGGGTTGTCTGCCGGCCCTGCAATGGCACTGGAACCCCCAAGCCCGAGGAATCGGCGTGACGGTCGCAAACCATCCGGCCCGGTACAAATCGGCGCGGCGCTGGGTGATGACCTGGTGGAGAAGTTGAAGGCCGCGCAGGCCGCCCAGCGGCAACGCATCGAGGCAGAAGCACGCCCCGGTGAATCGTGGCAAGCGGCCGCTGATCGAATCCGCAAAGAATCCCGGCAACCGCCGGCAGCCGGTGCGCCGTCGATTAGGAAGCCGCCCAAGGGCGACGAGCAACCAGATTTTTTCGTTCCGATGCTCTATGACGTGGGCACCCGCGATAGTCGCAGCATCATGGACGTGGCCGTTTTCCGTCTGTCGAAGCGTGACCGACGAGCTGGCGAGGTGATCCGCTACGAGCTTCCAGACGGGCACGTAGAGGTTTCCGCAGGGCCGGCCGGCATGGCCAGTGTGTGGGATTACGACCTGGTACTGATGGCGGTTTCCCATCTAACCGAATCCATGAACCGATACCGGGAAGGGAAGGGAGACAAGCCCGGCCGCGTGTTCCGTCCACACGTTGCGGACGTACTCAAGTTCTGCCGGCGAGCCGATGGCGGAAAGCAGAAAGACGACCTGGTAGAAACCTGCATTCGGTTAAACACCACGCACGTTGCCATGCAGCGTACGAAGAAGGCCAAGAACGGCCGCCTGGTGACGGTATCCGAGGGTGAAGCCTTGATTAGCCGCTACAAGATCGTAAAGAGCGAAACCGGGCGGCCGGAGTACATCGAGATCGAGCTAGCTGATTGGATGTACCGCGAGATCACAGAAGGCAAGAACCCGGACGTGCTGACGGTTCACCCCGATTACTTTTTGATCGATCCCGGCATCGGCCGTTTTCTCTACCGCCTGGCACGCCGCGCCGCAGGCAAGGCAGAAGCCAGATGGTTGTTCAAGACGATCTACGAACGCAGTGGCAGCGCCGGAGAGTTCAAGAAGTTCTGTTTCACCGTGCGCAAGCTGATCGGGTCAAATGACCTGCCGGAGTACGATTTGAAGGAGGAGGCGGGGCAGGCTGGCCCGATCCTAGTCATGCGCTACCGCAACCTGATCGAGGGCGAAGCATCCGCCGGTTCCTAATGTACGGAGCAGATGCTAGGGCAAATTGCCCTAGCAGGGGAAAAAGGTCGAAAAGGTCTCTTTCCTGTGGATAGCACGTACATTGGGAACCCAAAGCCGTACATTGGGAACCGGAACCCGTACATTGGGAACCCAAAGCCGTACATTGGGAACCGGTCACACATGTAAGTGACTGATATAAAAGAGAAAAAAGGCGATTTTTCCGCCTAAAACTCTTTAAAACTTATTAAAACTCTTAAAACCCGCCTGGCCTGTGCATAACTGTCTGGCCAGCGCACAGCCGAAGAGCTGCAAAAAGCGCCTACCCTTCGGTCGCTGCGCTCCCTACGCCCCGCCGCTTCGCGTCGGCCTATCGCGGCCGCTGGCCGCTCAAAAATGGCTGGCCTACGGCCAGGCAATCTACCAGGGCGCGGACAAGCCGCGCCGTCGCCACTCGACCGCCGGCGCCCACATCAAGGCACCCTGCCTCGCGCGTTTCGGTGATGACGGTGAAAACCTCTGACACATGCAGCTCCCGGAGACGGTCACAGCTTGTCTGTAAGCGGATGCCGGGAGCAGACAAGCCCGTCAGGGCGCGTCAGCGGGTGTTGGCGGGTGTCGGGGCGCAGCCATGACCCAGTCACGTAGCGATAGCGGAGTGTATACTGGCTTAACTATGCGGCATCAGAGCAGATTGTACTGAGAGTGCACCATATGCGGTGTGAAATACCGCACAGATGCGTAAGGAGAAAATACCGCATCAGGCGCTCTTCCGCTTCCTCGCTCACTGACTCGCTGCGCTCGGTCGTTCGGCTGCGGCGAGCGGTATCAGCTCACTCAAAGGCGGTAATACGGTTATCCACAGAATCAGGGGATAACGCAGGAAAGAACATGTGAGCAAAAGGCCAGCAAAAGGCCAGGAACCGTAAAAAGGCCGCGTTGCTGGCGTTTTTCCATAGGCTCCGCCCCCCTGACGAGCATCACAAAAATCGACGCTCAAGTCAGAGGTGGCGAAACCCGACAGGACTATAAAGATACCAGGCGTTTCCCCCTGGAAGCTCCCTCGTGCGCTCTCCTGTTCCGACCCTGCCGCTTACCGGATACCTGTCCGCCTTTCTCCCTTCGGGAAGCGTGGCGCTTTCTCATAGCTCACGCTGTAGGTATCTCAGTTCGGTGTAGGTCGTTCGCTCCAAGCTGGGCTGTGTGCACGAACCCCCCGTTCAGCCCGACCGCTGCGCCTTATCCGGTAACTATCGTCTTGAGTCCAACCCGGTAAGACACGACTTATCGCCACTGGCAGCAGCCACTGGTAACAGGATTAGCAGAGCGAGGTATGTAGGCGGTGCTACAGAGTTCTTGAAGTGGTGGCCTAACTACGGCTACACTAGAAGGACAGTATTTGGTATCTGCGCTCTGCTGAAGCCAGTTACCTTCGGAAAAAGAGTTGGTAGCTCTTGATCCGGCAAACAAACCACCGCTGGTAGCGGTGGTTTTTTTGTTTGCAAGCAGCAGATTACGCGCAGAAAAAAAGGATCTCAAGAAGATCCTTTGATCTTTTCTACGGGGTCTGACGCTCAGTGGAACGAAAACTCACGTTAAGGGATTTTGGTCATGCATTCTAGGTACTAAAACAATTCATCCAGTAAAATATAATATTTTATTTTCTCCCAATCAGGCTTGATCCCCAGTAAGTCAAAAAATAGCTCGACATACTGTTCTTCCCCGATATCCTCCCTGATCGACCGGACGCAGAAGGCAATGTCATACCACTTGTCCGCCCTGCCGCTTCTCCCAAGATCAATAAAGCCACTTACTTTGCCATCTTTCACAAAGATGTTGCTGTCTCCCAGGTCGCCGTGGGAAAAGACAAGTTCCTCTTCGGGCTTTTCCGTCTTTAAAAAATCATACAGCTCGCGCGGATCTTTAAATGGAGTGTCTTCTTCCCAGTTTTCGCAATCCACATCGGCCAGATCGTTATTCAGTAAGTAATCCAATTCGGCTAAGCGGCTGTCTAAGCTATTCGTATAGGGACAATCCGATATGTCGATGGAGTGAAAGAGCCTGATGCACTCCGCATACAGCTCGATAATCTTTTCAGGGCTTTGTTCATCTTCATACTCTTCCGAGCAAAGGACGCCATCGGCCTCACTCATGAGCAGATTGCTCCAGCCATCATGCCGTTCAAAGTGCAGGACCTTTGGAACAGGCAGCTTTCCTTCCAGCCATAGCATCATGTCCTTTTCCCGTTCCACATCATAGGTGGTCCCTTTATACCGGCTGTCCGTCATTTTTAAATATAGGTTTTCATTTTCTCCCACCAGCTTATATACCTTAGCAGGAGACATTCCTTCCGTATCTTTTACGCAGCGGTATTTTTCGATCAGTTTTTTCAATTCCGGTGATATTCTCATTTTAGCCATTTATTATTTCCTTCCTCTTTTCTACAGTATTTAAAGATACCCCAAGAAGCTAATTATAACAAGACGAACTCCAATTCACTGTTCCTTGCATTCTAAAACCTTAAATACCAGAAAACAGCTTTTTCAAAGTTGTTTTCAAAGTTGGCGTATAACATAGTATCGACGGAGCCGATTTTGAAACCGCGGTGATCACAGGCAGCAACGCTCTGTCATCGTTACAATCAACATGCTACCCTCCGCGAGATCATCCGTGTTTCAAACCCGGCAGCTTAGTTGCCGTTCTTCCGAATAGCATCGGTAACATGAGCAAAGTCTGCCGCCTTACAACGGCTCTCCCGCTGACGCCGTCCCGGACTGATGGGCTGCCTGTATCGAGTGGTGATTTTGTGCCGAGCTGCCGGTCGGGGAGCTGTTGGCTGGCTGGTGGCAGGATATATTGTGGTGTAAACAAATTGACGCTTAGACAACTTAATAACACATTGCGGACGTTTTTAATGTACTGAATTAACGCCGAATTAATTCGGGGGATCTGGATTTTAGTACTGGATTTTGGTTTTAGGAATTAGAAATTTTATTGATAGAAGTATTTTACAAATACAAATACATACTAAGGGTTTCTTATATGCTCAACACATGAGCGAAACCCTATAGGAACCCTAATTCCCTTATCTGGGAACTACTCACACATTATTATGGAGAAACTCGAGCAGTGGAACGAAAACTCACGTTAAGGGATTTTGGTCATGCATTCTAGGTACTAAAACAATTCATCCAGTAAAATATAATATTTTATTTTCTCCCAATCAGGCTTGATCCCCAGTAAGTCAAAAAATAGCTCGACATACTGTTCTTCCCCGATATCCTCCCTGATCGACCGGACGCAGAAGGCAATGTCATACCACTTGTCCGCCCTGCCGCTTCTCCCAAGATCAATAAAGCCACTTACTTTGCCATCTTTCACAAAGATGTTGCTGTCTCCCAGGTCGCCGTGGGAAAAGACAAGTTCCTCTTCGGGCTTTTCCGTCTTTAAAAAATCATACAGCTCGCGCGGATCTTTAAATGGAGTGTCTTCTTCCCAGTTTTCGCAATCCACATCGGCCAGATCGTTATTCAGTAAGTAATCCAATTCGGCTAAGCGGCTGTCTAAGCTATTCGTATAGGGACAATCCGATATGTCGATGGAGTGAAAGAGCCTGATGCACTCCGCATACAGCTCGATAATCTTTTCAGGGCTTTGTTCATCTTCATACTCTTCCGAGCAAAGGACGCCATCGGCCTCACTCATGAGCAGATTGCTCCAGCCATCATGCCGTTCAAAGTGCAGGACCTTTGGAACAGGCAGCTTTCCTTCCAGCCATAGCATCATGTCCTTTTCCCGTTCCACATCATAGGTGGTCCCTTTATACCGGCTGTCCGTCATTTTTAAATATAGGTTTTCATTTTCTCCCACCAGCTTATATACCTTAGCAGGAGACATTCCTTCCGTATCTTTTACGCAGCGGTATTTTTCGATCAGTTTTTTCAATTCCGGTGATATTCTCATTTTAGCCATAGACGTCGCGGTGAGTTCAGGCTTTTTCATATCTCATTGCCCCCCCGGATCTGCGAAAGCTCGAGAGAGATAGATTTGTAGAGAGAGACTGGTGATTTCAGCGTGTCCTCTCCAAATGAAATGAACTTCCTTATATAGAGGAAGGTCTTGCGAAGGATAGTGGGATTGTGCGTCATCCCTTACGTCAGTGGAGATATCACATCAATCCACTTGCTTTGAAGACGTGGTTGGAACGTCTTCTTTTTCCACGATGCTCCTCGTGGGTGGGGGTCCATCTTTGGGACCACTGTCGGCAGAGGCATCTTGAACGATAGCCTTTCCTTTATCGCAATGATGGCATTTGTAGGTGCCACCTTCCTTTTCTACTGTCCTTTTGATGAAGTGACAGATAGCTGGGCAATGGAATCCGAGGAGGTTTCCCGATATTACCCTTTGTTGAAAAGTCTCAATAGCCCTTTGGTCTTCTGAGACTGTATCTTTGATATTCTTGGAGTAGACGAGAGTGTCGTGCTCCACCATGTTATCACATCAATCCACTTGCTTTGAAGACGTGGTTGGAACGTCTTCTTTTTCCACGATGCTCCTCGTGGGTGGGGGTCCATCTTTGGGACCACTGTCGGCAGAGGCATCTTGAACGATAGCCTTTCCTTTATCGCAATGATGGCATTTGTAGGTGCCACCTTCCTTTTCTACTGTCCTTTTGATGAAGTGACAGATAGCTGGGCAATGGAATCCGAGGAGGTTTCCCGATATTACCCTTTGTTGAAAAGTCTCAATAGCCCTTTGGTCTTCTGAGACTGTATCTTTGATATTCTTGGAGTAGACGAGAGTGTCGTGCTCCACCATGTTGGCAAGCTGCTCTAGCCAATACGCAAACCGCCTCTCCCCGCGCGTTGGCCGATTCATTAATGCAGCTGGCACGACAGGTTTCCCGACTGGAAAGCGGGCAGTGAGCGCAACGCAATTAATGTGAGTTAGCTCACTCATTAGGCACCCCAGGCTTTACACTTTATGCTTCCGGCTCGTATGTTGTGTGGAATTGTGAGCGGATAACAATTTCACACAGGAAACAGCTATGACCATGATTACGAAGCTGCCCTTGAATTACGCGGCGCGCCAGGGCCCTCAGGGTCCCAATTGTACACGCGTGGCGAATTCGGTGACCGACGACAAGTCACGCCGCGAGGCCTGTGCGCACTGTTAACACTAGTCACGCAAGTGACGATGTCGAAGCATTTCGGCGGCGGCCGCATGGAGTCAAAGATTCAAATAGAGGACCTAACAGAACTCGCCGTAAAGACTGGCGAACAGTTCATACAGAGTCTCTTACGACTCAATGACAAGAAGAAAATCTTCGTCAACATGGTGGAGCACGACACACTTGTCTACTCCAAAAATATCAAAGATACAGTCTCAGAAGACCAAAGGGCAATTGAGACTTTTCAACAAAGGGTAATATCCGGAAACCTCCTCGGATTCCATTGCCCAGCTATCTGTCACTTTATTGTGAAGATAGTGGAAAAGGAAGGTGGCTCCTACAAATGCCATCATTGCGATAAAGGAAAGGCCATCGTTGAAGATGCCTCTGCCGACAGTGGTCCCAAAGATGGACCCCCACCCACGAGGAGCATCGTGGAAAAAGAAGACGTTCCAACCACGTCTTCAAAGCAAGTGGATTGATGTGATATCTCCACTGACGTAAGGGATGACGCACAATCCCACTATCCTTCGCAAGACCCTTCCTCTATATAAGGAAGTTCATTTCATTTGGAGAGGACACTCGGAGAGGTAGTATTTTTACAACAATTACCAACAACAACAACAAACAACAACAACATTACATTTTACATTCTACAACTACAAGCTTCTAGAGTCGACCTGCAGATTGGTAAGGAAATAATTATTTTCTTTTTTCCTTTTAGTATAAAATAGTTAAGTGATGTTAATTAGTATGATTATAATAATATAGTTGTTATAATTGTGAAAAAATAATTTATAAATATATTGTTTACATAAACAACATAGTAATGTAAAAAAATATGACAAGTGATGTGTAAGACGAAGAAGATAAAAGTTGAGAGTAAGTATATTATTTTTAATGAATTTGATCGAACATGTAAGATGATATACTAGCATTAATATTTGTTTTAATCATAATAGTAATTCTAGCTGGTTTGATGAATTAAATATCAATGATAAAATACTATAGTAAAAATAAGAATAAATAAATTAAAATAATATTTTTTTATGATTAATAGTTTATTATATAATTAAATATCTATACCATTACTAAATATTTTAGTTTAAAAGTTAATAAATATTTTGTTAGAAATTCCAATCTGCTTGTAATTTATCAATAAACAAAATATTAAATAACAAGCTAAAGTAACAAATAATATCAAACTAATAGAAACAGTAATCTAATGTAACAAAACATAATCTAATGCTAATATAACAAAGCGCAAGATCTATCAATTTTATATAGTATTATTTTTCAATCAACATTCTTATTAATTTCTAAATAATACTTGTAGTTTTATTAACTTCTAAATGGATTGACTATTAATTAAATGAATTAGTCGAACATGAATAAACAAGGTAACATGATAGATCATGTCATTGTGTTATCATTGATCTTACATTTGGATTGATTACAGTTGGGAAAGTAAGCGGCCGCTGCGGTACCCGGGGAGCTCGAATTTCCCCGATCGTTCAAACATTTGGCAATAAAGTTTCTTAAGATTGAATCCTGTTGCCGGTCTTGCGATGATTATCATATAATTTCTGTTGAATTACGTTAAGCATGTAATAATTAACATGTAATGCATGACGTTATTTATGAGATGGGTTTTTATGATTAGAGTCCCGCAATTATACATTTAATACGCGATAGAAAACAAAATATAGCGCGCAAACTAGGATAAATTATCGCGCGCGGTGTCATCTATGTTACTAGATCGGGAATTAGATATCAGTACTAATTTAAATAGTTTAAACGCTTAAGGGATCCAGCTTGGCACTGGCCGTCGTTTTACAACGTCGTGACTGGGAAAACCCTGGCGTTACCCAACTTAATCGCCTTGCAGCACATCCCCCTTTCGCCAGCTGGCGTAATAGCGAAGAGGCCCGCACCGATCGCCCTTCCCAACAGTTGCGCAGCCTGAATGGCGAATGCTAGAGCAGCTTGAGCTTGGATCAGATTGTCGTTTCCCGCCTTCAGTTTAGCTTCATGGAGTCAAAGATTCAAATAGAGGACCTAACAGAACTCGCCGTAAAGACTGGCGAACAGTTCATACAGAGTCTCTTACGACTCAATGACAAGAAGAAAATCTTCGTCAACATGGTGGAGCACGACACACTTGTCTACTCCAAAAATATCAAAGATACAGTCTCAGAAGACCAAAGGGCAATTGAGACTTTTCAACAAAGGGTAATATCCGGAAACCTCCTCGGATTCCATTGCCCAGCTATCTGTCACTTTATTGTGAAGATAGTGGAAAAGGAAGGTGGCTCCTACAAATGCCATCATTGCGATAAAGGAAAGGCCATCGTTGAAGATGCCTCTGCCGACAGTGGTCCCAAAGATGGACCCCCACCCACGAGGAGCATCGTGGAAAAAGAAGACGTTCCAACCACGTCTTCAAAGCAAGTGGATTGATGTGATATCTCCACTGACGTAAGGGATGACGCACAATCCCACTATCCTTCGCAAGACCCTTCCTCTATATAAGGAAGTTCATTTCATTTGGAGAGAACACGGGGGACTCTTGACCATGGTAG

**Supplementary appendix 3**

**ABA receptor (PYR/PYL/RCAR) sequences in tomato and other species**

>Solyc08g076960.1.1(SlRCAR1)

MDNKPETSLDNPVHQRSEPGSETGSSLSTITTHHLTVPPGLTPEEFQELSSSIAEFHSYRINPGQCSSLLAQRIHAPVETVWTVVRRFDKPQTYKHFIKSCSVGEDFRMTVGSTRDVTVISGLPAAtSTERLDILDDDRHVTGFSIIGGEHRLRNYRSVTTVHGFERDGEIWTVVLESYVVDVPEGNTEEDTRLFADTVVKLNLQKLASVTETLAREAGNGSVNSRDASHR

>Solyc06g061180.1.1(SlRCAR2)

MEQSDNSTTHVHQEAEEEPNPTHQFMLPPGLTPEESDELKSSVTDFHSYQVNSSQCSSLLAQRIHAPPHVVWPVVRRFEKPQIYKHFIKSCSVAENFSMVVGAtRDVNVISGLPANTSTERLDLLDDEKYVTGFSIIGGEHRLKNYRSVTSVHGFERHGRIWTVVLESYVVDVPEGNTEEDTRLFADTVVKLNLQKLASVAEAIALGGNGEIS

>Solyc12g095970.1.1(SlRCAR3)

MDGDRQLLVPQGLTQEEFVELEPLIRNYHTFEDLPNTCTSLVTQRIDAPVDVVWPFIRRFDNPEKYKHFIKSCRIVSGDGGVGSIREVTVVSGIPASTSTERLEILDDEKHILSFRVVGGEHRLTNYKSVTSVNEFKKNGKIYTIVLESYIVDIPEGNTGEDTKMFTDTVVKLNLQKLALVAMSTMHGHE

>Solyc08g065410.1.1(SlRCAR4)

MRIEAPANVVWPFVRRFDNPQKYKHFIKSCKMTGDGGVGSIREVSVVSGIPASTSTERLEILDDEKHILSFKVVGGEHRLNNYKSVTSVNEFEKNGKAYTIVLESYIVDIPQGNTGEDTKMFTDTVVKLNLQKLGVVAMAAMHGHE

>Solyc10g085310.1.1(SlRCAR5)

MPPSSSDSSVLLQRISSNNTHDFAYKQSHHQLQRRMPIPCSTEVPDSVSRYHTHTVSPDQCCSAVIQRISAPVSTVWSVVRRFDNPQAYKHFVKSCHVVVGDGDVGTLREVRVISGLPAASSTERLEILDDERHVISFSVVGGDHKLANYRSVTTLHTEPSSGNEAAAETIVVESYVVDVPPGNTREETCVFVDTIVKCNLQSLSQIAQNSAR

>Solyc03g095780.1.1(SlRCAR6)

MPCSVQLQRINPTTTTTLAGNFHKPPQPTCIIPVQFSVPNNHLHYHTHAVSPNQCCSAVVQTISAPIHAVWSLVRRFDNPQAYKHFLKSCHVIVGDGNVGTLREVQVISGLPAASSTERLEILDDKEHVISFSVVGGDHRLNNYRSVTTLHPADDENGTVVVESYVVDIPTGNTKEETCVFVDTIVRCNLQSLAQIAtANR

>Solyc10g076410.1.1(SlRCAR7)

MIPNPQKSSILLQRITSNNPCNCNKQSLHHHTPIPCTADIPDSVVKYHAHLVGPYQCCSAAIQRISAPVSTVWSVVRRFDNPQAYKHFIKSCHLIDGDGNVGTLREVRVISGLPAVNSTERLEILDEERHVISFSVVGGDHRLANYKSVTTLHPEPFGNGTIVVESYVVDIPKGNTKDETCVFVDTIVKCNLLSLKQIAENLK

>Solyc09g015380.1.1(SlRCAR8)

MPPKSSLLLQRITTTTTSTSSKQRTPIPCTTQVPDSITSHHTHSVSPNQCCSAVIQRISAPISTVWSVLRRFDNPQAYKHFVKSCHVIGGDGKVGTVREVRVISGLPAANSMERLEILDEERHVISFSVVGGDHRLVNYRSVTTLHSDQSSGTTIVVESYVVDIPHGNTTEETCVFVDTIVKCNLQSLAQIVENSNTTNLK

>Solyc01g095700.2.1(SlRCAR9)

MESQFIERYHSHQPSEHQCSSSLVKHIKAPVDIVWSLVRRFDQPQKYKPFISRCTVKGDLTIGSVREVNVKSGLPAtTSTERLELLDDEEHILGIRIVGGDHRLKNYSSVITVHPETLDGRPGTLVIESFVVDVPEGNTKEETCYFVKALINCNLKSLADVSERMAMQGGVLPVSVNWSSSNQIEI

>Solyc06g050500.2.1(SlRCAR10)

MPSSLQLHRINPTTAtLAVKQPQLSQATTWFSPVSTSVPDNVLHHHTHVVGPNQCCSAVLQSISAPIDTVWSLVRRFDNPQAYKHFLKSCHVIVGDGDVGTLREVRVVSGLPAGSSTERLEILDDEKHVLSFSVVGGDHRLNNYRSVTTLHRAAEEGSTVVVESYVVDVPQGNTKEETCVFVDTIVRCNLQSLAQIAQNLAKTSKNHDEDPQLKIHRLVDFVDCKVGSCKCLNVLLWYIMVCCVFCITTNHFVTITYILLNIFKVLTVKDIFLSS

>Solyc12g055990.1.1(SlRCAR11)

MMNNMEDEYIRRHHRHVLDHNQCSSSLVKRIRAPVNLVWSLVRRFDQPQRYKPFVSRCVVQGDLEIGSVREVNVRSGLPAtTSKERLELLDDEEHIFGVKIVGGDHRLQNYSSIITVHPEVIDGRPGTIVIESFVVDIPDGNTKDETCFFVEALIRCNLKSLADVSERLAVQGHTDPIDRI

>Solyc03g007310.2.1(SlRCAR12)

MNANGFCGVEKEYIRKHHIHEPKENQCSSFLVKHIRAPVHLVWSLVRRFDQPQKYKPFISRCIVQGDLGIGSLREVDVKSGLPAtTSTERLELLDDEEHILSVRIVGGDHRLRNYSSVISVHPEVIDGRPGTLVLESFVVDVPEGNTKDETCYFVEALINCNLKSLADVSERLAVQDRTEPIDQV

>Solyc08g082180.2.1(SlRCAR13)

MVSIMNVDRKISSEDEYIRRHHRHDVRDNQCSSSLVKHIKAPVHLVWSLVRRFDQPQRYKPFVSRCIVQGDLEIGSVREVNVKSGLPAtTSKERLELLDDDEHIFGVKIVGGDHRLRNYSSIITVHPKVIDGRPGTMVVESFVVDVPDGNTKDETCYFVEALIRCNLKSLADVSERLAVQGHMEPIDRM

>Solyc02g076770.1.1(SlRCAR14)

MDQIDIYSNPKCIRPIMLFTNQPKNSKALEEMIKLHHTHELSPKQCSSFLVKVIDAPLPLVWSLVRKFDKPQCYKNFISSCTLISGEGGVGSIREVNLVSGFPGKRSIERLDILDDDMHVSVFSVVDGDHSFSNFKSIMTLHEDKVEEDHDIIGNYYKTVVIHSYVVDIPEISCRDDTCEVTDNILRWNLRSLAWVAENMDTNDQVSSLDLNEKEITC

>Solyc05g052420.1.1(SlRCAR15)

MTSTLQLHRINHHNAAtLAGNFPMTTTTSNICVPDNHFHYHTHPVGPNNNQCCSAVVRSISASIDTVWSIIRRFDNPQAYKNFLKSCHVIVGDGKVVGSLREVHVITGLPAASSIERLEILDDEKKVMSISIIGGDHRLNNYRSVTTLHRAAADDGGDGGRTVVVESYVVDVPQGNTKEETRVFIDTIVRCNLQWLGQIAENLEKTKSN

>AT5G46790.1(AtPYL1)

MANSESSSSPVNEEENSQRISTLHHQTMPSDLTQDEFTQLSQSIAEFHTYQLGNGRCSSLLAQRIHAPPETVWSVVRRFDRPQIYKHFIKSCNVSEDFEMRVGCTRDVNVISGLPANTSRERLDLLDDDRRVTGFSITGGEHRLRNYKSVTTVHRFEKEEEEERIWTVVLESYVVDVPEGNSEEDTRLFADTVIRLNLQKLASITEAMNRNNNNNNSSQVR

>AT2G26040.1(AtPYL2)

MSSSPAVKGLTDEEQKTLEPVIKTYHQFEPDPTTCTSLITQRIHAPASVVWPLIRRFDNPERYKHFVKRCRLISGDGDVGSVREVTVISGLPASTSTERLEFVDDDHRVLSFRVVGGEHRLKNYKSVTSVNEFLNQDSGKVYTVVLESYTVDIPEGNTEEDTKMFVDTVVKLNLQKLGVAATSAPMHDDE

>AT1G73000.1(AtPYL3)

MNLAPIHDPSSSSTTTTSSSTPYGLTKDEFSTLDSIIRTHHTFPRSPNTCTSLIAHRVDAPAHAIWRFVRDFANPNKYKHFIKSCTIRVNGNGIKEIKVGTIREVSVVSGLPASTSVEILEVLDEEKRILSFRVLGGEHRLNNYRSVTSVNEFVVLEKDKKKRVYSVVLESYIVDIPQGNTEEDTRMFVDTVVKSNLQNLAVISTASPT

>AT2G38310.1(AtPYL4)

MLAVHRPSSAVSDGDSVQIPMMIASFQKRFPSLSRDSTAARFHTHEVGPNQCCSAVIQEISAPISTVWSVVRRFDNPQAYKHFLKSCSVIGGDGDNVGSLRQVHVVSGLPAASSTERLDILDDERHVISFSVVGGDHRLSNYRSVTTLHPSPISGTVVVESYVVDVPPGNTKEETCDFVDVIVRCNLQSLAKIAENTAAESKKKMSL

>AT5G05440.1(AtPYL5)

MRSPVQLQHGSDATNGFHTLQPHDQTDGPIKRVCLTRGMHVPEHVAMHHTHDVGPDQCCSSVVQMIHAPPESVWALVRRFDNPKVYKNFIRQCRIVQGDGLHVGDLREVMVVSGLPAVSSTERLEILDEERHVISFSVVGGDHRLKNYRSVTTLHASDDEGTVVVESYIVDVPPGNTEEETLSFVDTIVRCNLQSLARSTNRQ

>AT2G40330.1(AtPYL6)

MPTSIQFQRSSTAAEAANATVRNYPHHHQKQVQKVSLTRGMADVPEHVELSHTHVVGPSQCFSVVVQDVEAPVSTVWSILSRFEHPQAYKHFVKSCHVVIGDGREVGSVREVRVVSGLPAAFSLERLEIMDDDRHVISFSVVGGDHRLMNYKSVTTVHESEEDSDGKKRTRVVESYVVDVPAGNDKEETCSFADTIVRCNLQSLAKLAENTSKFS

>AT4G01026.1(AtPYL7)

MEMIGGDDTDTEMYGALVTAQSLRLRHLHHCRENQCTSVLVKYIQAPVHLVWSLVRRFDQPQKYKPFISRCTVNGDPEIGCLREVNVKSGLPATTSTERLEQLDDEEHILGINIIGGDHRLKNYSSILTVHPEMIDGRSGTMVMESFVVDVPQGNTKDDTCYFVESLIKCNLKSLACVSERLAAQDITNSIATFCNASNGYREKNHTETNL

>AT5G53160.2(AtPYL8)

MEANGIENLTNPNQEREFIRRHHKHELVDNQCSSTLVKHINAPVHIVWSLVRRFDQPQKYKPFISRCVVKGNMEIGTVREVDVKSGLPATRSTERLELLDDNEHILSIRIVGGDHRLKVINESKGSFSVYSESKTYMFYIVFGFRTILQSSLFTPRL

>AT1G01360.1(AtPYL9)

MMDGVEGGTAMYGGLETVQYVRTHHQHLCRENQCTSALVKHIKAPLHLVWSLVRRFDQPQKYKPFVSRCTVIGDPEIGSLREVNVKSGLPATTSTERLELLDDEEHILGIKIIGGDHRLKNYSSILTVHPEIIEGRAGTMVIESFVVDVPQGNTKDETCYFVEALIRCNLKSLADVSERLASQDITQ

>AT4G27920.1(AtPYL10)

MNGDETKKVESEYIKKHHRHELVESQCSSTLVKHIKAPLHLVWSIVRRFDEPQKYKPFISRCVVQGKKLEVGSVREVDLKSGLPATKSTEVLEILDDNEHILGIRIVGGDHRLKNYSSTISLHSETIDGKTGTLAIESFVVDVPEGNTKEETCFFVEALIQCNLNSLADVTERLQAESMEKKI

>AT5G45860.1(AtPYL11)

METSQKYHTCGSTLVQTIDAPLSLVWSILRRFDNPQAYKQFVKTCNLSSGDGGEGSVREVTVVSGLPAEFSRERLDELDDESHVMMISIIGGDHRLVNYRSKTMAFVAADTEEKTVVVESYVVDVPEGNSEEETTSFADTIVGFNLKSLAKLSERVAHLKL

>AT5G45870.1(AtPYL12)

MKTSQEQHVCGSTVVQTINAPLPLVWSILRRFDNPKTFKHFVKTCKLRSGDGGEGSVREVTVVSDLPASFSLERLDELDDESHVMVISIIGGDHRLVNYQSKTTVFVAAEEEKTVVVESYVVDVPEGNTEEETTLFADTIVGCNLRSLAKLSEKMMELT

>AT4G18620.1(AtPYL13)

MESSKQKRCRSSVVETIEAPLPLVWSILRSFDKPQAYQRFVKSCTMRSGGGGGKGGEGKGSVRDVTLVSGFPADFSTERLEELDDESHVMVVSIIGGNHRLVNYKSKTKVVASPEDMAKKTVVVESYVVDVPEGTSEEDTIFFVDNIIRYNLTSLAKLTKKMMK

>AT4G17870.1(AtPYR1)

MPSELTPEERSELKNSIAEFHTYQLDPGSCSSLHAQRIHAPPELVWSIVRRFDKPQTYKHFIKSCSVEQNFEMRVGCTRDVIVISGLPANTSTERLDILDDERRVTGFSIIGGEHRLTNYKSVTTVHRFEKENRIWTVVLESYVVDMPEGNSEDDTRMFADTVVKLNLQKLATVAEAMARNSGDGSGSQVT

>Os02g13330.1(OsPYL/RCAR2)

MEPHMERALREAVASEAERRELEGVVRAHHTFPAAERAAGPGRRPTCTSLVAQRVDAPLAAVWPIVRGFANPQRYKHFIKSCELAAGDGATVGSVREVAVVSGLPASTSTERLEILDDDRHVLSFRVVGGDHRLRNYRSVTSVTEFSSPSSPPRPYCVVVESYVVDVPEGNTEEDTRMFTDTVVKLNLQKLAAVATSSSPPAAGNHH

>Os06g36670.1(OsPYL/RCAR9)

MEAHVERALREGLTEEERAALEPAVMAHHTFPPSTTTATTAAATCTSLVTQRVAAPVRAVWPIVRSFGNPQRYKHFVRTCALAAGDGASVGSVREVTVVSGLPASTSTERLEMLDDDRHIISFRVVGGQHRLRNYRSVTSVTEFQPPAAGPGPAPPYCVVVESYVVDVPDGNTAEDTRMFTDTVVKLNLQMLAAVAEDSSSASRRRD

>Os10g42280.1(OsPYL/RCAR10)

MEQQEEVPPPPAGLGLTAEEYAQVRATVEAHHRYAVGPGQCSSLLAQRIHAPPAAVWAVVRRFDCPQVYKHFIRSCVLRPDPHHDDNGNDLRPGRLREVSVISGLPASTSTERLDLLDDAHRVFGFTITGGEHRLRNYRSVTTVSQLDEICTLVLESYIVDVPDGNTEDDTRLFADTVIRLNLQKLKSVSEANANAAAAAAAPPPPPPAAAE

>Os01g61210.1(OsPYL/RCAR1)

MPYAAVRPSPPPQLSRPIGSGAGGGKACPAVPCEVARYHEHAVGAGQCFSTVVQAIAAPADAVWSVVRRFDRPQAYKKFIKSCRLVDGDGGEVGSVREVRVVSGLPATSSRERLEVLDDDRRVLSFRIVGGEHRLANYRSVTTVHEAAAPAMAVVVESYVVDVPPGNTWEETRVFVDTIVRCNLQSLARTVERLAPEAPRANGSIDHA

>Os05g39580.1(OsPYL/RCAR6)

MMPYTAPRPSPPQHSRIGGCGGGGVLKAAGAAGHAASCVAVPAEVARHHEHAAGVGQCCSAVVQAIAAPVDAVWSVVRRFDRPQAYKHFIRSCRLLDGDGDGGAVAVGSVREVRVVSGLPATSSRERLEILDDERRVLSFRVVGGEHRLSNYRSVTTVHETAAGAAAAVVVESYVVDVPHGNTADETRMFVDTIVRCNLQSLARTAEQLALAAPRAA

>Os03g18600.1(OsPYL/RCAR4)

MPCIPASSPGIPHQHQHQHHRALAGVGMAVGCAAEAAVAAAGVAGTRCGAHDGEVPMEVARHHEHAEPGSGRCCSAVVQHVAAPAAAVWSVVRRFDQPQAYKRFVRSCALLAGDGGVGTLREVRVVSGLPAASSRERLEILDDESHVLSFRVVGGEHRLKNYLSVTTVHPSPSAPTAATVVVESYVVDVPPGNTPEDTRVFVDTIVKCNLQSLAKTAEKLAAGARAAGS

>Os06g33640.1(OsPYL/RCAR7)

MNGAGGAGGAAAGKLPMVSHRRVQCRLADKRCELREEEMEYIRQFHRHEPSSNQCTSFVAKHIKAPLQTVWSLVRRFDQPQLFKPFVRKCVMRENIIVTGCVREVNVQSGLPATRSTERLELLDDNEHILKVKFIGGDHMLKNYSSILTIHSEVIDGQLGTLVVESFVVDIPDGNTKDDICYFIENVLRCNLMTLADVSEERLANP

>Os06g33690.1(OsPYL/RCAR8)

MNGVGGAGGAAAGKLPMVSHRRVQWRLADERCELREEEMEYIRRFHRHEPSSNQCTSFAAKHIKAPLHTVWSLVRRFDQPQLFKPFVRNCVMRENIIATGCIREVNVQSGLPATRSTERLELLDDNEHILKVNFIGGDHMLKNYSSILTVHSEVIDGQLGTLVVESFIVDVPEGNTKDDISYFIENVLRCNLRTLADVSEERLANP

>Os02g15640.1(OsPYL/RCAR3)

MVEVGGGAAEAAAGRRWRLADERCDLRAAETEYVRRFHRHEPRDHQCSSAVAKHIKAPVHLVWSLVRRFDQPQLFKPFVSRCEMKGNIEIGSVREVNVKSGLPATRSTERLELLDDNEHILSVRFVGGDHRLKNYSSILTVHPEVIDGRPGTLVIESFVVDVPEGNTKDETCYFVEALLKCNLKSLAEVSERLVVKDQTEPLDR

>Os05g12260.1(OsPYL/RCAR5)

MVGLVGGGGWRVGDDAAGGGGGGAVAAGAAAAAEAEHMRRLHSHAPGEHQCSSALVKHIKAPVHLVWSLVRSFDQPQRYKPFVSRCVVRGGDLEIGSVREVNVKTGLPATTSTERLELLDDDEHILSVKFVGGDHRLRNYSSIVTVHPESIDGRPGTLVIESFVVDVPDGNTKDETCYFVEAVIKCNLTSLAEVSERLAVQSPTSPLEQ

>29827.m002533(RcPYRL1)

MEKPDASSSPMTDPEQEPTYTSHHLTIPSGLTPSEFEELSPLISEFHTYRINPSQCSSLLAQRVNAPNDVVWSKVRRFDKPQTYKHFIKSCAVEPGFTMTVGSTRDVNVISGLPAATSTERLDILDDDRHVTGFTIIGGE

HRLRNYRSVTTVHGFQRDGRIWTVVLESYVVDVPEGNTEEDTRLFADTVVKLNLQKLAFVAEGLARDADGKSQVI

>29794.m003335(RcPYRL2)

MEPSRSPPPQGLTREEYSELKPLVDTYHKFEPTPNTCTSLITQRIDAPSQVVWPFVRSFENPQKYKHFIKSCNMRGDGGIGSIREVTVVSGIPASTSTERLEILDDEKHILSFRVVGGEHRLNNYRSVTSVNEFINNEGK

VYTIVLESYIVDIPEGNTGEDTKMFVDTVVKLNLQKLAVVATASLHGHE

>29820.m001002(RcPYRL3)

MPKSTLLLHRINTATTTNSTTTTSTCQKRWCPLSCEPTVPGTVSRYHNHVLRPNQCCSAVVQQIAAPVSTVWSVVRRFDNPQAYKHFVKSCHLINGDGDVGTLREVHVISGLPAANSTERLDILDDERHVISFSVVGGDH

RLANYKSITTLHPSPSGNGTVVVESYVVDVPPGNTKEDTCVFVDTIVRCNLHSLAQIAQNLARLNKS

>29729.m002290(RcPYRL4)

MPAASLQLQIPNTATTTTTTTSAALSCYKHSWQPPVPLSWDAAVPDYVSCYHTRSVGPDQCCSAVFKIINAPVSTVWSVVRRFDNPQAYKHFVKSCHLINGDGDVGTLREVHVVSGLPAESSTERLEILDDEQHVISFSM

IGGDHRLKNYRSVTTLHASPNGNGTVVIESYVVDIPAGNTEEETCVFVDTILRCNLQSLAQIAENMAKN

>29742.m001442(RcPYRL5)

MNCGDVYSVVESQYIRRHHRHQPMENQCTSALVKHIKAPVHLVWSLVRRFDQPQKYKPFVSRCVVSGELGIGSVREVNVKSGLPATTSTEMLELLDDEEHILGIKIVGGDHRLKNYSSIMTVHPEVIDGRPGTLVIESFV

VDVPDGNTKDETCYFVKALIKCNLKSLADVSERMAVQDCVEPINRF

>30169.m006525(RcPYRL6)

MSGRSNGNVAIGNGIIGSVESEYVRRHHRHDPADHQCSSALVKHIKAPVHLVWSLVRRFDQPQKYKPFISRCVAQGNLQIGSLREIDVKSGLPATTSTERLEFLDDDEHILSMRIVGGDHRLKNYSSIISLHPEIIDGRP

GTLVIESFVVDVPDGNTKDETCYFVEALIKCNLKSLADVSERLAVQDRTEPIDCI

>30190.m010824(RcPYRL7)

MVAGDYITVLNTNNNIFSKMEDDYIRRHHKHDVKDHQCSSSLVKHIKAPVHLVWSLVRRFDQPQRYKPFVSRCIAQGDLQIGSLREVNVKSGLPATTSTERLELLDDEEHIFRMTIVGGDHRLKNYSSIITVHPEVIDGR

PGTMAIESFVVDVPDGNTKDETCYFVEALIKCNLTSLANVSEHLAVHDRTEPIDRI

>Glyma01g02290.1(GmPYRL1)

MEKAESSASTSEPDSDENHHRHPTNHHINPPSGLTPLEFASLIPSVAEHHSYLVGSGQCSSLLAQRVQAPPDAVWSVVRRFDKPQTYKHFIKSCAVKEPFHMAVGVTRDVNVISGLPAATSTERLDLLDDIRCVTGFSII

GGEHRLRNYRSVTTVHSFEDDADDGKIYTVVLESYVVDVPDGNTEEDTRLFADTVVKLNLQKLASVTEGTNRDGDGKSHSR

>Glyma09g33700.1(GmPYRL2)

MEKAESSASTSEPDSDDNHHRHPTNHHLNPPSGLTPLEFASLVPSVAEHHSYLVGPGQCSSLLAQRVHAPPDAVWSFVRRFDKPQTYKHFIKSCAVKEPFHMAVGVTRDVNVISGLPAATSTERLDFLDDVRRVTGFSII

GGEHRLRNYRSVTTVHSFDDDNASADGKIYTVVLESYVVDVPDGNTEEDTRLFADTVVKLNLQKLASVTEGTNGDGDGKPHSR

>Glyma01g12970.1(GmPYRL3)

MEKTHSSSAEEQDPTRRHLDPPPGLTAEEFEDLKPSVLEHHTYSVTPTRQSSSLLAQRIHAPPHAVWSVVRCFDNPQAYKHFIKSCHVKEGFQLAVGSTRDVHVISGLPAATSTERLDLLDDDRHVIGFTIVGGDHRLRN

YRSVTSVHGFECDGKIWTVVLESYVVDVPEGNTEEDTRLFADTVVKLNLQKLASVSEGMCGDGDGDGDGKGNKS

>Glyma08g36770.1(GmPYRL4)

MSRSHNKRKPFSFIFKITLLELLSSLLSSSLRFAMDKTHSGEEQDPNPTHPTRNHLDPPPGLTPEEFEDLKPSVLEHHTYSVTPTRQCSSLLAQRIHAPPHTVWTVVRCFDNPQAYKHFIKSCHVKEGFQLAVGSTRDVH

VISGLPAATSTERLDLLDDDRHVIGFTIVGGDHRLRNYRSVTSVHGFERDGKIWTVVLESYVVDVPEGNTEEDTRLFADTVVKLNLQKLASVTEGMCGDSDGKGNN

>Glyma14g06100.1(GmPYRL5)

MVARHHAHAVGPNQCCSFVIQAIDAPVSAVWPVVRRFDNPQAYKHFVKSCHVVAAGGAGGDGGIHVGALREVRVVSGLPAVSSTERLEILDDERHVMSFSVVGGDHRLRNYRSVTTLHGDGSNGGTVVIESYVVDIPAGN

TKEETCVFVDTIVRCNLQSLAQMAENMGS

>Glyma06g05440.1(GmPYRL6)

EFTELESTINTHHKFEASPEICSSIIAQRIDAPAHTVWPLVRSFENPQKYKHFVKSCNMRSGDGGVGSIREVTVVSGLPASTSTERLEILDDDNHLLSFRVVGGEHRLHNYRSVTSVNEFKRPDNGKVYTIVLESYVVDI

PEGNTGVDTKMFVDTVVKLNLQKLGEVAMATN

>Glyma04g05380.1(GmPYRL7)

AYPVLGLTPEEFSELESIINTHHKFEPSPEICSSIIAQRIDAPAHTVWPLVRSFENPQKYKHFVKSCNMRSGDGGVGSIREVTVVSGLPASTSTERLEILDDDKHLLSFRVVGGEHRLHNYRSVTSVNEFKNPDNGKVYT

IVLESYVVDIPEGNTGVDTKMFVDTVVKLNLQKLGE

>Glyma17g34800.1(GmPYRL8)

MASETHHHVQGLTPEELTQLEPIIKKYHLFEASSNKCFSIITHRIEAPASSVWPLVRNFDNPQKYKHFIKGCNMKGDGSVGSIREVTVVSGLPASTSTERLEILDDDKHVLSFRVVGGEHRLQNYRSVTSVNEFHKEGKV

YTIVLESYIVDIPEGNTEEDTKMFVDTVVKLNLQKLGVVAMASSMNGR

>Glyma02g42990.1(GmPYRL9)

MTSLQFHRFNPATDTSTAIANGVNCPKPPSTLRLLAKVSLSVPETVARHHAHPVGPNQCCSVVIQAIDAPVSAVWPVVRRFDNPQAYKHFVKSCHVVAAAGGGEDGIRVGALREVRVVSGLPAVSSTERLEILDDERHVM

SFSVVGGDHRLRNYRSVTTLHGDGNGGTVVIESYVVDVPPGNTKEETCVFVDTIVRCNLQSLAQIAET

>Glyma18g43680.1(GmPYRL10)

MTSLQFHRFNPATDTSTAIANGVNCPKPPSTLRLLAKVSLSVPETVARHHAHPVGPNQCCSVVIQAIDAPVSAVWPVVRRFDNPQAYKHFVKSCHVVAAAGGGEDGIRVGALREVRVVSGLPAVSSTERLEILDDERHVM

SFSVVGGDHRLRNYRSVTTLHGDGNGGTVVIESYVVDVPPGNTKEETCVFVDTIVRCNLQSLAQIAET

>Glyma14g10730.1(GmPYRL11)

MTILPHSNNKSSNHKFIAHQNYMASETHHHVQGLTPEELTKLEPIIKKYHLFEQSPNTCFSIITYRIEAPAKAVWPFVRSFDNPQKYKHFIKGCNMRGDGGVGSIREVTVVSGLPASTSTERLEILDDDKHVLSFRVVGG

EHRLKNYRSVTSVNEFNKEGKVYTIVLESYIVDIPEGNTEEDTKMFVDTVVKLNLQKLGVVAMASSMHGQ

>Glyma07g19120.1(GmPYRL12)

MSPNNPSTIVSDAVARHHTHVVSPHQCCSAVVQEIAAPVSTVWSVVRRFDNPQAYKHFVKSCHVILGDGDVGTLREVRVISGLPAAVSTERLDVLDDERHVIGFSMVGGDHRLSNYRSVTILHPRSATDTVVVESYVVDV

PAGNTTEDTRVFVDTILRCNLQSLAKFAENLTNKLHQR

>Glyma01g31320.1(GmPYRL13)

MLQNSSMSSLLLHRINGGGGATTATNCHDTVFMTVPDGVARYHTHAVAPNQCCSSVAQEIGASVATVWSVLRRFDNPQAYKHFVKSCHVIGGDGDVGTLREVHVISGLPAARSTERLEILDDERHVISFSVVGGDHRLAN

YRSVTTLHPTASSASGGCSGTVVVESYVVDVPPGNTREDTRVFVDTIVKCNLQSLAQTAENLTLRKNNNNDYKCCS

>Glyma11g35670.1(GmPYRL14)

MPSSLHFDRFNPITHAATTVAIANGVNCPKQPQAPPSSTAARRLVVPSLSSGRGIAAPDTVALHHAHVVDPNQCCSIVTQHINAPVSAVWAVVRRFDNPQGYKNFVRSCHVITGDGIRVGAVREVRVVSGLPAETSTERL

EILDDERHVISFSMVGGDHRLRNYQSVTTLHANGNGTLVIESYVVDVPQGNTKEETCVFVDTIVRCNLQSLAQIAENRTNNCEHTAQHC

>Glyma07g06270.1(GmPYRL15)

MNGGESYGAIETQYIRRHHKHEPRENQCTSALVKHIRAPVHLVWSLVRRFDQPQKYKPFVSRCIMQGDLGIGSVREVNVKSGLPATTSTERLEQLDDEEHILGIRIVGGDHRLRNYSSIITVHPEVIDGRPGTMVIESFV

VDVPDGNTRDETCYFVEALIRCNLSSLADVSERMAVQGRTNPINH

>Glyma16g02910.1(GmPYRL16)

MGITIGIQCLEIEEISICDGMFCYLVDFVDVKEKMNYCLMWFGYFPSQVWSLVRRFDQPQKYKPFVSRCIMQGDLGIGSVREVNVKSGLPATTSTERLEQLDDEEHILGIRIVGGDHRLRNYSSIITVHPEVIDGRPSTM

VIESFVVDVPDGNTRDETCYFVEALIRCNLSSLADVSERMAVQGRTDPINH

>Glyma14g30260.1(GmPYRL17)

MNRIGNGGGGGGGLSNVEMEYIRRHHRHEPGENQCGSALVKHIRAPVPQVWSLVRRFDQPQKYKPFISRCVVRGNLEIGSLREVDVKSGLPATTSTERLELLDDNEHILSIRIIGGDHRLRNYSSIMSLHPEIIDGRPGT

LVIESFVVDVPEGNTKDETCYFVEALIKCNLKSLADVSEGLAVQDCTEPIDRI

>Glyma13g08120.1(GmPYRL18)

MNGIGNDGGGGLSNVEMEYIRRHHRHEPGENQCGSALVKHIRAPVPQVWSLVRRFDQPQKYKPFVSRCVVRGNLEIGSLREVDVKSGLPATTSTERLELLDDNEHLLSIRIIGGDHRLRNYSSIMSLHPEIIDGRPGTLV

IESFVVDVPEGNTKDETCYFVEALIKCNLKSLADVSEGIAVQDRTEPIDRI

>Glyma15g08930.1(GmPYRL19)

MLCKQNLETPTIKAMLNTYHASKLPSNQCGSSLVQTIDAPLPLVWSLIRRFEYPQGYKLFVKKCTLLDGNGGIGSVREVMVTSGLPAGVSVERLDKLDDDKHVFKFSIIGGDHRLVNYSSTITLHQEEEEYGGKTVAIES

YAVDVPAGSTVDDTCSFANTIIACNLRSLAKITEEMVCKANQIKV

>Glyma13g30210.1(GmPYRL20)

MLCKQDLETPTIKAMLNTYHASKLSSNQCGSSLVQTIDAPLPLVWSLIRRFEYPQGYKLFVKKCNLLDGDGGIGSVREVMITSGLPAGVSVERLDKLDDDKHVLKFSIIGGDHRLVNYSSTITLHEEEEEYGGKTVAIES

YAVDVPAGSSGDDTCSFANTIIACNLRSLAKITEEKVCKVNQIKL

>Glyma06g13150.1(GmPYRL21)

MTELSSREVEYIRRHHSKAAEDNQCASALVKHIRAPLPLVWSLVRRFDEPQKYKPFVSRCVVRGNLEIGSLREVDVKSGLPATTSTERLEILDDNHHILSVRIIGGDHRLRNYSSIMSLHPEIVDGRPGTLVIESFVVDI

PEGNTKDETCYFVEALIKCNLKSLADVSEGLTLQDHTEPIDRKYELLITRG

>Bradi3g34070.1(BdPYRL1)

MEPQQQPDAAAAAGAGAGEPEVPAGLGLTAAEYAQLRPTVEAYHLYAVGQGQCSSLLAQRIEAPAAAVWAIVRRFDCPQVYKHFIRNCALRPDPNAGAGEDDGELRPGRLREVSVISGLPASTSTERLDLLDDARRAFGF

TIIGGEHRLRNYRSVTTVSEIRAAGAAAVVLESYIVDVPEGNSEEDTRLFADTVVRLNLQKLKSVAEANAASNAPAPPPAE

>Bradi1g37810.1(BdPYRL2)

MEAHMERALREGLTEAERASLEGAVRAHHTFPGRAATCTSLVAQRVAAPVRDVWPIVRSFGNPQRYKHFVRTCALAAGDGASVGSVREVTVVSGLPASTSTERLEILDDDRHILSFSVVGGEHRLRNYRSVTSVTEFQGQ

EDAGAPPYCVVLESYVVDVPPGNTEDDTRMFTDTVVKLNLQKLASVAEESGSRTRD

>Bradi3g08580.1(BdPYRL3)

MEPHMERALREALTEAERRSLAPVVAAHHTFPGSGQSSPSPSPGKKKTCTSLVTQRVDAPLAAVWAIVRGFATPQRYKHFIKSCALAAGDGATVGSVREVTVVSGLPASTSTERLEILDDDRHVLSFRVVGGEHRLRNYR

SVTSVTEFSSPPPPEPEEKEKEETQDAAASSSYCVVVESYVVDVPEGNTEEDTRMFTDTVVKLNLQKLAAIATTSTSSPSASPSSPPPPQPSDGAQADH

>Bradi2g22510.1(BdPYRL4)

MPYTASRSRPSPAQRSRVGGGRKGAAAAVPEEVARHHEHAAGAGQCCSAVVQESIAAPVEAVWAVVRRFDRPQAYKHFIRSCRLVDGDGGAVGSVREVRVVSGLPATSSRERLEILDDERRVLSFRVVGGEHRLSNYRSV

TTVHHAETTGSTVVVESYVVDVPAGNTADETRTFVDTIVRCNLQSLARTAEQLAAAD

>Bradi1g16710.1(BdPYRL5)

MPAPYSAAAAQQLQQHRPLAAAVTGSRCGEHDGTVPAEVAQHHSHPPSSSAGPWRCCSAVVQRVRAPTSAVWSVVRRFGEPQAYKSFVRSCAVVDGDGGVGTLREVRVVSGLPAASSRERLEVLDDDRRVLSFRVVGGEH

RLRNYRSVTTVHPSSSSSSPAEEETVVVESYVVEVPAGNTAEDTRTFVDTIVKCNLLSLARTAEKLSAAGRCP

>Bradi1g65130.1(BdPYRL6)

MPCIPASSPSSIHHHHHQRHRVLAMGCGAELAAVQGASGMARCGAHDGEVPAEVSRHHEHAAADPAGSGMRCCSAVVQHVAAPAADVWSVVRRFDQPQAYKRFVRSCALVAGDGGVGTLREVRVVSGLPAASSRERLEVL

DDESHVLSFRVVGGEHRLKDYLSVTTVHPSPAAPSSATVVVESYVVDVPPGNTVEDTRVFIDTIVKCNLQSLAKTAEKLAAGGRAVS

>Bradi2g53840.1(BdPYRL7)

MPYTATRPSPPQHSRTVGAARNKAPLAVPLPAEVARYHEHAAGAGQCGSAVVQAIGAPAEAVWAVVRRFDRPQAYKRFVKSCRLVEDGGSVGVGSVREVRVVSGLPATCSRERLEVLDDERRVLSFRIVGGEHRLANYRS

VTTVSEVPVAGGAGKPVSVVVESYVVDVPPGNTGDETRVFVDTIVRCNLLSLARAAEAEAQLALAPVQSPRVS

>Bradi2g32250.1(BdPYRL8)

MVGLVDGSARGWRLSDEAATSGAGRGGGGGGVTAAADHMRRLHSHALGEHQCSSTLLKHIKAPVHLVWSLVRSFDQPQRYKPFVSRCVVRGGDLEIGSLREVNVKTGLPATTSTERLEQLDDEEHILSVKFVGGDHRLRV

QLLIYHNCPPGEH

>Bradi3g09580.1(BdPYRL9)

MVEIDGAVGVGGGAGGVEGARRWRLADERCDLRATESDYVRRFHPHEPRDHQCSSAVAKHIKAPVHLVWSLVRRFDQPQLFKPFVSRCEMKGNIEIGSVREVNVKSGLPATRSTERLELLDDTEHILSVKFVGGDHRLKN

YSSILTVHPEVIDGRPGTLVIESFVVDVPEGNTKDETCYFVEALIKCNLKSLAEVSERLVVKDQTEPLDR

>POPTR_0001s02490.1(PtPYRL1)

MTDPAQQEPTTYTTHHVTIPPSLTQSEFDELNPLITEFHNYRIRPGQCSSLLAQRINAPNDLVWSLARRFDKPQTYKHFIKSCSVAPGFTMTVGSTRDVNVISGLPAATSTERLDILDDERQVTGFSIIGGEHRLKNYRS

VTTVHGFEREGKIWTVVLESYVVDVPEGNTEEDTRLFADTVVKLNLQKLASVAEGLARDGDGK

>POPTR_0003s09050.1(PtPYRL2)

MTDPEQQESIASTTHHITIPSGLTQSESEELAPLITEFHTYRISAGQCSSLLAQLISAPNDTVWSIVRRFDKPQTYKHFIKSCSVGPGFTMTVGSTRDVNVISGLPAATSTERLDILDDEQQLTGFSIIGGEHRLRNYRS

VTTVHGFEREGKIRTVVLESYVVDVPEGNTEEEARLFADTVVKLNLQKLASVAESLVRDGDGK

>POPTR_0018s05090.1(PtPYRL3)

MDTNQAPPPQGLTQEEYMELKPLIDTYHKFEPAPNTCTSLITQRIDAPAHVVWPFVRRFDNPQKYKHFIKSCNMSAGDGGVGSVREVAVVSGLPASTSIERLEILDDENHILSFRVVGGEHRLNNYKSVTSVNEFNKEGK

VYAIVLESYIVDIPGGNTGEDTKMFVDTVVKLNLQKLAVVAIASLHGHE

>POPTR_0006s24670.1(PtPYRL4)

MDANHAPPVPQGLTQEEYVELKPLIDTYHKFGAAVPNTCTSLITQRIDAPAHVVWPFVRRFDNPQKYKHFIKSCKMSAGDGGVGSIREVTVVSGIPASTSTERLEILDDENHILSFRVVGGEHRLNNYKSVTSVNEFNKE

DKVYTIVLESYIVDIPDGNTVEDTEMFVDTVVKLNLQKLAVVANTALHGHE

>POPTR_0010s19120.1(PtPYRL5)

MPASLQLQRAAIPTTTTTLACHKQSQTTVNTWRVPLTWDAPVPDYVSCHHTRLVGPKQCCSVVVKTINAPVSTVWSVVRRFDNPQAYKHFVKSCHVIDGDGNVGSLREVHVVSGLPAASSTERLEILDDEQHVLSFSVVG

GVHRLNNYRSVTTLHASPNGNGTVVVESYVVDVPAGNTKEDTCSFIETIVRCNLQSLAQIAEKMARNAQISTSS

>POPTR_0006s10500.1(PtPYRL6)

MPANPPKSSLLIHRINNTTITNTTLNTTNTTTSTSCQKRWSPLTCATIPVPETVSRYHAHAVGPNQCCSAVVQQIAAPVSTVWSVVRHFDNPQAYKHFVKSCHVILGDGDVGTLREVHVISGLPAAKSTERLEILDHERH

VISFSVVGGDHRLANYRSVTTLHASPTGNGTVVVESYVVDIPPGNTKEDTCVFVDTIVRCNLQSLAQIAENKNRRNNKSSS

>POPTR_0008s07340.1(PtPYRL7)

MYLSLTDHRKKKAKRFKMPASLQLQRAAATSTTTTRTGHKQSQTTVNTWGVPLPWDTPVPDYVSCHHTRIPGPNQCCSVVVQTINAPVATVWSVVRRFDNPQAYKHFLKSCHVIDGDGKVGSLREVHVVSGLPAASSTER

LEILDDEQHILSFSVVGGVHRLNNYRSVTTLHASPNGNGTVVVESYVVDVPTGNTKEDTCSFLDTIVRCNLQSLAQIAGKKARNNQISITS

>POPTR_0016s13320.1(PtPYRL8)

MPANPPRSSLLIHRINNTTSNTTLNTTNTTTATSCQKRWSPLPCDATPVPETVSRYHTHAVGPNQCCSAVVQQIAAPISTVWSVVRRFDNPQAYKHFVKSCHVILGDGDVGTLREIHVISGLPAAHSTERLEILDDERHV

ISFSVVGGDHRLANYKSVTTLHSSPSGNGTVVMESYAVDIPPGNTKEDTCVFVDTIVRCNLQSLAQIAENSNRRNNKSSSA

>POPTR_0002s17070.1(PtPYRL9)

MNGGDAYSAAEVQYIRRHHQHEPAENQCTSALVKHIKAPAHLVWSLVRRFDQPQRYKPFVSRCVMNGELGIGSVREVNVKSGLPATTSTERLELLDDEEHILGVKIVGGDHRLKNYSSIMTVHPEIIDGRPGTLVIESFI

VDVPDGNTKDETCYFVKALIRCNLKSLADVSERMAVQDLVEPINQF

>POPTR_0014s09280.1(PtPYRL10)

MNGSDAYSATEAQYVRRHHKHEPRENQCTSALVKHIKAPAHLVWSLVRRFDQPQRYKPFVSRCVMNGELGIGSVREVNVKSGLPATTSTERLELLDDEEHILGVQIVGGDHRLKNYSSIMTVHPEFIDGRPGTLVIESFI

VDVPDGNTKDETCYFVKALIRCNLKSLADVSERMAVQDRVEPVNQF

>POPTR_0015s02210.1(PtPYRL11)

MNGNCNGRGGIGCVESEYIRRHHTHDDLADHQCSSALVKHIKAPVQLVWSLVRRFDQPQKYKPFISRCVVLGNLEIGSLREVDVRSGLPATTSTERLELLDDDEHILSIRIVGGDHRLKNYSSIISLHPEIIDGRPGTLV

IESFVVDVPDGNTKDETCYFVEALIKCNLKSLADVSEHLAVQDRTEPIDCM

>POPTR_0003s13900.1(PtPYRL12)

MVTNDYVTIASGKMEDEFIKRHHKHDVKEHQCSSSLVKHIKAPVPLVWSLVRRFDQPQKYKPFVSRCVVQGDLQIGSVREVNVKSGLPATTSTERLELLDDEEHIFSMKIVGGDHRLKNYLSTVTVHPEVIDGRPGTLVI

ESFIVDVPDGNTKDETCYFVEALIKCNLKSLADVSERLAVQDRTEPIDRM

>POPTR_0012s01550.1(PtPYRL13)

MNENSNGRGGIGSVESEYIRRHHKHGDLADHQCSSALVKHIKAPVHLVWSLVRRFDQPQKYKPFISRCVVLGNLEIGSLREVDVRSGLPATTSTERLELLDDDEHIFSIRIVGGDHRLKNYSSVISLHPEIIDGRPGTLV

IESFVVDVPDGNTKDETCYFVEALIKCNLKSLADVSESHAVQDRTEPIECM

>POPTR_0001s10530.1(PtPYRL14)

MVTNDYVTIANGMMEDEFIKRHHKHDVKEHQCSSSLVKHIKAPVPLVWSLVRRFDQPQKYKPFVSRCIAQGDLQIGSVREVNVKSGLPATTSTERLELLDDDEHIFGMKIVGGDHRLKNYSSIVTVHPKVIDERPGTLVI

ESFVVDVPDGNTKDETCYFVEALIKCNLKSLADVSERLAVQGRTEPIDRT

>GSVIVT01013161001(VvPYRL1)

MEEIEKYKTSSHFSSPASLFAAMNKAETSSMAEAESEDSETTTPTTHHLTIPPGLTQPEFQELAHSISEFHTYQVGPGQCSSLLAQRVHAPLPTVWSVVRRFDKPQTYKHFIKSCHVEDGFEMRVGCLRDVNVISGLPAETSTERLDILDDERHVTGFSI

IGGEHRLRNYRSVTTVHEYQNHGGEIWTVVLESYVVDMPEGNTEEDTRLFADTVVKLNLQKLASVTEGMARDQGCFYPLRCMISRVCSMSLIKMITLDGILSSYFKLSEREADFHVGCPTSCFIYA

>GSVIVT01035362001(VvPYRL2)

MDPHHHHGLTEEEFRALEPIIQNYHTFEPSPNTCTSLITQKIDAPAQVVWPFVRSFENPQKYKHFIKDCTMRGDGGVGSIREVTVVSGLPASTSTERLEILDDEKHILSFRVVGGEHRLNNYRSVTSVNDFSKEGKDYTIVLESYIVDIPEGNTGEDTKM

FVDTVVKLNLQKLAVVAITSLHENE

>GSVIVT01019517001(VvPYRL3)

MSGYGCIKMEDEYIRRHHRHEIRDNQCSSSLVKHIKAPVHLVWSLVRSFDQPQKYKPFVSRCIVQGDLEIGSVREVNVKSGLPATTSTERLELLDDEEHIFGMRIVGGDHRLKNYSSIVTVHPEIIDGRPGTLVIESFVVDVPDGNTKDETCYFVEALIK

CNLKSLADVSERLAIQDRTEPIDRM

>GSVIVT01028704001(VvPYRL4)

MNGNGLSSMESEYIRRHHRHEPAENQCSSALVKHIKAPVPLVWSLVRRFDQPQKYKPFISRCVVQGNLEIGSLREVDVKSGLPATTSTERLELLDDDEHILSMRIIGGDHRLRNYSSIISLHPEIIDGRPGTMVIESYVVDVPEGNTKDETCYFVEALIK

CNLKSLADVSERLAVQDRTEPIDRM

>GSVIVT01027078001(VvPYRL5)

MMEAQVICRHHAHEPRENQCSSVLVRHVKAPANLVWSLVRRFDQPQKYKPFVSRCVVQGDLRIGSVREVNVKTGLPATTSTERLELFDDDEHVLGIKILDGDHRLRNYSSVITVHPEIIDGRPGTLVIESFVVDVPEGNTKDDTCYFVRALINCNLKCLA

EVSERMAMLGRVEPANAV

>Pp1s231_41V6.1(PpPYRL1)

MQTKGRQADFQTLLEGQQDLICRFHRHELQPHQCGSILLQLIKAPVETVWSVARSFDKPQVYKRFIQTCEIIEGDGGVGSIREVRLVSSIPATSSIERLEILDDEEHIISFRVLGGGHRLQNYWSVTSLHSHEIDGQMGTLVLESYVVDIPEGNTREETHMFVDTVVRCNLKALAQVSEHRFFHELRAKELSAASLSLGSKEEAVHARKKEAVQV

>Pp1s52_236V6.1(PpPYRL2)

MMQEKQGRPDFQFLLEGQQDLICRFHKHELLPHQCGSILLQQIKAPVQTVWLIVRRFDEPQVYKRFIQRCDIVEGDGVVGSIREVQLVSSIPATSSIERLEILDDEEHIISFRVLGGGHRLQNYWSVTSLHRHEIQGQMGTLVLESYVVDIPDGNTREETHTFVDTVVRCNLKALAQVSEQKHLLNSNEKPAAP-

>Pp1s97_204V6.1(PpPYRL3)

MVRSDVEGGRGAVASEDVVGSGSDSVCTSTASQPPPASSSSQGSGVKSAPSSVGSVSVRGVGAESSQPQSKTTTTTTTTTTTTTTHPMGALGMMQQVKGRQDFQRLLEAQQDLICRYHTHELKAHQCGSILLQQIKVPLPIVWAIVRSFDKPQVYKRFIQTCKITEGDGGVGSIREVHLVSSVPATCSIERLEILDDEKHIISFRVLGGGHRLQNYSSVSSLHELEVEGHPCTLVLESYMVDIPDGNTREETHMFVDTVVRCNLKSLAQISEQQYNKDCLQQKQHDQQQMYQQRHPPLPPIPITDKNMESSPQDREAVLKQEVLNLNQR

>Pp1s89_26V6.1(PpPYRL4)

MRFDIGHNDVRGFFTCEEEHAYALHSQTVELNQCGSILMQQIHAPIEVVWSIVRSFGSPQIYKKFIQACILTVGDGGVGSIREVFLVSGVPATSSIERLEILDDEKHVFSFRVLKGGHRLQNYRSVTTLHEQESGGPCKGLRNNCQPPPCKGLDLSCWGYGLCVHQRCSARLLAVFFFFFFPTSFAARSLTTLAFSCLRSITLQRLAHGTAATATVTFLKTLLACSQRPKPVLPAFGQLFRLPL
