# Supplementary material for: Co-silencing of ABA receptors (SlRCAR) reveals interactions between ABA and ethylene signaling during tomato fruit ripening
Source: Hortic Res. 2022 Jun 5;9:uhac057. doi: 10.1093/hr/uhac057 (PMC9171117; doi:10.1093/hr/uhac057)
Supplement: Web_Material_uhac057 [file web_material_uhac057.zip › Supplementary material.docx]

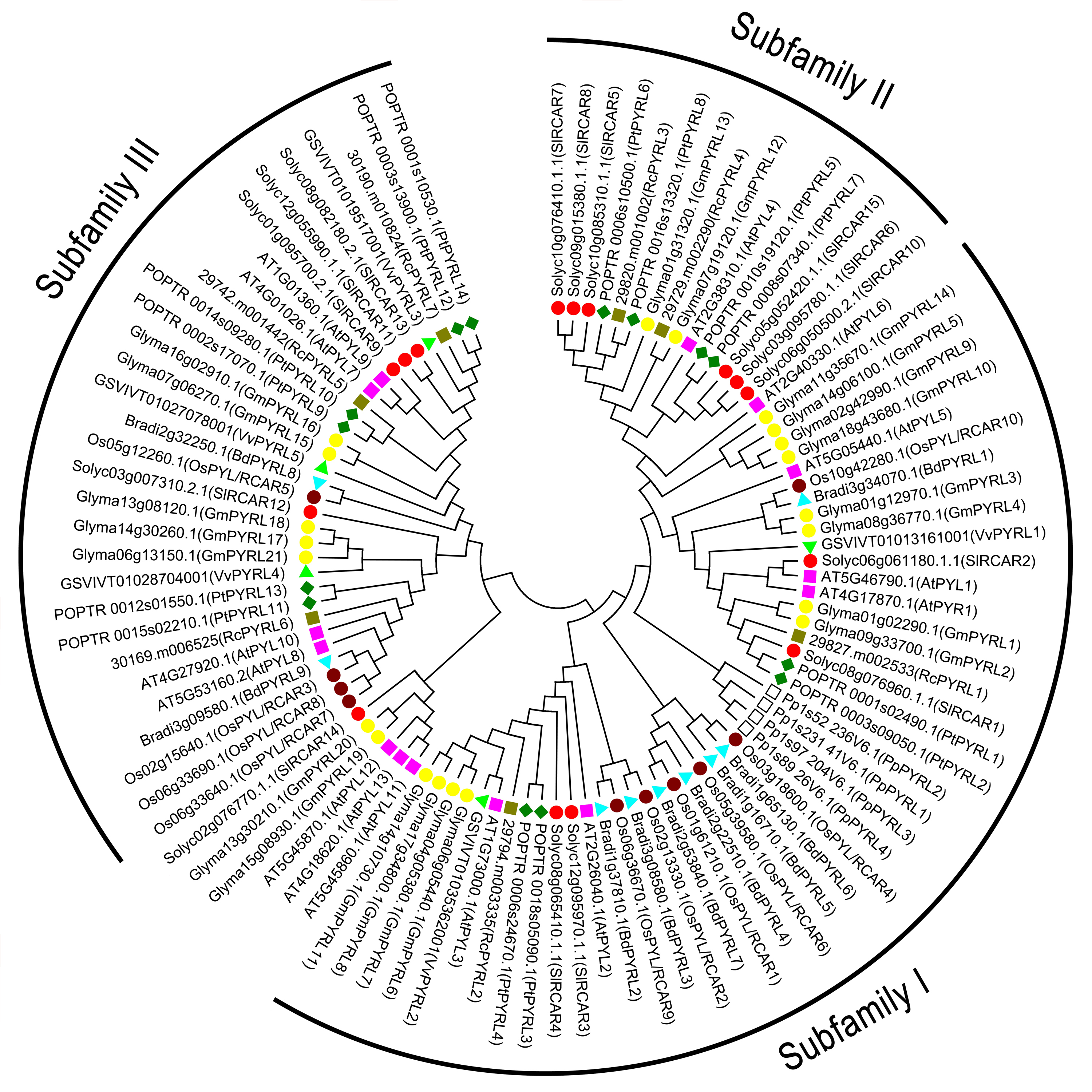


**Supplementary Figure S1 Phylogenetic tree of PYR/PYL ABA receptors.** Phylogenetic analysis of ABA receptor (PYR/PYL/RCAR) family members in tomato, together with those from *Arabidopsis thaliana*, *Ricinus communis*, *Glycine max*, *Brachypodium distachyon*, *Populus trichocarpa*, *Medicago truncatula*, *Vitis vinifera*, and *Physcomitrella patens*, using the neighbor-joining method on MEGA6.


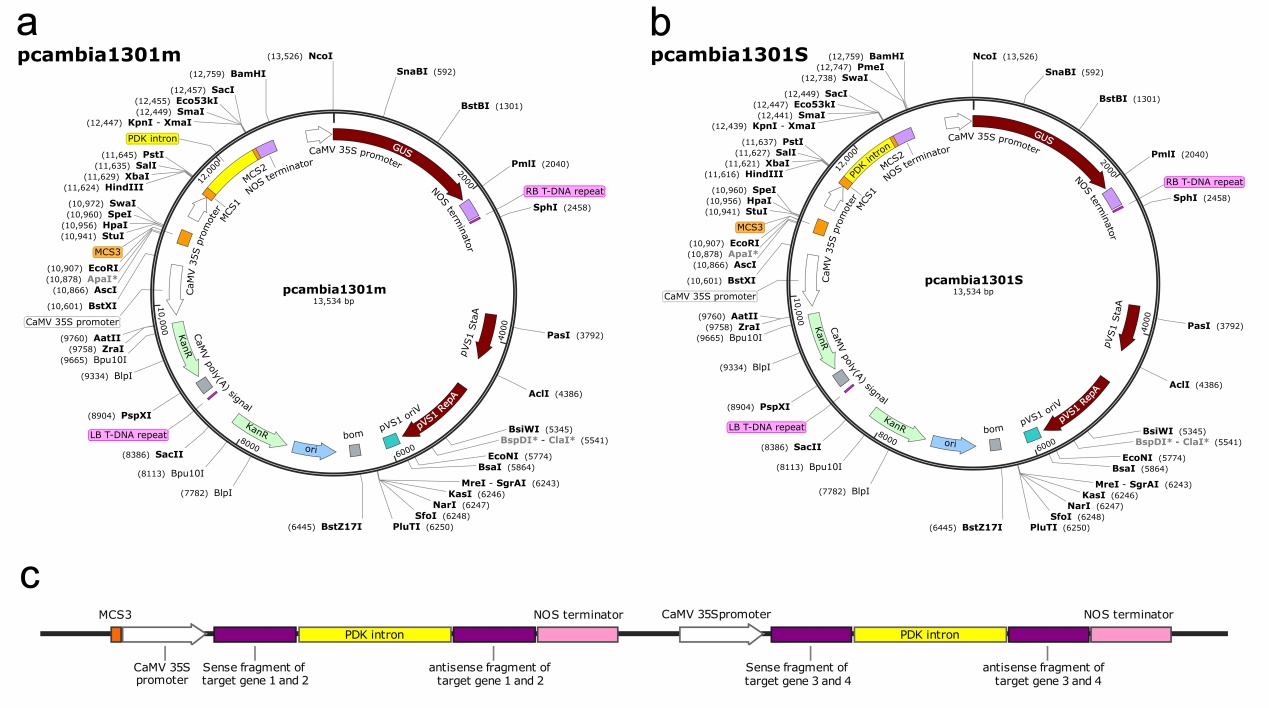


**Supplementary Figure S2 Map of multiple-gene silencing system.** **(a)** Multiple-gene RNAi vector system pCAMBIA1301m. **(b)** Vector system pCAMBIA1301s. **(c)** Schematic diagram of *SlRCARs* multi-gene interference.


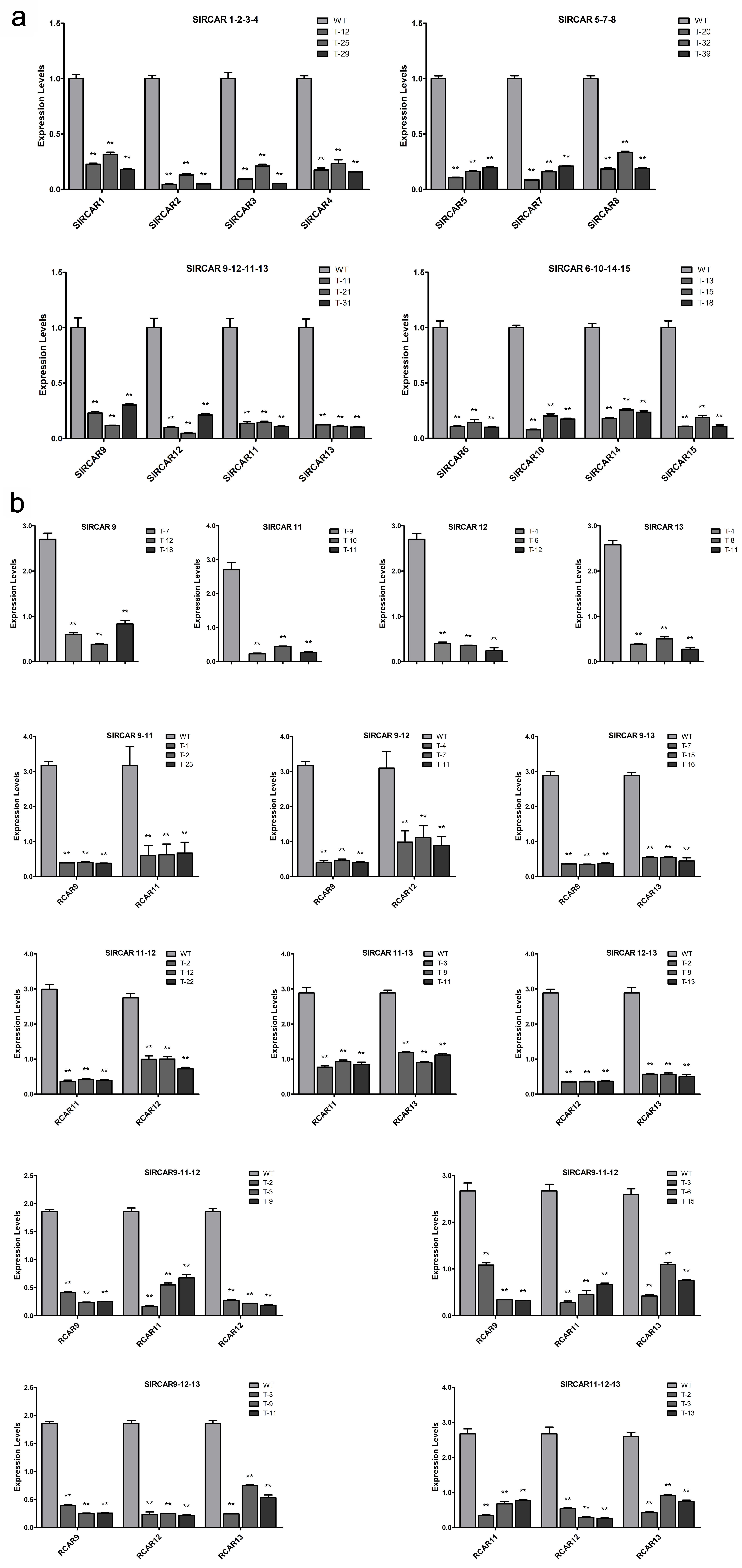


**Supplementary Figure S3 Repression efficiency detection of ABA receptor genes in transgenic tomato. (a)** Relative expression levels of target genes in multiple-gene silenced transgenic tomato, including SlRCAR1-2-3-4, SlRCAR5-7-8, SlRCAR6-10-14-15, and SlRCAR9-12-11-13. **(b)** Relative expression levels of target genes (including *SlRCAR9*, *SlRCAR12*, *SlRCAR11*, and *SlRCAR13*) in single-gene, double-gene, and three-gene silenced transgenic tomato.


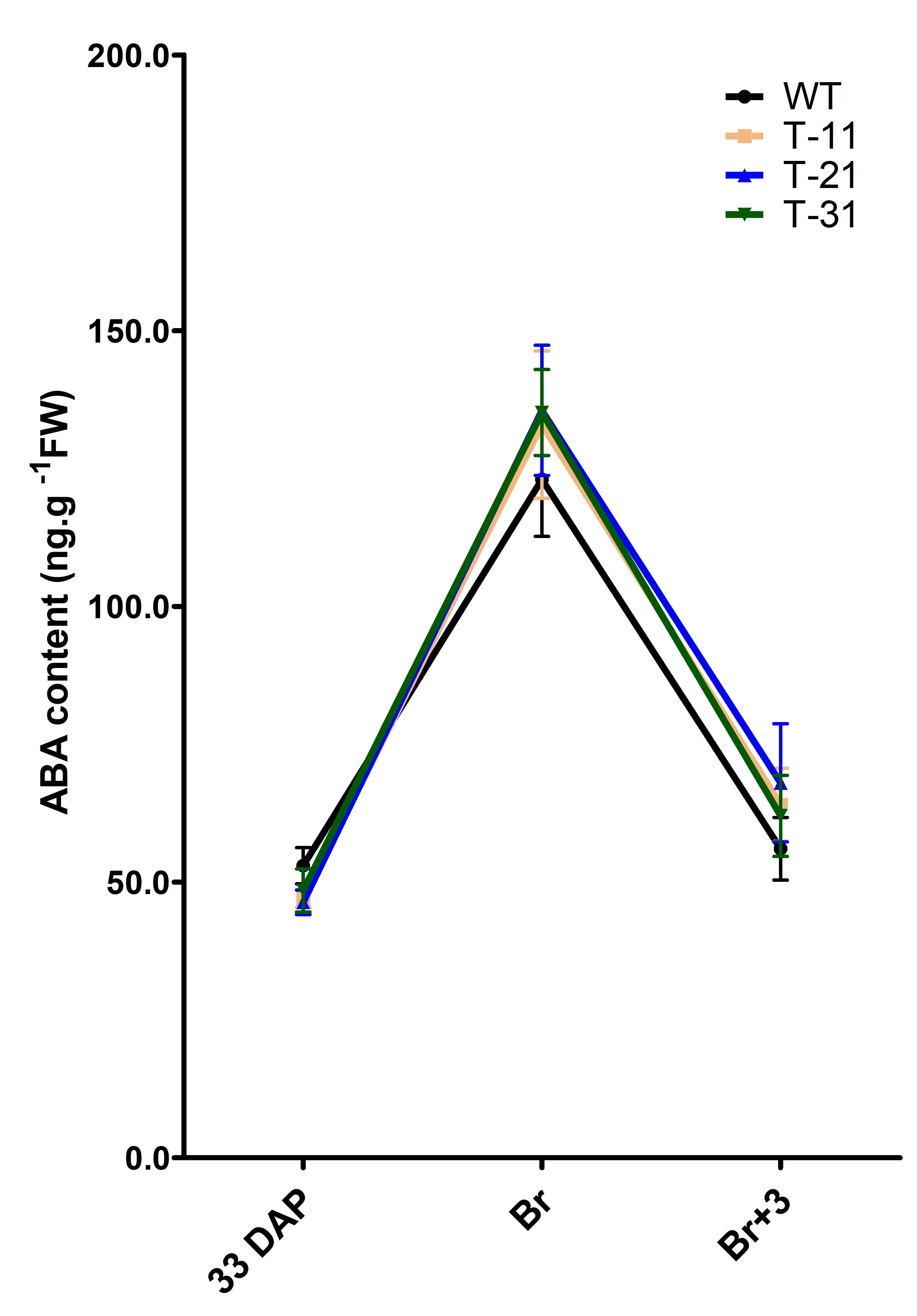


**Supplementary Figure S4 Changes of endogenous ABA content during fruit ripening in tomato.** Fruits harvested at the mature green (MG, 33DPA), breaker (Br), and orange (Br+3) stages.


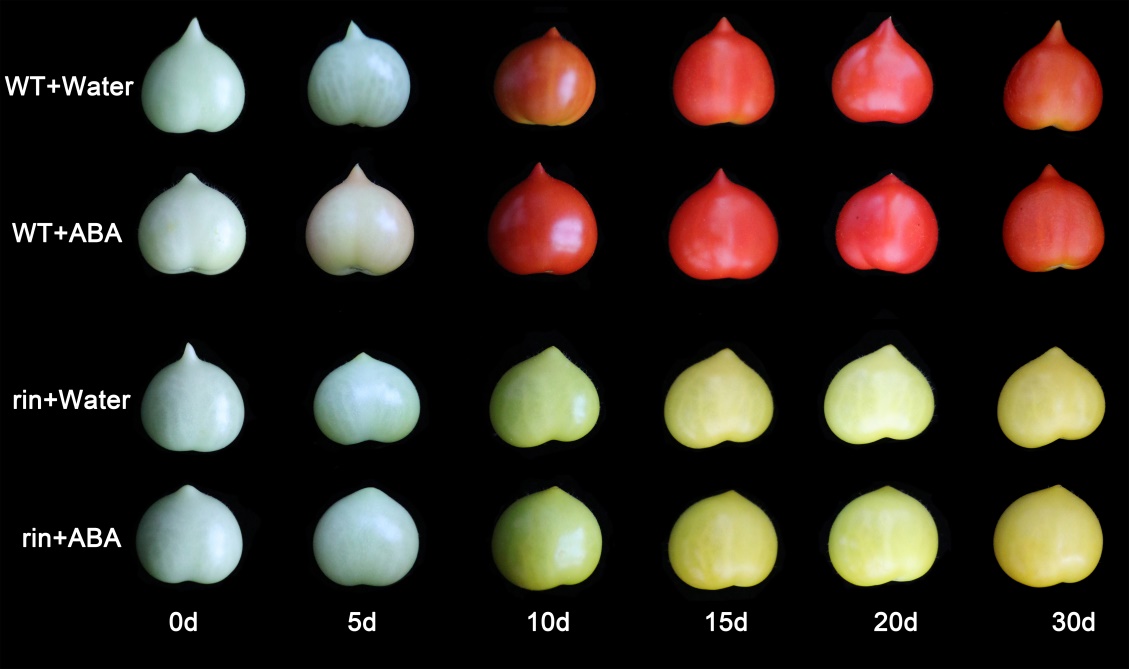


**Supplementary Figure S5 Recovery effect of ABA on fruit ripening in *rin* tomato fruit.** Fruits harvested at the mature green stage (33DPA)

**Supplementary Table S1 Gene ID of ABA receptor genes**

| Gene number | Gene ID in tomato genome database |
| --- | --- |
| *SlRCAR 1* | Solyc08g076960.1.1 |
| *SlRCAR 2* | Solyc06g061180.1.1 |
| *SlRCAR 3* | Solyc12g095970.1.1 |
| *SlRCAR 4* | Solyc08g065410.1.1 |
| *SlRCAR 5* | Solyc10g085310.1.1 |
| *SlRCAR 6* | Solyc03g095780.1.1 |
| *SlRCAR 7* | Solyc10g076410.1.1 |
| *SlRCAR 8* | Solyc09g015380.1.1 |
| *SlRCAR 9* | Solyc01g095700.2.1 |
| *SlRCAR 10* | Solyc06g050500.2.1 |
| *SlRCAR 11* | Solyc12g055990.1.1 |
| *SlRCAR 12* | Solyc03g007310.2.1 |
| *SlRCAR 13* | Solyc08g082180.2.1 |
| *SlRCAR 14* | Solyc02g076770.1.1 |
| *SlRCAR 15* | Solyc05g052420.1.1 |

**Supplementary Table S2 qRT-PCR primers in this study**

| Gene ID of tomato genome database | Primer name | Primer sequence(5’-3’) |
| --- | --- | --- |
| Solyc08g076960.1.1 | Q-SlRCAR1-F | GGAGAGATCTGGACGGTTGT |
|  | Q-SlRCAR1-R | GCGCCAAAGTTTCAGTAACA |
| Solyc06g061180.1.1 | Q-SlRCAR2-F | TGATGTGCCTGAAGGGAATA |
|  | Q-SlRCAR2-R | TCTCACCGTTACCACCAAGA |
| Solyc12g095970.1.1 | Q-SlRCAR3-F | ATTAGTTCCGCAAGGGCTAA |
|  | Q-SlRCAR3-R | CGTTGCGTCACAAGAGAAGT |
| Solyc08g065410.1.1 | Q-SlRCAR4-F | GATGACAGGTGATGGTGGAG |
|  | Q-SlRCAR4-R | GTCGCTCTGTGCTTGTAGATG |
| Solyc10g085310.1.1 | Q-SlRCAR5-F | GGGACCACAAGTTAGCCAAT |
|  | Q-SlRCAR5-R | ATCAACGACGTACGATTCCA |
| Solyc03g095780.1.1 | Q-SlRCAR6-F | TCAGCTGCAGAGAATCAACC |
|  | Q-SlRCAR6-R | GAGAACTGCACCGGGATAAT |
| Solyc10g076410.1.1 | Q-SlRCAR7-F | GTCAAGTATCACGCGCATCT |
|  | Q-SlRCAR7-R | TATACGCTTGAGGGTTGTCG |
| Solyc09g015380.1.1 | Q-SlRCAR8-F | TCAGCTTCAGTGTCGTAGGC |
|  | Q-SlRCAR8-R | AGCTTTGATCGGAGTGGAGT |
| Solyc01g095700.2.1 | Q-SlRCAR9-F | GCTATGCAGGGAGGTGTTCT |
|  | Q-SlRCAR9-R | CAAGCAGAAGCAAATTCCAA |
| Solyc06g050500.2.1 | Q-SlRCAR10-F | CGCAAGCGTACAAACACTTT |
|  | Q-SlRCAR10-R | AAATCTCCAGCCTCTCCGTA |
| Solyc12g055990.1.1 | Q-SlRCAR11-F | GACACCATAGGCATGTGCTC |
|  | Q-SlRCAR11-R | TTGCACGACACATCTGCTAA |
| Solyc03g007310.2.1 | Q-SlRCAR12-F | AGTCAAGCACATTCGAGCAC |
|  | Q-SlRCAR12-R | ATCCCAAGATCTCCTTGCAC |
| Solyc08g082180.2.1 | Q-SlRCAR13-F | AAGGCCTGGAACAATGGTAG |
|  | Q-SlRCAR13-R | TTTGAGGTTACACCGGATGA |
| Solyc02g076770.1.1 | Q-SlRCAR14-F | CCAAATGCATACGTCCAATC |
|  | Q-SlRCAR14-R | AGTGGTGCATCGATGACTTT |
| Solyc05g052420.1.1 | Q-SlRCAR15-F | ACACTCCAACTCCACCGAAT |
|  | Q-SlRCAR15-R | GGGTGGGTATGGTAATGGAA |
| Solyc07g064130.1.1 | Q-SlUBI-F | GCCGACTACAACATCCAGAAGG |
|  | Q-SlUBI-R | TGCAACACAGCGAGCTTAACC |
| Solyc01g101060.2.1 | Q-SlSAMS1-F | TGATGGCAAGACCCAAGTTA |
|  | Q-SlSAMS1-R | CATTGGTAACGGTCTCATCG |
| Solyc01g095080.2.1 | Q-SlACS2-F | ATCCACCTTGTTTGTGACGA |
|  | Q-SlACS2-R | TGTTCATCGAGGATTTCAGC |
| Solyc07g049530.2.1 | Q-SlACO1-F | GCGCCACTCTATTGTGGTTA |
|  | Q-SlACO1-R | TGCATCACTTCCTGGATTGT |
| Solyc04g079960.1.1 | Q-SlGGPS2-F | CCTTGTATGGATGACGACGA |
|  | Q-SlGGPS2-R | GTACTCGAATGCGAAAGCAA |
| Solyc02g085700.1.1 | Q-SlGGPS-like-F | AGGAGCAAGAAAGCAAGAGC |
|  | Q-SlGGPS-like-R | TCATGACCGGTTCCTTGATA |
| Solyc11g011340.1.1 | Q-SlGEDH-F | GGAATGCACATTGGTGTTGT |
|  | Q-SlGEDH-R | CAAGAAAGAGTCTGCACCCA |
| Solyc07g032740.2.1 | Q-SlGOT1-F | TGATAGCAGCCCAGTCAAAG |
|  | Q-SlGOT1-R | TTTGTTCAGCTCGTCTCACC |
| Solyc10g076510.1.1 | Q-SlPDC1-like-F | TTGCTTTCATTGGTGATGGT |
|  | Q-SlPDC1-like-R | TTGTGTAACCGCCATTGTTT |
| Solyc05g014790.2.1 | Q-SlLOX6-F | TTGCGGAGTCTTATGTGAGG |
|  | Q-SlLOX6-R | ATCGTGTGGCACAGTCAAAT |
| Solyc06g059740.2.1 | Q-SlADH2-F | GCCTTGGAGCAAGTTTGAAT |
|  | Q-SlADH2-R | AATTATCCTCGAGGCACCAG |
| Solyc01g101060.2.1 | Q-SAMS1-F | CACGGCCATCTGACCAAACG |
|  | Q-SAMS1-R | TTGCCATCAGGCCTCAACCA |
| Solyc12g099000.1.1 | Q-SAMS2-F | CCTCAACCCTTCAGGCCGTT |
|  | Q-SAMS2-R | AGAAAGCACCACCTCCGTGA |
| Solyc10g083970.1.1 | Q-SAMS3-like-F | GGTGGACAGGAGTGGTGCTT |
|  | Q-SAMS3-like-R | ACAGAGAGTGGTTCTGCGACA |
| Solyc09g008280.1.1 | Q-SAMS3-F | ACCAGATCGCCCAGGACTTG |
|  | Q-SAMS3-R | AGCGACCTGATGGGTTGAGG |
| Solyc08g008100.2.1 | Q-ACS6-F | GCAGCAACCGCGTTTAGTCA |
|  | Q-ACS6-R | ACGTGCAATATTGACAACGGCAT |
| Solyc08g081550.2.1 | Q-ACS1A-F | TGCAAACATGGATGACGAGACG |
|  | Q-ACS1A-R | CGGAGAGTGAGCTGGTGAGT |
| Solyc01g095080.2.1 | Q-ACS2-F | CGTCTCGCCTGGATCTTCGT |
|  | Q-ACS2-R | TTCCATCGAACTCGATTTATCTCCACT |
| Solyc05g050010.2.1 | Q-ACS4-F | AGCACAATGGAAGAGGAACAACCT |
|  | Q-ACS4-R | CACGAGCCTGGGCGAATCTA |
| Solyc02g036350.2.1 | Q-ACO-F | TCACACAGATGCTGGTGGCA |
|  | Q-ACO-R | GCTGAGCAATCACCCTGTGC |
| Solyc07g049550.2.1 | Q-ACO4-F | CCACCATGTCCTAAGCCCGAT |
|  | Q-ACO4-R | TAGAGTGGCGCATGGGAGGA |
| Solyc07g049530.2.1 | Q-ACO1-F | CCTCCCATGCGCCACTCTAT |
|  | Q-ACO1-R | CGTGTCCCGTCTGTTTGTGC |
| Solyc07g026650.2.1 | Q-ACO5-F | GTGAGAACCTTGGCCTAGCGA |
|  | Q-ACO5-R | TCCAGACCTGGAACTTGTTCGT |
| Solyc02g081190.2.1 | Q-ACO4-like-F | CTTCGCGCTCACACGGATG |
|  | Q-ACO4-like-R | AGCTGATCGCCGAGGTTGAT |
| Solyc08g014420.2.1 | Q-MAPK-F | ACGGATACGGTGATGTCGGATG |
|  | Q-MAPK-R | ATAAACCGGCCACCGTGACT |
| Solyc12g011330.2.1 | Q-ETR1-F | TCGCAAGACTTCGACCTGCT |
|  | Q-ETR1-R | GCTCCAGCTCATGAACATGCC |
| Solyc07g056580.2.1 | Q-ETR-F | TGGGTGCTTGTGCAGTTTGG |
|  | Q-ETR-R | AAGCATGACAGCAGTTGCACA |
| Solyc09g075440.2.1 | Q-ETR3-F | CGCAGATCAGGTTGCTGTCG |
|  | Q-ETR3-R | GCATGCATGGGCGTTCTCAT |
| Solyc06g053710.2.1 | Q-ETR2-F | ACCGAGCTCTGGAACAAGCA |
|  | Q-ETR2-R | AGCCGCTGCTCATTACCCAA |
| Solyc11g006180.1.1 | Q-ETR5-F | GTGCTCTGGGCCCTTCACTA |
|  | Q-ETR5-R | GGGCTATGATCAACGGCCAAC |
| Solyc09g089610.2.1 | Q-ETR6-F | TCCAATGCTGACGGTCTCGAA |
|  | Q-ETR6-R | ACCACTGCAGCATGGGACAA |
| Solyc10g083610.1.1 | Q-CTR1-F | TTTCGGGTACCGGGTTCGAT |
|  | Q-CTR1-R | CGAGCCACAGCCCTCATTCT |
| Solyc03g123800.1.1 | Q-MAPKK-F | GGCACCACCCAGTGCTTCTA |
|  | Q-MAPKK-R | GCCGGTGCTATTCTGGGTGA |
| Solyc09g007870.2.1 | Q-EIN2-F | ACCTGGCATCCGTCCTCAAG |
|  | Q-EIN2-R | GGTGATGAGGCAGACAGCGT |
| Solyc06g073730.1.1 | Q-EIN3-like(EIL4-F | GCAGAGCAGGTGGTGGATGA |
|  | Q-EIN3-like(EIL4-R | CCATCCTGTGCCCTCGACAT |
| Solyc06g073720.1.1 | Q-EIL1-F | TCTTCCCTGCTCCGCTGAAG |
|  | Q-EIL1-R | TCCCTCCACATCCTCCTCTCC |
| Solyc01g009170.2.1 | Q-EIL2-F | ACAGTATGCTGAGCCAAAGCG |
|  | Q-EIL2-R | ATCACCCTCTGCCCATCTGC |
| Solyc01g096810.2.1 | Q-EIL3-F | CACAGAGGCGGTTTCCGTTG |
|  | Q-EIL3-R | TTACCGCCGTCAGAACACCA |
| Solyc01g006650.1.1 | Q-EN3-like3-F | CAAGCTCAAGCGGCTCAAGG |
|  | Q-EN3-like3-R | TCCTCGAGCATTGCAGACCT |
| Solyc05g051200.1.1 | Q-ERF1-F | CAAGAAGAGGTAACATCCATAGAG |
|  | Q-ERF1-R | TCAAATGTTCCTAACCAAACCCTA |
| Solyc02g077370.1.1 | Q-PTI5-F | CGCGATTCGGCTAGACATGG |
|  | Q-PTI5-R | AGTGCCTTAGCACCTCGCAT |
| Solyc08g078190.1.1 | Q-ERF-like-F | TGCAATTGAAGCGGCGAAGG |
|  | Q-ERF-like-R | TCAGTTTCCACCGCTTCTCTCC |
| Solyc03g093540.1.1 | Q-ERF1A-F | CAGCGGAGATTCGTGACCCA |
|  | Q-ERF1A-R | TTTGCAACTTCGAGTGGGAAATTCA |
| Solyc12g009560.1.1 | Q-EBF1-F | AGCAGAGAATTGCCCTAGCTTGA |
|  | Q-EBF1-R | GCACCTGATGACAGAAGACTAGCA |
| Solyc08g060810.2.1 | Q-EBF2-F | TCTCGTGTTGCGGTCCCTTT |
|  | Q-EBF2-R | AAGCCAGCGCTTGGAAACAC |
| Solyc03g007960.2.1 | Q-CrtR-b2-F | GATCGTCCCTGGCCTCTGTT |
|  | Q-CrtR-b2-R | GCTGCAGCTACCCTCCGAAA |
| Solyc06g036260.2.1 | Q-CrtR-b1-F | CACTTTCACTCTCACGCACACA |
|  | Q-CrtR-b1-R | TGAGGTAGAGGAGGCGGAGA |
| Solyc02g090890.2.1 | Q-ZEP(ABA1)-F | TGGTTCTGATAAGGCAGCATTTCG |
|  | Q-ZEP(ABA1)-R | GCTCAACGCCTGATGTTTGCT |
| Solyc07g056570.1.1 | Q-NCED1-F | CCGACCCACGAGTCCAGATT |
|  | Q-NCED1-R | GAACGGCGTGAACCATACCG |
| Solyc08g016720.1.1 | Q-NCED2-F | CACGTGTACAAATCGCCGGAA |
|  | Q-NCED2-R | CGGGTTCGCTCCATTCCTGA |
| Solyc04g071940.2.1 | Q-(ABA2-like)-F | GGCAAGTGCCATTGGTGGAG |
|  | Q-(ABA2-like)-R | TTCCCAACTCAGCTGCAACG |
| Solyc01g088230.2.1 | Q-TAO4-F | CCACCACCAGACTCGAGGAC |
|  | Q-TAO4-R | ATCAGCCCGGAAAGCAGCAT |
| Solyc01g088220.2.1 | Q-TAO4-like-F | TGGAGGTGGACTCGGTCAGA |
|  | Q-TAO4-like-R | ACACGATTGGTACTCGGGAGA |
| Solyc11g071600.1.1 | Q-TA03-F | CCAGTCGGTGAACCGATGAAGA |
|  | Q-TA03-R | ATGCAGGCAGTTTGGTGGTG |
| Solyc11g071610.1.1 | Q-AO1-F | GCTCCACGACCTCATGGGAA |
|  | Q-AO1-R | TGTGCCATACGCACCAAACG |
| Solyc11g071620.1.1 | Q-AO1-like-F | TGGCAGTGTCGTCCAGCTTT |
|  | Q-AO1-like-R | GCCCTTGGCCAACATCAACC |
| Solyc01g088170.2.1 | Q-TA02-F | TGAACCGATGAAGAAGGTCGGA |
|  | Q-TA02-R | TACAGGCAGTTTGGCGGTGA |
| Solyc01g108280.2.1 | Q-SnRK2E-F | GCCAGATCAACGTATGCAGAGC |
|  | Q-SnRK2E-R | AGTGAGGTAATGATTAAGGCTGTTGGT |
| Solyc02g090390.2.1 | Q-SnRK2I-F | GGCGCTTCAATGAGGATGAGG |
|  | Q-SnRK2I-R | TGCAGCAGAGCAGACTTGGA |
| Solyc08g077780.2.1 | Q-SAPK3-F | ATGAGCGCCCAACACTCCAT |
|  | Q-SAPK3-R | TCTCGCGTGCTCAACATCCA |
| Solyc01g103940.2.1 | Q-SnRK2B-F | AGGCACCTTCTCTCTCGCAT |
|  | Q-SnRK2B-R | GCCTGTGCTGCTTCTGTCAA |
| Solyc04g074500.2.1 | Q-SAPK2-F | GAGGTCTTGCTTACACCTACTCATCT |
|  | Q-SAPK2-R | GCATGAAATGGCAGTAGCTAACCC |
| Solyc04g012160.2.1 | Q-SnRK2C-F | AAAGATAGCAGATGTTTGGTCTTGTGG |
|  | Q-SnRK2C-R | CACTCCTTGGAGACTCGGACA |
| Solyc01g108080.2.1 | Q-ABI5-like7-F | CGGCCTCACAGCAACAACAA |
|  | Q-ABI5-like7-R | TCCGCCTTGAGCCATACTAGC |
| Solyc04g078840.2.1 | Q-AREB-F | GCAGCCTGCTTTGCCCTATG |
|  | Q-AREB-R | CCCACTGGCTCCTAAACCTACC |
| Solyc11g044560.1.1 | Q-SLABF4-F | AGTCTGCGGCCAGATCAAGG |
|  | Q-SLABF4-R | CGTCACTGTCCTTCGCAAGC |
| Solyc10g081350.1.1 | Q-ABI5-like2(1)-F | TGGGCACATTATCGGACACACA |
|  | Q-ABI5-like2(1)-R | TCCAGCTCATGGGTGTAAGCC |
| Solyc01g008980.2.1 | Q-ABI5-like2(2)-F | GCTTGTCACCATCTCCTTCGC |
|  | Q-ABI5-like2(2)-R | TGACACCTTGTTCACCAGCTCA |
| Solyc10g076920.1.1 | Q-ABI5-like2(3)-F | GCCAGGCCATCCAATTCAACA |
|  | Q-ABI5-like2(3)-R | TCATCCGCTTCTGCCTCCTT |
| Solyc05g052410.1.1 | Q-DREB2A-F | ATGGAAGGAAGCCAGTGCGT |
|  | Q-DREB2A-R | CCCAACCAAAGCCTTCTCCCT |
| Solyc10g076370.1.1 | Q-DREB2A-like-F | GCTGAAATTCGCGAGCCTGT |
|  | Q-DREB2A-like-R | CAGCAGCATCACCAGCAGTC |
| Solyc10g080210.1.1 | Q-SlPG-F | GGGCACAAGTGCAACAAAGGT |
|  | Q-SlPG-R | TGCACGTAGCCTCTGATGGT |
| Solyc08g060970.2.1 | Q-SlTPGL-F | TGATTCACCGGCACAGCCATA |
|  | Q-SlTPGL-R | GCTTGGCCCTCCATAGCTGA |
| Solyc04g082140.2.1 | Q-SlPE-F | GCACGACAGTACCTCGGTCA |
|  | Q-SlPE-R | GTGCCGATTAGCCACCTTGC |
| Solyc05g005560.2.1 | Q-SlPGbeta-F | TCTTCCACGCCGGTGATGAG |
|  | Q-SlPGbeta-R | CGACGACGTTTCGACCCAAC |
| Solyc08g081620.2.1 | Q-SlCel1-F | GCAACGATGGCTATAGTTCATGGTACA |
|  | Q-SlCel1-R | GCGGCCACGGACCCTATAAA |
| Solyc09g010210.2.1 | Q-SlCel2-F | ACCGCCGGATCAACGTATCA |
|  | Q-SlCel2-R | TCGTCGTAAACGCCATCGGA |
| Solyc09g075360.2.1 | Q-SlCel4-F | TGGGACTGAAGTTGCTGCTGA |
|  | Q-SlCel4-R | AAGGCAAACACCCTGATGGC |
| Solyc08g083210.2.1 | Q-SlCel5-F | CCAGGTTCTGATGTTGCTGCTG |
|  | Q-SlCel5-R | TGGGCAGACCACTGAACTGA |
| Solyc06g051800.2.1 | Q-SlExp1-F | GCGTTGCCAAATGACAATGGTG |
|  | Q-SlExp1-R | TCCTTGCTTTCGGCATGGGA |
| Solyc09g092520.2.1 | Q-SlXET1-F | TGCAAATCAAACTCGTTGCTGGA |
|  | Q-SlXET1-R | TGTTACCCTTGCCTTGGGCAT |
| Solyc07g009380.2.1 | Q-SlXET2-F | ACTTCCAAGAATCCCACAGCCA |
|  | Q-SlXET2-R | AGGGAACCCTTGAGGGAAACG |
| Solyc11g065600.1.1 | Q-SlXET4-F | TCTGCTGGTGTTGTCACTGCT |
|  | Q-SlXET4-R | TGTCTCCTTTGCCTCCTGTGA |
| Solyc07g052980.2.1 | Q-SlXET16-F | AGGCGGGCTCGTTAAGACTG |
|  | Q-SlXET16-R | AGCTGGTGACTCTGGTGCAA |
| Solyc12g017240.1.1 | Q-SlXETB1-F | TGGTGATCAGCGAGCTCAGA |
|  | Q-SlXETB1-R | CGTGCCCTGCTCCTTGAGAA |
| Solyc07g056000.2.1 | Q-SlXETB2-F | ACCAGCGCGCTCAGATACAA |
|  | Q-SlXETB2-R | TGGTGACAGTGCCAGCAGAA |
| Solyc01g099630.2.1 | Q-SlXETm-F | GCCAAGTTGGGCTAGTCACC |
|  | Q-SlXETm-R | CACCAGCTGAGTCTCCACCA |
| Solyc12g044880.1.1 | Q-SlTBG1-F | TGCTGGTGTTCTTGGACCAGT |
|  | Q-SlTBG1-R | GCTGCTTCTGAGCCACTAAAGAG |
| Solyc09g092160.2.1 | Q-SlTBG2-F | GGCTGTTGGTCTTGGTGCTG |
|  | Q-SlTBG2-R | CTCGGAATTCGGCGTGAACC |
| Solyc03g121540.2.1 | Q-SlTBG3-F | TGTGCAAGCAAGACGATGCC |
|  | Q-SlTBG3-R | GCAGTCCAGGCTTCAGTCCA |
| Solyc12g008840.1.1 | Q-SlTBG4-F | ACGCGCCTGGAGGAAATGAT |
|  | Q-SlTBG4-R | TTTGCTGCAGTCGCCTTGTG |
| Solyc11g069270.1.1 | Q-SlTBG5-F | CGTTTGGTGGTCCTGTCCCT |
|  | Q-SlTBG5-R | ACGGTCCACCACTGGTTCTC |
| Solyc02g084720.2.1 | Q-SlTBG6-F | TGGTCAGCTCTCAGGTTCTGC |
|  | Q-SlTBG6-R | AGTACGCCAGTGCTCCATGT |
| Solyc03g019890.2.1 | Q-SlTBG7-F | TCGTCGTCGTTAGCAGCAGT |
|  | Q-SlTBG7-R | TCGCCAATCGAACCAGACCA |
| Solyc03g093130.2.1 | Q-SlXTH3-F | TGATTGGAGCCAAGCACCCT |
|  | Q-SlXTH3-R | TGCACCCATTTGAGCCTCTCT |
| Solyc12g011030.1.1 | Q-SlXTH9-F | GGGCGAAGGACGCGGTAA |
|  | Q-SlXTH9-R | TGGATTGAAAGCCAGAGCCAGA |
| Solyc07g064190.1.1 | Q-PE3-F | CCTTCCGCTCTGCCACTCTT |
|  | Q-PE3-R | AGCTCCAACTCGAAGTGCCA |
| Solyc12g008530.1.1 | Q-PE53-F | GCACAAACTATTGGCCCAAATGGA |
|  | Q-PE53-R | TGCAACCTACAAAGGCTGCTG |
| Solyc01g099960.2.1 | Q-PE22-F | ATCACGGCCCAAGGTCGAAA |
|  | Q-PE22-R | GGGCTGGACCATTCCACTCA |
| Solyc12g096730.1.1 | Q-TPG6-F | TGCGTATCCATTGGTCCAGGAA |
|  | Q-TPG6-R | TCCTTCGCTAAGCTTCCAATGC |

**Supplementary Table S3 Primers of multi-gene interference vector construction.**

| Vector of multi-gene interference | Primer name with restriction sites | Primer sequence (5’-3’) | Primer for overlap PCR | Primer sequence (5’-3’) |
| --- | --- | --- | --- | --- |
| pCAMBIA1301:35:RCAR1-2-3-4 | RCAR1-F | ACGCGTCGACTCTAGAGTATCATTGGCGGAGAACACC | ABA1-R-B | GGTTCGGTTCCTCGCATCACGACTATTAACACTA |
|  | RCAR2-R | TCCCCCGGGGAGCTCGGTAAACCGGAGATGACGTTG | ABA2-F-B | GTGATGCGAGGAACCGAACCCGACCCAT |
|  | RCAR3-F | ACGCGTCGACTCTAGATAGACAATTATTAGTTCCGCAAGG | ABA3-R-B | GTCATCTTGCCTACCAACTCCGCCATCAC |
|  | RCAR4-R | TCCCCCGGGGAGCTCGGTGTCTTCTCCAGTATTCCCT | ABA4-F-B | GAGTTGGTAGGCAAGATGACAGGTGATGGTG |
| pCAMBIA1301:35:RCAR5-7-8 | RCAR5-F | ACGCGTCGACTCTAGAGCCTCCAAGTTCTTCAGATTCAT |  |  |
|  | RCAR5-R | TCCCCCGGGGAGCTCCTTATACGCCTGCGGATTGTC |  |  |
|  | RCAR7-F | ACGCGTCGACTCTAGAACATCACCACACACCAATACCAT | ABA7-R-B | TGGTTGTGGTGGAGATAACTCGGACTTCACG |
|  | RCAR8-R | TCCCCCGGGGAGCTCACCCGCACTTCACGGACAG | ABA8-F-B | AGTTATCTCCACCACAACCACCACCAGCAC |
| pCAMBIA1301:35:RCAR6-10-14-15 | RCAR6-F | ACGCGTCGACTCTAGACTTCCACAAACCGCCTCAAC | ABA6-R-B | AGTGGTAACCCTGAACCTCCCTTAATGTGCC |
|  | RCAR10-R | TCCCCCGGGGAGCTCGTTTCTTCCTTCGTATTCCCTTG | ABA10-F-B | GGAGGTTCAGGGTTTCCACTTCTGTCCCTGAT |
|  | RCAR14-F | ACGCGTCGACTCTAGATTTACTCAAACCCCAAATGCATAC | ABA14-R-B | GTTGGAGTGTTACCAACACCACCTTCACCAG |
|  | RCAR15-R | TCCCCCGGGGAGCTCCGCTCAATGCTCGAAGCC | ABA15-F-B | GGTGTTGGTAACACTCCAACTCCACCGAATC |
| pCAMBIA1301:35:RCAR9-12-11-13 | RCAR9-F | ACGCGTCGACTCTAGATAAGCCATTTATTAGCAGGTGTA | ABA9-R-B | TCTGTGGCTGCAAGTCTCCTCTTTAGTGTTCC |
|  | RCAR12-R | TCCCCCGGGGAGCTCTGTCCCAGGTCTTCCATCAATC | ABA12-F-B | AGGAGACTTGCAGCCACAGAAGTACAAGCCC |
|  | RCAR11-F | ACGCGTCGACTCTAGATGACGAGGAACACATCTTT | ABA11-R-B | TTCAAGGTCCCTAGATTCTATCAATAGGGTCGGTA |
|  | RCAR13-R | TCCCCCGGGGAGCTCTAACAAGTTTCATCCTTAGTATTCCC | ABA13-F-B | TAGAATCTAGGGACCTTGAAATCGGAAGTGTT |

**Supplementary Table S4 Primers of transgenic positive detection for the multiple-gene silencing vectors**

| Primer name | Sequence(5’-3’) |
| --- | --- |
| 35s-F | CCTTCCTCTATATAAGGAAG |
| LOOP-F | ACAAGTTCAGCGTGTCCGGCGA |
| Nos-R | CCAAATGTTTGAACGATCGG |
| RCAR1-F(RNAi-S-R/RNAi-R-F) | ACGCGTCGACTCTAGAGTATCATTGGCGGAGAACACC |
| RCAR3-F(RNAi-S-R/RNAi-R-F) | ACGCGTCGACTCTAGATAGACAATTATTAGTTCCGCAAGG |
| RCAR5-F(RNAi-S-R/RNAi-R-F) | ACGCGTCGACTCTAGAGCCTCCAAGTTCTTCAGATTCAT |
| RCAR7-F(RNAi-S-R/RNAi-R-F) | ACGCGTCGACTCTAGAACATCACCACACACCAATACCAT |
| RCAR6-F(RNAi-S-R/RNAi-R-F) | ACGCGTCGACTCTAGACTTCCACAAACCGCCTCAAC |
| RCAR14-F(RNAi-S-R/RNAi-R-F) | ACGCGTCGACTCTAGATTTACTCAAACCCCAAATGCATAC |
| RCAR9-F(RNAi-S-R/RNAi-R-F) | ACGCGTCGACTCTAGATAAGCCATTTATTAGCAGGTGTA |
| RCAR11-F(RNAi-S-R/RNAi-R-F) | ACGCGTCGACTCTAGATGACGAGGAACACATCTTT |
| RCAR12-F(RNAi-S-R/RNAi-R-F) | ACGCGTCGACTCTAGACAGCCACAGAAGTACAAGCCC |
| RCAR13-F(RNAi-S-R/RNAi-R-F) | ACGCGTCGACTCTAGAGGACCTTGAAATCGGAAGTGTT |

**Supplementary Table S5 Fruit ripening time of four-gene silenced tomato, including SlRCAR1-2-3-4, SlRCAR5-7-8, SlRCAR6-10-14-15, and SlRCAR9-12-11-13.**

|  | Line | Mean | SD | P value |
| --- | --- | --- | --- | --- |
| WT |  | 42.29 | 5.05 | 1.0000 |
| SlRCAR 1-2-3-4 | T-12 | 42.33 | 4.39 | 0.8285 |
|  | T-25 | 43.39 | 4.71 | 0.2734 |
|  | T-29 | 41.97 | 4.27 | 0.4864 |
| SlRCAR 5-7-8 | T-20 | 37.29 | 3.85 | 0.0001 |
|  | T-32 | 38.86 | 4.15 | 0.0019 |
|  | T-39 | 39.11 | 3.43 | 0.0007 |
| SlRCAR 6-10-14-15 | T-13 | 41.60 | 4.44 | 0.8214 |
|  | T-15 | 42.38 | 4.95 | 0.8439 |
|  | T-18 | 42.97 | 5.34 | 0.5023 |
| SlRCAR 9-11-12-13 | T-11 | 47.60 | 6.33 | 0.0000 |
|  | T-21 | 49.05 | 5.63 | 0.0000 |
|  | T-31 | 46.49 | 4.83 | 0.0000 |

**Supplementary Table S6 Fruit ripening time of single-gene, double-gene, and three-gene silenced tomato.**

|  | Line | Mean | SD | P value |
| --- | --- | --- | --- | --- |
| WT |  | 42.39 | 6.24 | 1.0000 |
| SlRCAR 9 | T-7 | 43.41 | 5.91 | 0.3243 |
|  | T-12 | 43.75 | 5.79 | 0.2918 |
|  | T-18 | 44.46 | 6.33 | 0.1149 |
| SlRCAR 11 | T-9 | 44.39 | 6.94 | 0.0313 |
|  | T-10 | 44.53 | 6.39 | 0.0281 |
|  | T-11 | 43.77 | 6.41 | 0.3103 |
| SlRCAR 12 | T-4 | 42.56 | 5.33 | 0.9000 |
|  | T-6 | 43.46 | 5.52 | 0.3520 |
|  | T-12 | 44.00 | 4.09 | 0.1779 |
| SlRCAR 13 | T-4 | 45.95 | 5.66 | 0.0142 |
|  | T-8 | 43.89 | 5.97 | 0.3225 |
|  | T-11 | 43.26 | 5.67 | 0.4673 |
| SlRCAR 9-11 | T-1 | 44.80 | 7.03 | 0.0175 |
|  | T-2 | 44.22 | 6.98 | 0.1464 |
|  | T-23 | 43.21 | 5.24 | 0.4541 |
| SlRCAR 9-12 | T-4 | 43.54 | 5.50 | 0.3342 |
|  | T-7 | 43.70 | 5.92 | 0.3297 |
|  | T-11 | 43.79 | 5.71 | 0.2420 |
| SlRCAR 9-13 | T-7 | 43.68 | 5.80 | 0.3089 |
|  | T-15 | 43.97 | 6.15 | 0.1823 |
|  | T-16 | 44.44 | 6.36 | 0.0398 |
| SlRCAR 11-12 | T-2 | 44.24 | 6.14 | 0.0488 |
|  | T-12 | 43.71 | 6.42 | 0.1922 |
|  | T-22 | 42.93 | 5.56 | 0.6397 |
| SlRCAR 11-13 | T-6 | 46.14 | 6.43 | 0.0178 |
|  | T-8 | 45.79 | 7.27 | 0.0046 |
|  | T-11 | 45.24 | 7.66 | 0.0120 |
| SlRCAR 12-13 | T-2 | 45.57 | 5.25 | 0.0120 |
|  | T-8 | 44.48 | 5.39 | 0.0843 |
|  | T-13 | 44.24 | 4.94 | 0.0789 |
| SlRCAR 9-11-12 | T-2 | 44.15 | 6.51 | 0.2604 |
|  | T-3 | 45.21 | 6.09 | 0.0682 |
|  | T-9 | 45.67 | 6.80 | 0.0182 |
| SlRCAR 9-11-13 | T-3 | 46.89 | 6.66 | 0.0028 |
|  | T-6 | 46.11 | 7.14 | 0.0061 |
|  | T-15 | 46.24 | 6.79 | 0.0368 |
| SlRCAR 9-12-13 | T-3 | 43.93 | 4.62 | 0.2427 |
|  | T-9 | 45.39 | 6.81 | 0.0350 |
|  | T-11 | 43.65 | 5.56 | 0.3873 |
| SlRCAR 11-12-13 | T-2 | 45.69 | 7.21 | 0.0033 |
|  | T-3 | 45.48 | 5.46 | 0.0177 |
|  | T-13 | 46.18 | 6.18 | 0.0012 |
