# Supplementary material for: Co-silencing of ABA receptors (SlRCAR) reveals interactions between ABA and ethylene signaling during tomato fruit ripening
Source: Hortic Res. 2022 Jun 5;9:uhac057. doi: 10.1093/hr/uhac057 (PMC9171117; doi:10.1093/hr/uhac057)
Supplement: Web_Material_uhac057 [file web_material_uhac057.zip › Supporting information.docx]

**Supporting information**

Additional Supporting Information may be found online in the supporting information tab for this article:

Supplementary Figure S1 Phylogenetic tree of PYR/PYL ABA receptors.

Supplementary Figure S2 Map of multiple-gene silencing system.

Supplementary Figure S3 Repression efficiency detection of ABA receptor genes in transgenic tomato.

Supplementary Figure S4 Changes of endogenous ABA content during fruit ripening in tomato.

Supplementary Figure S5 Recovery effect of ABA on fruit ripening in *rin* tomato fruit.

Supplementary Table S1 Gene ID of ABA receptor genes.

Supplementary Table S2 qRT-PCR primers in this study.

Supplementary Table S3 Primers of multi-gene interference vector construction.

Supplementary Table S4 Primers of transgenic positive detection for the multiple-gene silencing vectors

Supplementary Table S5 Fruit ripening time of four-gene silenced tomato, including SlRCAR1-2-3-4, SlRCAR5-7-8, SlRCAR6-10-14-15, and SlRCAR9-12-11-13.

Supplementary Table S6 Fruit ripening time of single-gene, double-gene and three-gene silenced tomato.

Supplementary appendix 1 Detailed protocols of multiple-genes RNAi vector system

Supplementary appendix 2 Sequence of pcambia1301m and pcambia1301s

Supplementary appendix 3 ABA receptor (PYR/PYL/RCAR) sequences in tomato and other species
